# The aryl hydrocarbon receptor directs the differentiation of murine progenitor blastomeres

**DOI:** 10.1007/s10565-022-09755-9

**Published:** 2022-08-27

**Authors:** Chia-I. Ko, Jacek Biesiada, Hesbon A. Zablon, Xiang Zhang, Mario Medvedovic, Alvaro Puga

**Affiliations:** 1 Department of Environmental and Public Health Sciences and Center for Environmental Genetics, University of Cincinnati, Cincinnati, OH 45267 USA; 2 Center for Biostatistics, 160 Panzeca Way, Cincinnati, OH 45267 USA; 3 Genomics, Epigenomics, and Sequencing Core, 160 Panzeca Way, Cincinnati, OH 45267 USA

**Keywords:** Aryl hydrocarbon receptor, Pluripotency, Preimplantation development, Cellular heterogeneity, OCT4, CDX2

## Abstract

Key regulatory decisions during cleavage divisions in mammalian embryogenesis determine the fate of preimplantation embryonic cells. Single-cell RNA sequencing of early-stage—2-cell, 4-cell, and 8-cell—blastomeres show that the aryl hydrocarbon receptor (AHR), traditionally considered as an environmental sensor, directs blastomere differentiation. Disruption of AHR functions in *Ahr* knockout embryos or in embryos from dams exposed to dioxin, the prototypic xenobiotic AHR agonist, significantly impairs blastocyst formation, causing repression and loss of transcriptional heterogeneity of OCT4 and CDX2 and incidence of nonspecific downregulation of pluripotency. Trajectory—the path of differentiation—and gene variability analyses further confirm that deregulation of OCT4 functions and changes of transcriptional heterogeneity resulting from disruption of AHR functions restrict the emergence of differentiating blastomeres in 4-cell embryos. It appears that AHR directs the differentiation of progenitor blastomeres and that disruption of preimplantation AHR functions may significantly perturb embryogenesis leading to long-lasting conditions at the heart of disease in offspring’s adulthood.

**Supplementary Information:**

The online version contains supplementary material available at 10.1007/s10565-022-09755-9.

## Introduction

Barker’s Theory of the Developmental Origins of Health and Disease (DOHaD) proposes that the environment encountered during fetal life and infancy permanently changes the body’s structure, function, and metabolism and shapes the long-term control of tissue physiology and homeostasis (Fleming et al. 2021). Accordingly, damage resulting from maternal stress, poor nutrition, or exposure to environmental pollutants during fetal life or infancy may be at the heart of adult-onset disease. Preimplantation development is a period of embryogenesis when the embryo is sensitive to environmental conditions; disturbance of this early embryonic environment may induce compensative metabolism in embryos and offspring that could be the cause of later-onset pathological conditions such as large offspring syndrome and cardiovascular disease (Fleming et al. 2004; Velazquez et al. 2016). Consistent with the DOHaD Theory concept, the adverse preimplantation environment may cause developmental alterations in the embryo, triggering a sustained state of insufficiency to increase the risk of disease in the adult.

We have recently found that the AHR, a transcription factor activated by environmental as well as by endogenous agonists, regulates stem cell pluripotency. Untimely AHR activation in pluripotent mouse embryonic stem (ES) cells lengthens mitotic progression, causing pluripotency loss and differentiation failure (Ko et al. 2016). Deletion or inhibition of the AHR accelerates hematopoietic stem cell proliferation, a condition that is useful to generate a stem cell pool for transplant therapy and tissue repair after injury (Angelos et al. 2017; Morales-Hernandez et al. 2017). Yet, however beneficial in that respect, *Ahr* knockout causes premature exhaustion of hematopoietic stem cells, development of a myeloproliferative disorder, and reduction of cardiomyocyte differentiation from ES cells (Wang et al. 2013; Singh et al. 2014). Considering the physiological role of pluripotency factors in stem cells, it is not surprising that AHR and OCT4—one of the three core pluripotency factors—antagonize each other’s expression. Indeed, in mouse ES cells, the pluripotency factors bind to the *Ahr* promoter and repress its expression (Ko et al. 2014). Conversely, in both mouse ES cells and human embryonic carcinoma cells, the AHR triggers differentiation and downregulates the expression of OCT4 and NANOG (Ko et al. 2014, 2016; Morales-Hernandez et al. 2016). Furthermore, in many stem-progenitor cell types, exposure to dioxin downregulates the expression of lineage-specific transcription factors and alters differentiation (Chen et al. 2019; Heo et al. 2019; Watson et al. 2019). Integration of these results suggests that the long-lasting effects resulting from disruption of AHR functions may be due to the dysfunctional regulation of pluripotency.

In vivo pluripotency is a transient state in inner cell mass (ICM) cells surrounded by trophoblasts, the first differentiated lineage resulting from preimplantation development. Individual preimplantation embryonic cells—blastomeres—remain loosely attached to each other until late 8-cell stage, a time when they undergo compaction. During this morphological event, blastomeres acquire not only cell-to-cell adhesion but also a geometric position and polarity within the embryo (White et al. 2016). It has been proposed that the inside-outside position and the polarization along the apicobasal axis of each blastomere define the cell fate (Suwinska et al. 2008). Indeed, the precursor cells of trophoblasts are the polarized outer blastomeres expressing trophoblast markers CDX2 and GATA3, whereas the precursors of pluripotent ICM cells are the inner blastomeres expressing pluripotency factors OCT4, SOX2, and NANOG (Sasaki 2015). Consistent with the observation that totipotency is lost in 4-cell blastomeres, precursors of ICM and trophoblasts can be identified at the 4-cell stage based on their high or low level of OCT4 expression and the mono-methylation of arginine-26 of histone H3 (H3R26me) (Torres-Padilla et al. 2007; Goolam et al. 2016). The concurrence between the emergence of differential cell fates among blastomeres and the upregulation of zygotic OCT4 at this stage suggest that OCT4 initiates blastomere differentiation (Wu and Scholer 2014; Goolam et al. 2016). Although OCT4-low and H3R26me-low 4-cell blastomeres subsequently form trophoblasts, the corresponding trophoblast differentiation only starts at later stages and seems to go along with concerted polarization and compaction (Zenker et al. 2018). Hence, while HIPPO signaling–mediated maintenance of CDX2 expression is responsible for trophoblast differentiation starting at the 16-cell stage, OCT4 seems to control the segregation of early differentiating blastomeres and act to coordinate the cellular events organizing blastomere differentiation (Anani et al. 2014; Wu and Scholer 2014; Hirate et al. 2015; Fukuda et al. 2016). Accordingly, trigger(s) of preimplantation embryonic differentiation may initiate the process by regulating *Oct4* expression and the population of OCT4-expressing cells. In consideration of the AHR expression in embryos up to 8-cell stage and its role in regulation of *Oct4* expression (Peters and Wiley 1995; Dey and Nebert 1998; Jain et al. 1998; Goolam et al. 2016), we hypothesize that a functional AHR is necessary to govern preimplantation development by interacting with OCT4 expression and functions for proper formation of the blastocyst.

We have compared *Ahr*^*−/−*^ and dioxin-exposed *Ahr*^+*/*+^ with *Ahr*^+*/*+^ embryos to examine the involvement of the AHR in preimplantation embryogenesis. Both *Ahr* deletion and its xenobiotic activation by dioxin curtail blastocyst formation, weaken the pluripotent state of ICM, reduce the number of pluripotency factor–expressing ICM cells, and cause the abnormal expression of pluripotency factors in trophoblasts. The absence of 4-cell differentiating blastomeres concurs with the deregulation of OCT4 expression in both *Ahr* knockout and dioxin-exposed embryos, a condition that persists in 8-cell embryos, along with the impaired upregulation of CDX2. Trajectory analyses further indicate that the absence of differentiating blastomeres in these 4-cell embryos is due to the deregulation of OCT4 function and the changes of transcriptional heterogeneity. We conclude that AHR regulates the expression of genes involved in pluripotency control and trophoblast differentiation, notably OCT4 and CDX2, during the commitment of blastomere cell fates. Considering the importance of the cellular states in ICM and trophoblasts to subsequent embryogenesis, disruption of preimplantation AHR functions is likely to cause damage to developmental programs determining the health and disease consequences later in life.

## Results

### AHR regulates the reproductive outcome

**Fig. 1:**
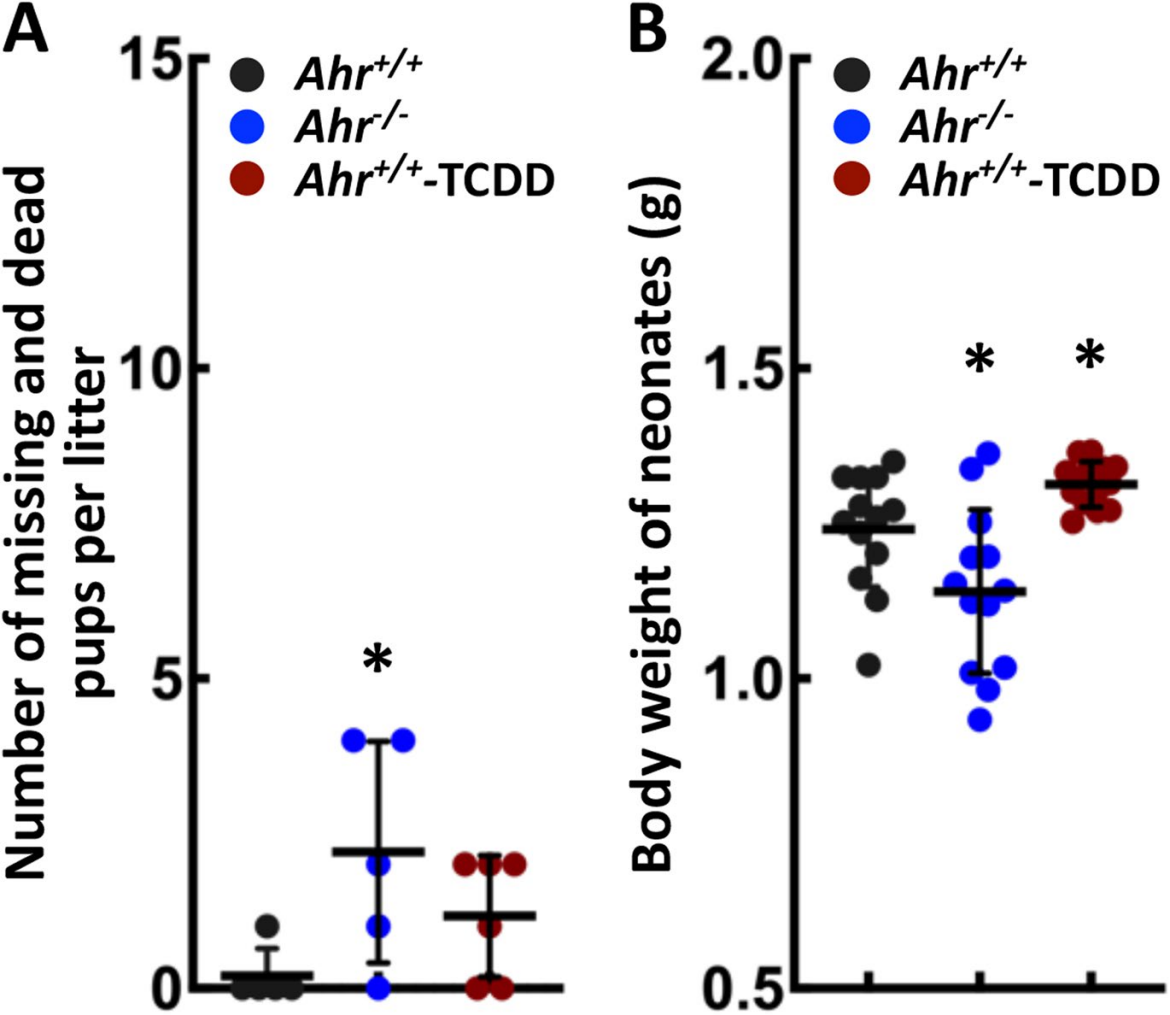
**AHR regulates the reproductive outcome.** The number of missing and dead pups (**A**) and the body weight of neonates (**B**) observed in *Ahr*^+*/*+^, *Ahr*^*−/−*^, and *Ahr*^+*/*+^-TCDD conditions. The numbers of missing and dead pups were determined by subtracting the number of newborns from that of implantation sites obtained from the same dam. Five *Ahr*^+*/*+^, five *Ahr*^*−/−*^, and six *Ahr*^+*/*+^-TCDD litters were examined for missing and dead pups respectively; and body weight of pups were measured within 3 litters of all experimental conditions. Results are shown as the mean ± S.D. * indicates significant difference relative to *Ahr*^+*/*+^ condition at *p-*value < 0.05 obtained from ANOVA followed by a posteriori *t*-test

The C57Bl/6 *Ahr*^*−/−*^ mice suffer from multiple developmental lesions including patent *ductus venosus* that has been associated with long-lasting risk of cardiovascular disease (Lahvis et al. 2000; Lund et al. 2003; Haugen et al. 2005; Tchirikov et al. 2006; Poeppelman and Tobias 2018). Albeit not clear whether the incidence of cardiac disease correlates with patent ductus venosus–related dysfunction, developmental lesions present in *Ahr*^*−/−*^ and dioxin-exposed mice suggest that they might be suitable models to test the DOHaD theory. Considering the role of AHR in pluripotency control, we examined whether *Ahr* deletion or its xenobiotic activation by dioxin during preimplantation development led to abnormal reproductive outcomes. Neonates from wild-type mice exposed to 1 μg/kg dioxin (2,3,7,8-tetrachlorodibenzo-*p*-dioxin; TCDD), the prototypic xenobiotic AHR agonist (hereafter referred to as *Ahr*^+*/*+^-TCDD mice), during preimplantation development and from *Ahr* knockout mice were compared to control *Ahr*^+*/*+^ counterparts for litter size and body weight. Relative to *Ahr*^+*/*+^ mice, we found an increase in *Ahr*^*−/−*^ and a trend to increase in *Ahr*^+*/*+^-TCDD missing and dead pups per litter, respectively (Fig. 1A). Furthermore, we found a significant increase of body weight in *Ahr*^+*/*+^-TCDD neonates, and a decrease in *Ahr*^*−/−*^ neonates relative to *Ahr*^+*/*+^ (Fig. 1B). These observations suggest that the poor reproductive outcomes observed in *Ahr*^*−/−*^ and *Ahr*^+*/*+^-TCDD mice may result from disruption of AHR functions during embryonic development.

### AHR regulates blastocyst formation and the pluripotent state of the ICM

**Fig. 2:**
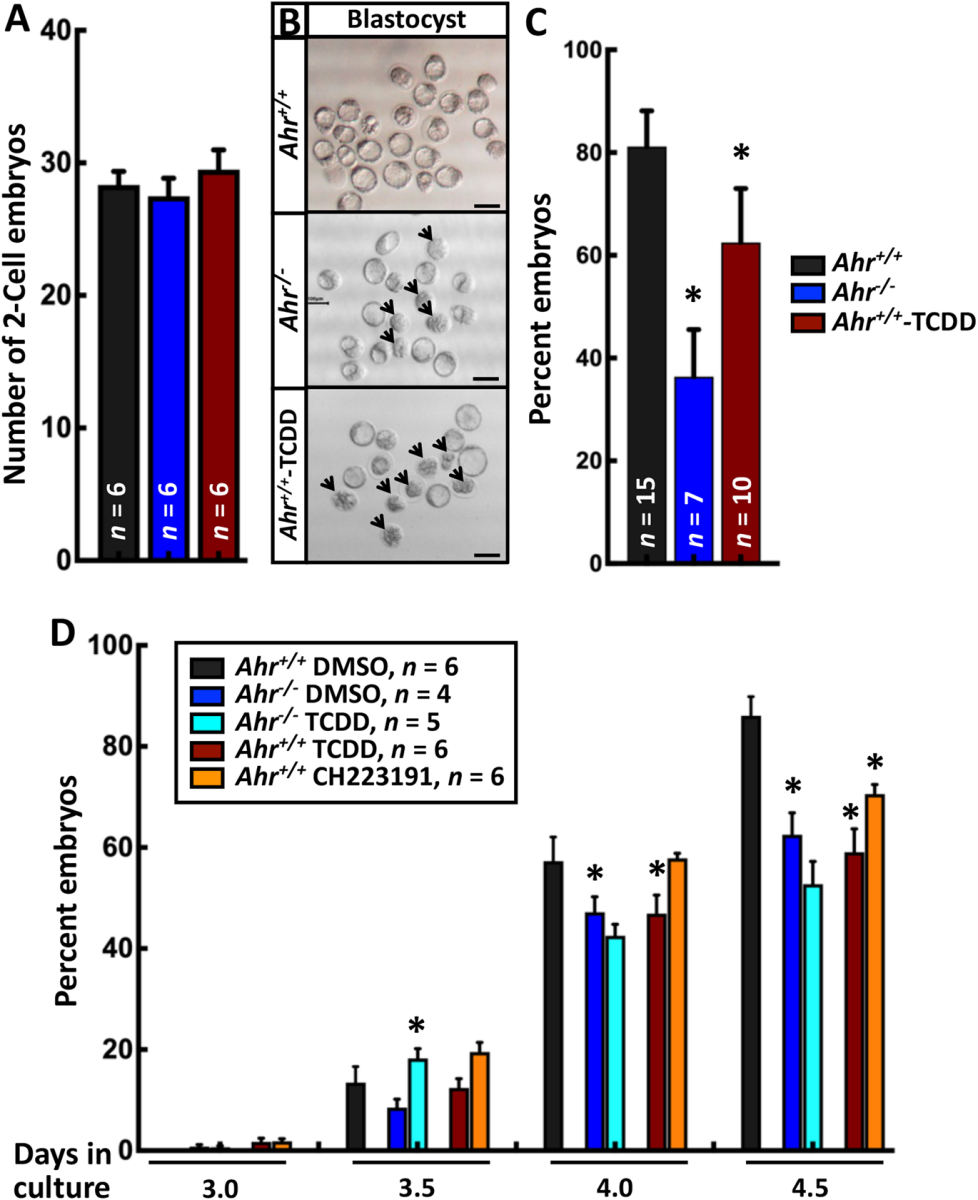
**AHR regulates blastocyst formation.** (**A)** The number of 2-cell embryos observed in *Ahr*^+*/*+^, *Ahr*^*−/−*^, and *Ahr*^+*/*+^-TCDD conditions. (**B)** Morphological image of *Ahr*^+*/*+^, *Ahr*^*−/−*^, and *Ahr*^+*/*+^-TCDD blastocysts. Arrows indicate embryos without blastocele. Scale bar indicates 100 μm. (**C)** The relative number of blastocysts observed in *Ahr*^+*/*+^, *Ahr*^*−/−*^, and *Ahr*^+*/*+^-TCDD conditions. (**D)** The relative number of embryos that have developed to blastocysts in each of the 5 in vitro conditions. Results are shown as the mean ± S.E.M. ***n*** indicates the number of independent litters in each experimental condition. * indicates significant difference relative to control condition, i.e., *Ahr*^+*/*+^ in figures A and C and *Ahr*^+*/*+^ DMSO in panel D, respectively, at *p-*value < 0.05 obtained from ANOVA followed by a posteriori *t*-test

We previously showed that untimely derepression of AHR in ES cells downregulates expression of OCT4 and SOX2 and causes premature loss of pluripotency (Ko et al. 2016). Given the role of OCT4 in organization of cellular events during differentiation of blastomeres (Wu and Scholer 2014; Goolam et al. 2016; Zenker et al. 2018), we hypothesized that interfered AHR functions may alter blastocyst formation and the in vivo pluripotent state of the ICM. To explore the consequences of disrupting AHR functions in blastocysts, *Ahr*^*−/−*^ and *Ahr*^+*/*+^-TCDD embryos were compared to control embryos for possible quantitative and/or morphological differences. While comparable numbers of 2-cell embryos were found in all conditions (Fig. 2A), considerably fewer than control *Ahr*^*−/−*^ and *Ahr*^+*/*+^-TCDD blastocysts were observed with many embryos showing sign of degradation without blastocele (Fig. 2B and 2C). Results consistent with these were obtained when *Ahr*^+*/*+^ and *Ahr*^*−/−*^ embryos were cultured in vitro and exposed to AHR ligands. The number of blastocysts in vehicle-treated *Ahr*^*−/−*^ cultures was significantly decreased relative to *Ahr*^+*/*+^ cultures exposed to control vehicle, as were the numbers in *Ahr*^+*/*+^ cultures treated with the AHR antagonist CH223191 and with TCDD after 4 and 4.5 days (Fig. 2D). As expected, we found no difference between vehicle and TCDD-exposed *Ahr*^*−/−*^ groups, suggesting that effect of TCDD exposure on blastocyst formation is AHR-dependent.

**Fig. 3:**
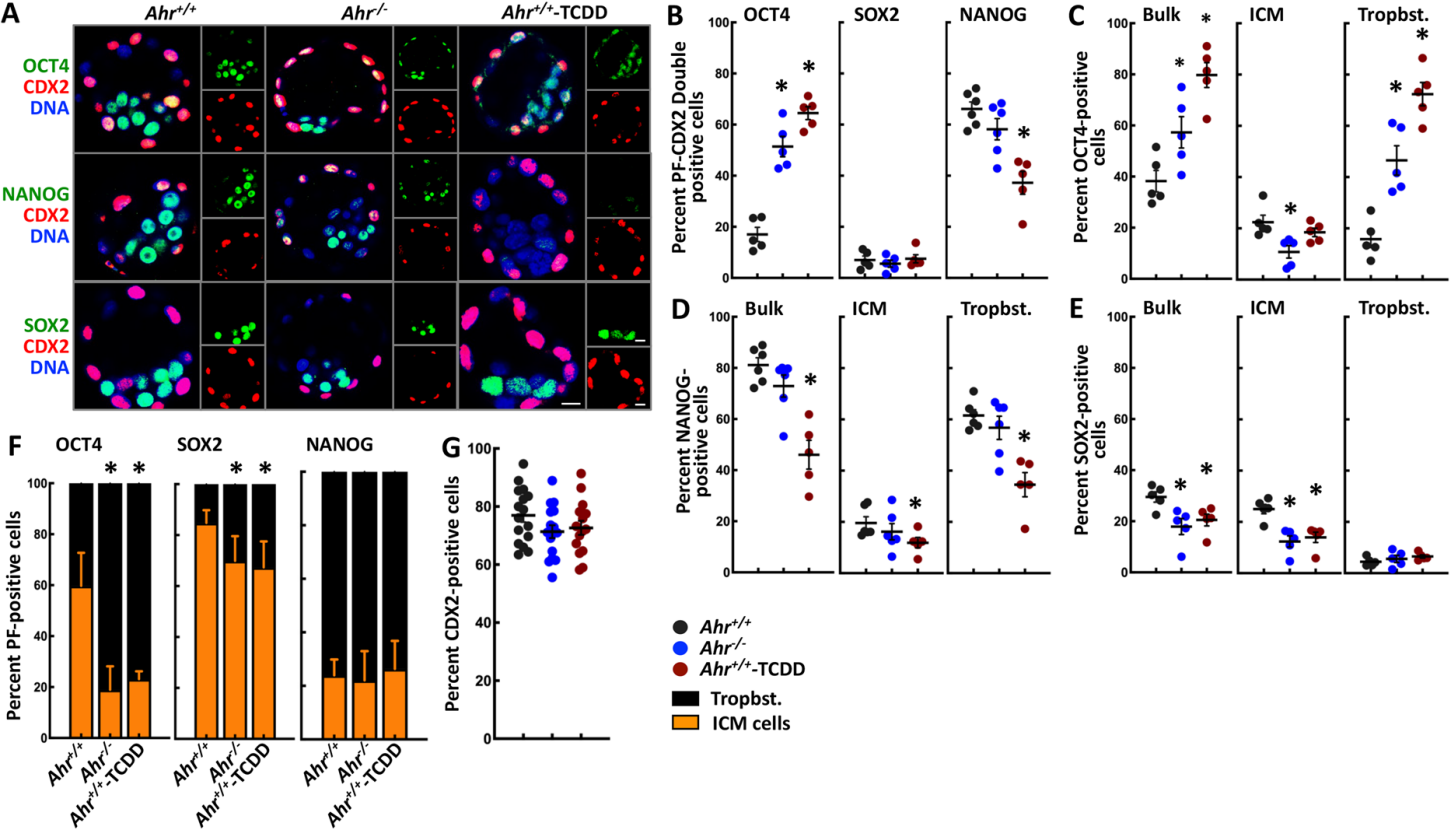
**AHR regulates the pluripotent state in ICM.** (**A)** Immunofluorescence images showing nuclear expression of pluripotency factors OCT4, SOX2, and NANOG and trophoblast marker CDX2 in *Ahr*^+*/*+^, *Ahr*^*−/−*^, and *Ahr*^+*/*+^-TCDD blastocysts. Scale bar indicates 10 μm. (**B**–**G)** Relative number of pluripotency factor and CDX2 double-positive (**B**); the bulk of OCT4-, NANOG-, and SOX2-positive (**C**–**E**); fractional representation of OCT4-, NANOG-, and SOX2-expressing ICM cells and trophoblasts (**F**); and CDX2-positive (**G**) cell count. Results are shown as the mean ± S.E.M. * indicates significant difference relative to *Ahr*^+*/*+^ condition at *p-*value < 0.05 obtained from ANOVA followed by a posteriori *t*-test. PF = pluripotency factors; Tropbst. = trophoblasts

To assess the differentiated state of ICM and trophoblasts in *Ahr*^*−/−*^ and *Ahr*^+*/*+^-TCDD blastocysts, we examined the expression of the pluripotency factors OCT4, SOX2, and NANOG and the trophoblast marker CDX2 by immunofluorescence analyses. *Ahr*^+*/*+^ blastocysts showed nuclear expression of pluripotency factors in ICM and CDX2 in trophoblasts, while abnormal expression of pluripotency factors was observed in *Ahr*^*−/−*^ and *Ahr*^+*/*+^-TCDD trophoblasts (Fig. 3A). Compared to the low number of *Ahr*^+*/*+^ embryonic cells expressing simultaneously OCT4 and CDX2, we found a significantly larger number of OCT4-CDX2 double-positive cells in both *Ahr*^*−/−*^ and *Ahr*^+*/*+^-TCDD blastocysts, and no difference among *Ahr*^+*/*+^, *Ahr*^*−/−*^, and *Ahr*^+*/*+^-TCDD embryonic cells in the number of cells showing SOX2-CDX2 double staining (Fig. 3B). In contrast, the number of NANOG-CDX2 double-positive cells was significantly higher in *Ahr*^+*/*+^ than in *Ahr*^*−/−*^ and *Ahr*^+*/*+^-TCDD blastocysts. When we scored the number of pluripotency factor–expressing embryonic cells and assigned them to either the ICM or the trophoblast lineage based on their position within immunostained blastocysts, we found that changes of OCT4-CDX2 and NANOG-CDX2 double-positive cell numbers in *Ahr*^*−/−*^ and *Ahr*^+*/*+^-TCDD blastocysts followed the same trends observed for the bulk and the trophoblast fraction of OCT4- and NANOG-expressing cells (Fig. 3C and 3D). In addition, we found significantly fewer SOX2-expressing embryonic and ICM cells in *Ahr*^*−/−*^ and *Ahr*^+*/*+^-TCDD than in *Ahr*^+*/*+^ blastocysts, and no difference in the SOX2-expressing trophoblasts (Fig. 3E). Relative to the numbers in *Ahr*^+*/*+^ blastocysts, only OCT4- and SOX2-expressing *Ahr*^*−/−*^ and *Ahr*^+*/*+^-TCDD ICM cells showed a significant decrease, which corresponded to an increase in the numbers of OCT4- and SOX2-expressing trophoblasts (Fig. 3F). We found no difference in the number of CDX2-expressing trophoblasts in all immunostained blastocysts (Fig. 3G). Collectively, disruption of AHR functions seemed to specifically deregulate the level and cell-type specificity of OCT4 and SOX2 expression, suggesting that differentiation was derailed in the *Ahr*^*−/−*^ and *Ahr*^+*/*+^-TCDD embryos.

### AHR directs the segregation of 4-cell blastomeres

**Fig. 4:**
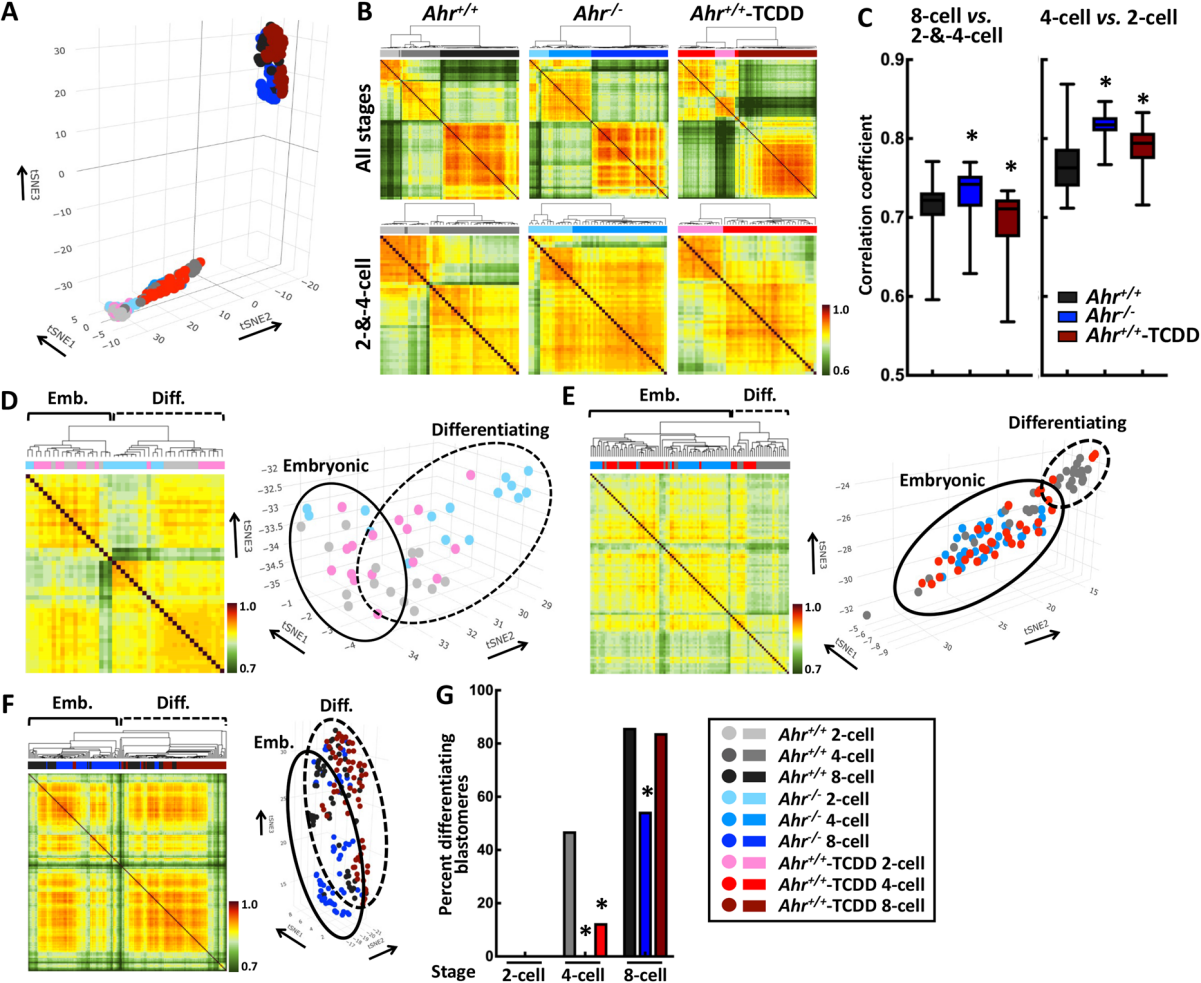
**AHR directs the segregation of 4-cell blastomeres.** (**A)** Three-dimensional tSNE representation of 322 preimplantation single-cell transcriptomes using the most variable genes across all analyzed blastomeres. A pseudotime was assigned to each axis by fitting the developmental stages of blastomeres. (**B)** Cell–cell Pearson’s correlation matrices using all expressed genes across cells of all-stages (top panels) and only 2-cell and 4-cell stages (bottom panels) for *Ahr*^+*/*+^, *Ahr*^*−/−*^, and *Ahr*^+*/*+^-TCDD conditions. We based this analysis on the assumption that the higher the correlation coefficient, the more similar and less heterogeneous the blastomeres should be. Top and right-side colored bars indicate developmental stages and correlation coefficient of blastomeres. (**C)** Correlation coefficients of *Ahr*^+*/*+^, *Ahr*^*−/−*^, and *Ahr*^+*/*+^-TCDD 8-cell relative to 2-and-4-cell and of 4-cell relative to 2-cell stages. (**D**–**F)** Identification of subpopulation in 2-cell (**D**), 4-cell (**E**), and 8-cell (**F**) populations by comparing results obtained from hierarchical clustering on cell–cell Pearson’s correlation matrices to relative location on 3D-tSNE plots. (**G)** Summary of differentiating blastomeres identified in each group shown as percent differentiating blastomeres. * indicates significant difference relative to *Ahr*^+*/*+^ condition at *p-*value < 0.05 obtained from ANOVA followed by a posteriori *t*-test. Emb. = embryonic; Diff. = differentiating

Increasing evidence supports the concept that gene expression heterogeneity is the origin of cell fate decisions (Torres-Padilla and Chambers 2014; Kalkan et al. 2017; Simon et al. 2018). During preimplantation development, the earliest signs of differentiation are observed in 4-cell embryos, where high-*vs*-low levels of OCT4 expression prelude ICM-*vs*-trophoblast cell fates (Torres-Padilla et al. 2007; Goolam et al. 2016). The *Ahr* gene has been identified as one of a group of genes that show significant 4-cell inter-blastomere transcriptional variability (Goolam et al. 2016), suggesting a role for the AHR in the initiation of blastomere differentiation. Accordingly, dysfunctional AHR may disrupt early blastomere differentiation and cause the impaired formation of *Ahr*^*−/−*^ and *Ahr*^+*/*+^-TCDD blastocysts. Since mutual regulation between OCT4 and CDX2 is in place by the 16-cell stage, at a time when *Ahr* expression is undetectable (Peters and Wiley 1995; Jain et al. 1998; Hirate et al. 2015; Fukuda et al. 2016), we aimed specifically at the early 2- to 8-cell stages to explore the potential role of the AHR in regulation of the transcriptome of progenitor blastomeres. We isolated 336 single blastomeres obtained from eight embryos of each of the nine groups: *Ahr*^+*/*+^, *Ahr*^*−/−*^, and *Ahr*^+*/*+^-TCDD each at 2-cell, 4-cell, and 8-cell stages individually, and subjected them to Single-Cell RNA-sequencing (scRNA-seq) allowing the identification of cellular heterogeneity. Three-dimensional t-distributed stochastic neighbor embedding (3D-tSNE) using all variable genes across all cells revealed that blastomeres belonging to 8-cell embryos were distinct from blastomeres of the cluster containing both 2-cell and 4-cell blastomeres (hereafter referred to as the 2-and-4-cell cluster, Fig. 4A). We obtained a similar classification with additional analyses using hierarchical clustering of all expressed genes with expression levels ≥ 1 transcripts-per-million and by clustering cell–cell Pearson’s correlation matrix across all blastomeres analyzed (Supplementary Fig. 1B and 1C). This finding is in agreement with data previously shown by others (Hamatani et al. 2004), attesting to the reliability of our scRNA-seq approach.

To investigate if disrupted AHR function interferes with cellular heterogeneity, we performed cell–cell Pearson’s correlation analyses followed by hierarchical clustering using cells of all three or only of 2-cell and 4-cell stages for *Ahr*^+*/*+^, *Ahr*^*−/−*^, and *Ahr*^+*/*+^-TCDD conditions separately. Similar to the segregation obtained from analyses using all cells, *Ahr*^+*/*+^ 8-cell blastomeres were distinctly grouped when compared to cells belonging to the 2-and-4-cell cluster (Fig. 4B top-left panel). *Ahr*^+*/*+^ blastomeres that belonged to the 2-and-4-cell cluster were subsequently segregated into 2 subclusters based on their developmental stage (Fig. 4B bottom-left panel). After the segregation of *Ahr*^*−/−*^ 8-cell blastomeres from the 2-and-4-cell cluster, no segregation of individual *Ahr*^*−/−*^ 2-cell subcluster was observed (Fig. 4B middle panels). A few *Ahr*^+*/*+^-TCDD 4-cell blastomeres were grouped in the cluster containing 8-cell blastomeres followed by a clear separation of *Ahr*^+*/*+^-TCDD 2-cell and 4-cell subclusters (Fig. 4B right panels). To examine how different the *Ahr*^*−/−*^ and *Ahr*^+*/*+^-TCDD transcriptomes are relative to wild type, we determined the correlation coefficient of 8-cell and 4-cell blastomeres relative to cells belonging to the 2-and-4-cell cluster and the 2-cell subcluster. The correlation coefficient between cells in 8-cell and 2-and-4-cell clusters was significantly increased, i.e., was evidence of higher similarity, in *Ahr*^*−/−*^ relative to *Ahr*^+*/*+^ and the opposite was the case for *Ahr*^+*/*+^-TCDD relative to *Ahr*^+*/*+^ (Fig. 4C). Additionally, both *Ahr*^*−/−*^ and *Ahr*^+*/*+^-TCDD 4-cell blastomeres showed higher correlation than *Ahr*^+*/*+^ cells when compared to the corresponding 2-cell blastomeres (Fig. 4C), indicative of a lower level of cellular heterogeneity in *Ahr*^*−/−*^ and *Ahr*^+*/*+^-TCDD 2-cell and 4-cell blastomeres than in their *Ahr*^+*/*+^counterparts.

To identify possible subpopulation(s) in each of the nine groups, we compared the relative location of each blastomere obtained from hierarchical clustering on the correlation matrices to its relative position on the 3D-tSNE plot. Blastomeres were referred to as either “*Embryonic*” or “*Differentiating*” depending on their spatial location denoted by the arrows indicating the advancement in development on the 3D-tSNE coordinates; blastomeres with mismatching location and position were not included in the subpopulational gene expression analyses. We found that only 2 *Ahr*^+*/*+^ but as many as 11 *Ahr*^*−/−*^ and 7 *Ahr*^+*/*+^-TCDD cells were classified as differentiating blastomeres at the 2-cell stage (Fig. 4D and Supplementary Fig. 1D–F, 1 M). At the 4-cell stage, we identified 16 *Ahr*^+*/*+^ and only 4 *Ahr*^+*/*+^-TCDD and no *Ahr*^*−/−*^ differentiating blastomeres (Fig. 4E and Supplementary Fig. 1G–I, 1 M). At the 8-cell stage, 49 *Ahr*^+*/*+^, 31 *Ahr*^*−/−*^, and 52 *Ahr*^+*/*+^-TCDD blastomeres were considered as differentiating (Fig. 4F and Supplementary Fig. 1 J–L, 1 M). Statistical analysis of the difference in the numbers of differentiating blastomeres in each group showed significant reduction of differentiating cells in both 4-cell *Ahr*^*−/−*^ and *Ahr*^+*/*+^-TCDD embryos and only in 8-cell *Ahr*^*−/−*^ embryos relative to their wild-type counterparts, respectively (Fig. 4G, Supplementary Table 1 and Supplementary Fig. 1 N). Taken together, these results point at the conclusion that the AHR is required in the appearance of early cellular heterogeneity and that its deregulation specifically disrupts the initiation of blastomere differentiation at the 4-cell stage.

### AHR promotes pluripotency downregulation in the 4-cell blastomeres that initiate differentiation

To identify which AHR functions were involved in initiating blastomere differentiation, we used comprehensive transcriptomic analyses via the Ingenuity Pathway Analysis (IPA) platform to explore the biological functions of genes differentially expressed between different groups and subpopulations. We identified, notably those dealing with the downregulation of *Pluripotency Control* and *Metabolism of Inositol Phosphate Compounds* in 4-cell blastomeres undergoing differentiation, suggesting that the AHR has a mechanistic role in the emergence of progenitor blastomeres.

**Fig. 5:**
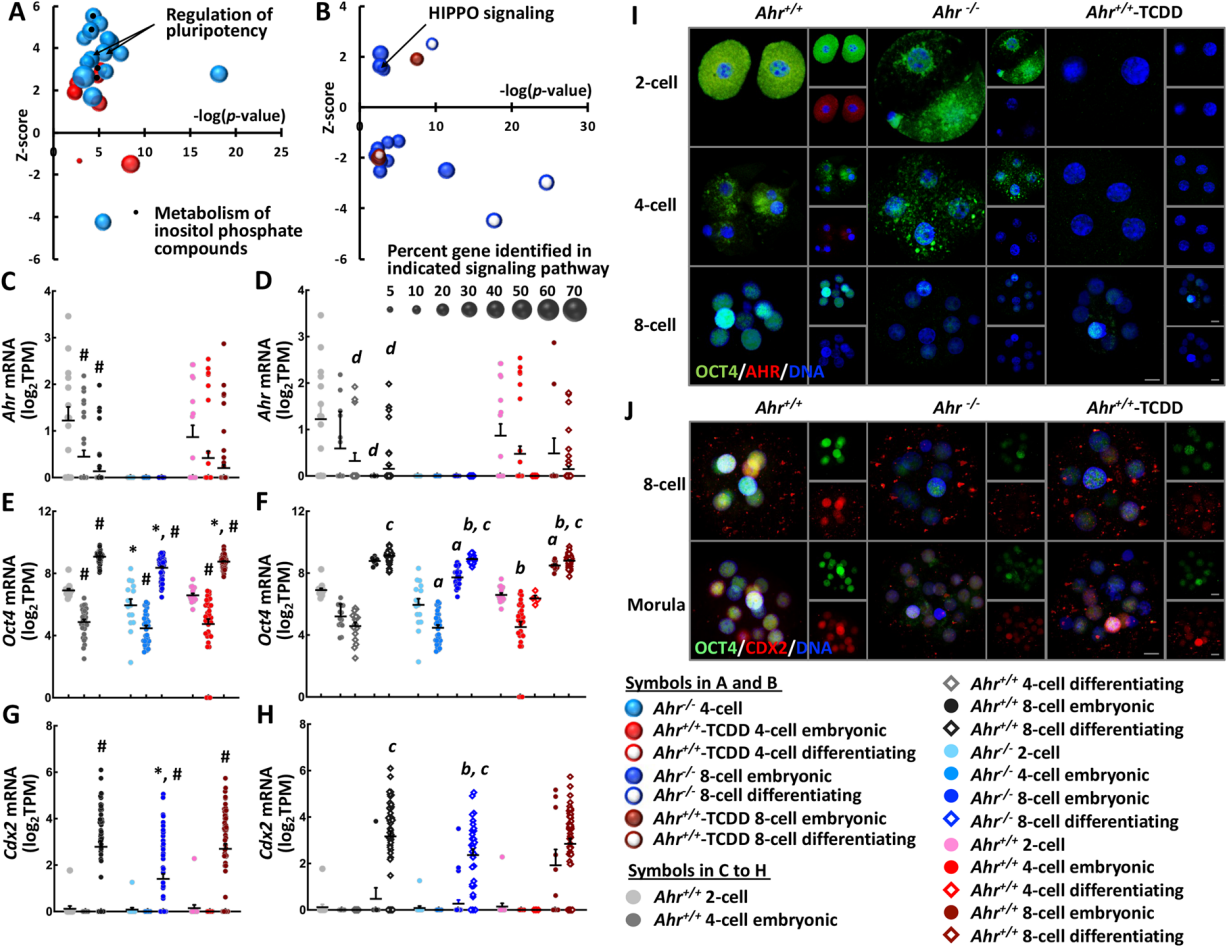
**The AHR Promotes pluripotency downregulation in 4-cell blastomeres that initiate differentiation and sustains the expression and the transcriptional heterogeneity of OCT4 and CDX2 in progenitor blastomeres.** (**A** and **B)** Differentially enriched canonical pathways identified using Ingenuity Pathway Analysis platform in comparisons of *Ahr*^*−/−*^ and *Ahr*^+*/*+^-TCDD 4-cell blastomeres relative to *Ahr*^+*/*+^ 4-cell differentiating blastomere (A) and of 8-cell blastomeres relative to *Ahr*^+*/*+^ 8-cell embryonic blastomeres. Bubble size indicates the percent gene identified in each of the enriched pathways. (**C**–**H)** Dot plots showing mRNA expression levels of *Ahr* (**C** and **D**), *Oct4* (**E** and **F**), and *Cdx2* (**G** and **H**) in the bulk (**C**, **E**, and **G**) and subpopulations (**D**, **F**, and **H**) of *Ahr*^+*/*+^*, Ahr*^*−/−*^, and *Ahr*^+*/*+^-TCDD blastomeres at each of 2-, 4-, and 8-cell stages. *, #, *a*, *b*, *c*, and *d* indicate significant differences resulted from comparisons relative to the bulk of control *Ahr*^+*/*+^ blastomere population (*), to the bulk of blastomere population of the precedent stage and of the same condition (#), to the *Ahr*^+*/*+^ embryonic (*a*) and differentiating (*b*) subpopulations respectively, to the embryonic subpopulation of the same stage and of the same condition (*c*), and to the embryonic subpopulation of the precedent stage of the same condition (*d*), respectively, at *p-*value < 0.05 obtained from ANOVA followed by a posteriori *t*-test. (**I** and **J)** Protein expression of OCT4 and AHR at 2-, 4-, and 8-cell (**I**) stages and of OCT4 and CDX2 at 8-cell and morula (**J**) stages in *Ahr*^+*/*+^*, Ahr*^*−/−*^, and *Ahr*^+*/*+^-TCDD embryos. Images represent flattened z-stakes for the visualization of all blastomeres in the analyzed embryo. Scale bar indicates 10 μm

A higher number of differentially expressed genes and many more canonical pathways were differentially enriched in the *Ahr*^+*/*+^ 2-cell to 4-cell transition than in the 4-cell to 8-cell transition (Supplementary Fig. 2A–F and Supplementary Data 1 and 2), suggestive of a higher degree of transcriptomic changes in 4-cell than in 8-cell blastomeres. Specifically, pathways related to pluripotency control and metabolism of inositol phosphate compounds*,* identified in the *Ahr*^+*/*+^ transition from 2-cell to 4-cell differentiating subpopulations, were not enriched when we compared either *Ahr*^*−/−*^ or *Ahr*^+*/*+^-TCDD 4-cell blastomeres to their 2-cell population (Supplementary Fig. 2G–I and Supplementary Data 2). Therefore, downregulation of pluripotency and metabolism of inositol phosphate may be crucial functions of the AHR in the subpopulation of 4-cell blastomeres undergoing differentiation. Indeed, *Mouse Embryonic Stem Cell Pluripotency*, *Role of NANOG in Mammalian Embryonic Stem Cell Pluripotency*, and *Metabolism of Inositol Phosphate Compounds* were upregulated in the comparison of *Ahr*^*−/−*^ and *Ahr*^+*/*+^-TCDD 4-cell embryonic blastomeres to *Ahr*^+*/*+^ 4-cell differentiating subpopulation (Fig. 5A and Supplementary Data 3).

HIPPO and mTOR signaling pathways were downregulated in the transition of *Ahr*^+*/*+^ 4-cell differentiating subpopulation to the 8-cell blastomeres (Supplementary Fig. 2E, 2F and Supplementary Data 2), suggesting that the initiation of trophoblast differentiation follows promptly the downregulation of pluripotency. Remarkably, signaling pathways involved in pluripotency control and metabolism of inositol phosphate compounds were non-specifically downregulated in the transition of *Ahr*^*−/−*^ 4-cell blastomeres to both 8-cell embryonic and differentiating subpopulations (Supplementary Fig. 2 J, 2 K and Supplementary Data 2). Similar nonspecific downregulation of mTOR signaling was found when comparing *Ahr*^+*/*+^-TCDD 8-cell both subpopulations to 4-cell embryonic blastomeres (Supplementary Fig. 2L–O and Supplementary Data 2). Only *HIPPO signaling* was found upregulated in the comparison of *Ahr*^*−/−*^ 8-cell embryonic blastomeres to *Ahr*^+*/*+^ 8-cell differentiating subpopulation (Fig. 5B and Supplementary Data 3). Collectively, these results suggest that disruption of AHR functions leads to delayed and non-specific regulation of pathways responsible for fate specification of progenitor blastomeres.

### AHR sustains the expression and the transcriptional heterogeneity of OCT4 and CDX2 in progenitor blastomeres

To characterize how AHR regulates the emergence of progenitor blastomeres, we used the data obtained by scRNA-seq to analyze the expression of AHR, OCT4, and CDX2. Consistent with evidence shown by others (Peters and Wiley 1995; Jain et al. 1998; Goolam et al. 2016), we found the highest level of *Ahr* mRNA in the bulk of the *Ahr*^+*/*+^ 2-cell population, followed by successive decreases in the 4-cell and 8-cell embryos (Fig. 5C). No difference was observed in the bulk of the *Ahr*^+*/*+^-TCDD population at all stages analyzed. At the subpopulation level, a significant decrease of *Ahr* mRNA was detected in the transition from the *Ahr*^+*/*+^ 2-cell to the 4-cell differentiating subpopulation; from the *Ahr*^+*/*+^ 4-cell embryonic blastomeres to the 8-cell both embryonic and differentiating subpopulations; and from the *Ahr*^+*/*+^-TCDD 4-cell embryonic blastomeres to the 8-cell differentiating subpopulation (Fig. 5D).

High levels of *Oct4* mRNA were found in the 2-cell blastomere population in all conditions, followed by a decrease at the 4-cell stage and a strong increase in 8-cell blastomeres (Fig. 5E), an expression pattern reflecting the transition of maternal-to-zygotic *Oct4* transcripts at the 4-cell stage (Wu and Scholer 2014). There was a decrease of *Oct4* expression in the bulk of both *Ahr*^*−/−*^ and *Ahr*^+*/*+^-TCDD groups relative to *Ahr*^+*/*+^ at the 8-cell stage and in *Ahr*^*−/−*^ than in *Ahr*^+*/*+^ 2-cell blastomeres. Similarly, we found a significant decrease of *Oct4* expression in both *Ahr*^*−/−*^ and *Ahr*^+*/*+^-TCDD, notably in both embryonic and differentiating blastomeres relative to the corresponding *Ahr*^+*/*+^ 8-cell subpopulations; in all conditions, differentiating blastomeres at 8-cell stage had higher expression levels than their embryonic counterparts (Fig. 5F). Furthermore, the levels of *Oct4* transcription in *Ahr*^*−/−*^ and *Ahr*^+*/*+^-TCDD 4-cell embryonic blastomeres decreased or had a trend to decrease, respectively, relative to the corresponding *Ahr*^+*/*+^ blastomeres. The levels of *Cdx2* mRNA were high in all 8-cell blastomeres analyzed and were significantly decreased or trended to decrease in the bulk of *Ahr*^*−/−*^ and *Ahr*^+*/*+^-TCDD relative to the *Ahr*^+*/*+^ 8-cell population (Fig. 5G). The *Cdx2* mRNA levels were higher in *Ahr*^+*/*+^ and *Ahr*^*−/−*^ 8-cell differentiating blastomeres than in their embryonic counterparts, with the *Ahr*^*−/−*^ 8-cell differentiating blastomeres showing lower expression than the corresponding *Ahr*^+*/*+^ subpopulation (Fig. 5H). Additionally, some *Ahr*^+*/*+^*-*TCDD 8-cell embryonic blastomeres showed unusually high levels of *Cdx2* mRNA causing the indistinguishable difference between embryonic and differentiating subpopulations.

In agreement to the *Ahr* expression at mRNA level, we found high levels and heterogenous AHR expression in *Ahr*^+*/*+^ 2-cell and 4-cell embryos respectively (Fig. 5I). Similar to the observation in *Ahr*^*−/−*^ condition, we did not detect AHR expression in *Ahr*^+*/*+^*-*TCDD embryos, indicating degradation of the AHR after TCDD exposure. As expected, OCT4 and AHR protein expression was stronger in *Ahr*^+*/*+^ 2-cell than in 4-cell embryos (Fig. 5I). In two of the *Ahr*^*−/−*^ 2-cell blastomeres, we found differential OCT4 expression, which was in agreement with the transcriptional heterogeneity observed. We detected AHR^high^-OCT4^high^ and AHR^low^-OCT4^low^ inter-blastomere gene expression heterogeneity in *Ahr*^+*/*+^ 4-cell embryos, and the corresponding expression levels were lower in *Ahr*^*−/−*^ and *Ahr*^+*/*+^-TCDD than in *Ahr*^+*/*+^ 4-cell embryos and no heterogeneity could be detected. At the 8-cell stage, expression of OCT4 was strong and AHR was undetectable in *Ahr*^+*/*+^ embryos while *Ahr*^*−/−*^ and *Ahr*^+*/*+^-TCDD embryos had lowered OCT4 than wild-type. Strong and inter-blastomere heterogeneous CDX2 expression was identified in *Ahr*^+*/*+^ 8-cell embryos, being lower in the *Ahr*^*−/−*^ and *Ahr*^+*/*+^-TCDD counterparts (Fig. 5J and supplementary Fig. 3). A higher degree of OCT4 and CDX2 inter-blastomere heterogeneity was observed in *Ahr*^+*/*+^ morula, which was not the case in both *Ahr*^*−/−*^ and *Ahr*^+*/*+^-TCDD counterparts despite of increased expression levels. These results suggest that the crucial function of the AHR is to maintain the expression level and heterogeneity of OCT4 and subsequently CDX2 in progenitor blastomeres.

### AHR regulates the transcriptional heterogeneity and the differentiation trajectory of progenitor blastomeres

**Fig. 6:**
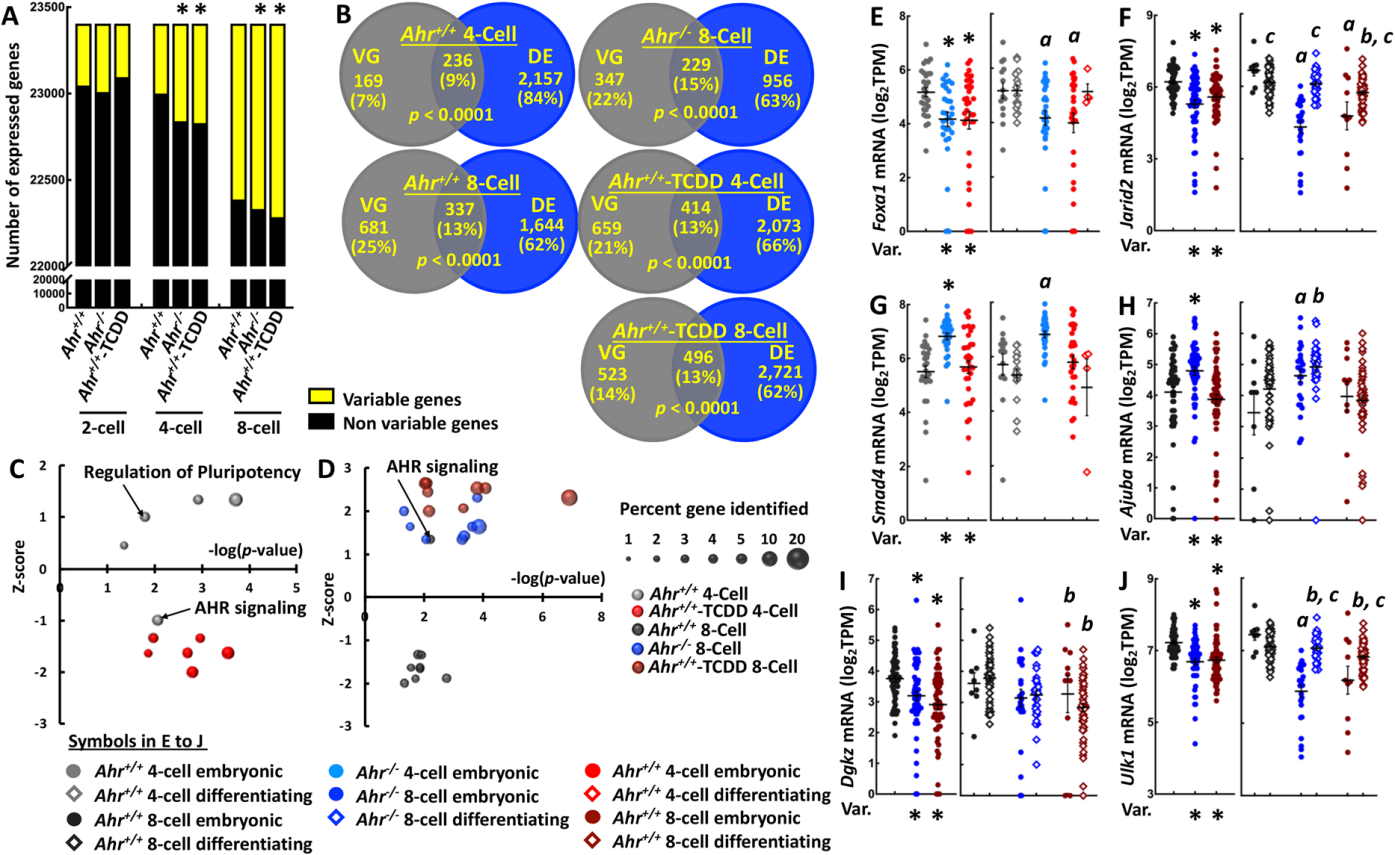
**AHR regulates the transcriptional heterogeneity of progenitor blastomeres.** (**A)** Number of variable genes identified in each of the 9 groups. (**B)** Venn diagrams showing the significant number of genes displaying variable expression levels (VG) and differential expression between paired embryonic and differentiating subpopulations (DE). *p-*values of the significant overlap are indicated. (**C** and **D)** Differentially enriched canonical pathways identified via IPA platform using common genes identified in 4-cell (**C**) and 8-cell (**D**) blastomeres respectively. (**E**–**J)** Comparison between changes of the transcriptional variability in the bulk of blastomere populations and of mRNA expression levels in corresponding subpopulation of selected variable genes. *Foxa1* and *Jarid2* are part of the role of OCT4 in mammalian ES pluripotency; *Smad4* and *Ajuba* belong to HIPPO signaling; and *Dgkz* and *Ulk1* are selected from mTOR signaling. *, ***a***, ***b***, and ***c*** indicate significant differences resulted from comparisons relative to the bulk of control *Ahr*^+*/*+^ blastomere population (*), to the *Ahr*^+*/*+^ embryonic (***a***) and differentiating (***b***) subpopulations respectively, and to the embryonic subpopulation of the same stage and of the same condition (***c***) at *p-*value < 0.05 obtained from ANOVA followed by a posteriori *t*-test. Var. = significant variability relative to control *Ahr*^+*/*+^ condition

We previously showed that fluctuating AHR expression in mouse ES cells regulates the heterogeneous expression of OCT4, leading to an alternative switch between the maintenance and the exit of pluripotency (Ko et al. 2016). As a consequence, AHR may control the variability of gene expression in early embryos and trigger the differentiation of progenitor blastomeres. To address this possibility, we examined genes that showed a higher level of expression variability than expected by chance—the variable genes—in the blastomere population of all 9 groups. We identified no change in *Ahr*^*−/−*^ and *Ahr*^+*/*+^-TCDD 2-cell populations relative to the corresponding *Ahr*^+*/*+^ blastomeres (Fig. 6A, Supplementary Fig. 4A, and Supplementary Data 4). Significantly greater number of variable genes were found in both *Ahr*^*−/−*^ and *Ahr*^+*/*+^-TCDD 4-cell blastomeres relative to the *Ahr*^+*/*+^ counterparts and in *Ahr*^+*/*+^-TCDD 8-cell blastomeres relative to the corresponding *Ahr*^+*/*+^ 8-cell population. This finding suggests that AHR governs the emergence of progenitor blastomeres by regulating the degree of transcriptional variability starting at the 4-cell stage. If it were true that the gene expression variability initiates differentiation of blastomeres, the variable genes would likely share the same identities and biological functions with genes showing differential expression levels between subpopulations. Supporting this assumption, we found a significant number of variable genes that were also differentially expressed between the paired subpopulations in each of the 5 groups in which differentiating blastomeres were identified (Fig. 6B). Comprehensive transcriptomic analysis via IPA revealed that these overlapping genes identified in *Ahr*^+*/*+^ 4-cell blastomeres may be involved in *Mouse ES Cell Pluripotency* and *Xenobiotic Metabolism AHR Signaling Pathway* (Fig. 6C and Supplementary Data 5). *AHR Signaling*, *NRF2-mediated Oxidative Stress Response*, and *Aldosterone Signaling in Epithelial Cells* were identified as the potential function of *Ahr*^+*/*+^, *Ahr*^*−/−*^, and *Ahr*^+*/*+^-TCDD 8-cell overlapping genes respectively (Fig. 6D and Supplementary Data 5). These findings suggest that AHR controls gene expression heterogeneity to differentially regulate pluripotency control among 4-cell blastomeres and promote the segregation of progenitor blastomeres.

In the pathway-related investigation, we found that a significant number of genes involved in the AHR signaling showed heterogeneous expression only among *Ahr*^+*/*+^ 4-cell blastomeres (Supplementary Fig. 4B), suggesting a role of the AHR on regulation of transcriptional heterogeneity at this stage. A few genes of the HIPPO signaling pathway showed variable levels of expression but the corresponding number did not reach significance (Supplementary Fig. 4C). Significant numbers were found for genes involved in the role of OCT4 in pluripotency control in *Ahr*^+*/*+^ and *Ahr*^+*/*+^-TCDD, but not *Ahr*^*−/−*^ 4-cell embryos, followed by a greater number observed at 8-cell stage in all conditions (Supplementary Fig. 4D). Furthermore, significant number of the mTOR signaling genes showing heterogenous expression was only observed among *Ahr*^+*/*+^ 8-cell blastomeres (Supplementary Fig. 4E). Of all scored genes, we found that AHR downregulated expression of *Foxa1* and *Jarid2* in both *Ahr*^*−/−*^ and *Ahr*^+*/*+^-TCDD 4-cell and in 8-cell embryonic subpopulations, respectively, altering the transcriptional variability of OCT4 signaling in the bulk of corresponding blastomere population (Fig. 6E and 6F). Reduced variability of HIPPO signaling in the bulk of *Ahr*^*−/−*^ 4-cell and 8-cell blastomere populations seemed to relate to an upregulated expression of *Smad4* and *Ajuba* in *Ahr*^*−/−*^ 4-cell embryonic and in 8-cell both embryonic and differentiating subpopulations respectively (Fig. 6G and 6H). On the other hand, we found that AHR downregulated expression of *Dgkz* and *Ulk1* in both *Ahr*^*−/−*^ and *Ahr*^+*/*+^-TCDD 8-cell differentiating subpopulations to increase the transcriptional heterogeneity of mTOR signaling in the bulk of *Ahr*^*−/−*^ and *Ahr*^+*/*+^-TCDD 8-cell blastomere populations (Fig. 6I and 6J). Taken together, these results suggest that the AHR promotes segregation of progenitor blastomeres through regulating the transcriptional heterogeneity of OCT4, HIPPO, and mTOR signaling in a stage-dependent manner.

**Fig. 7:**
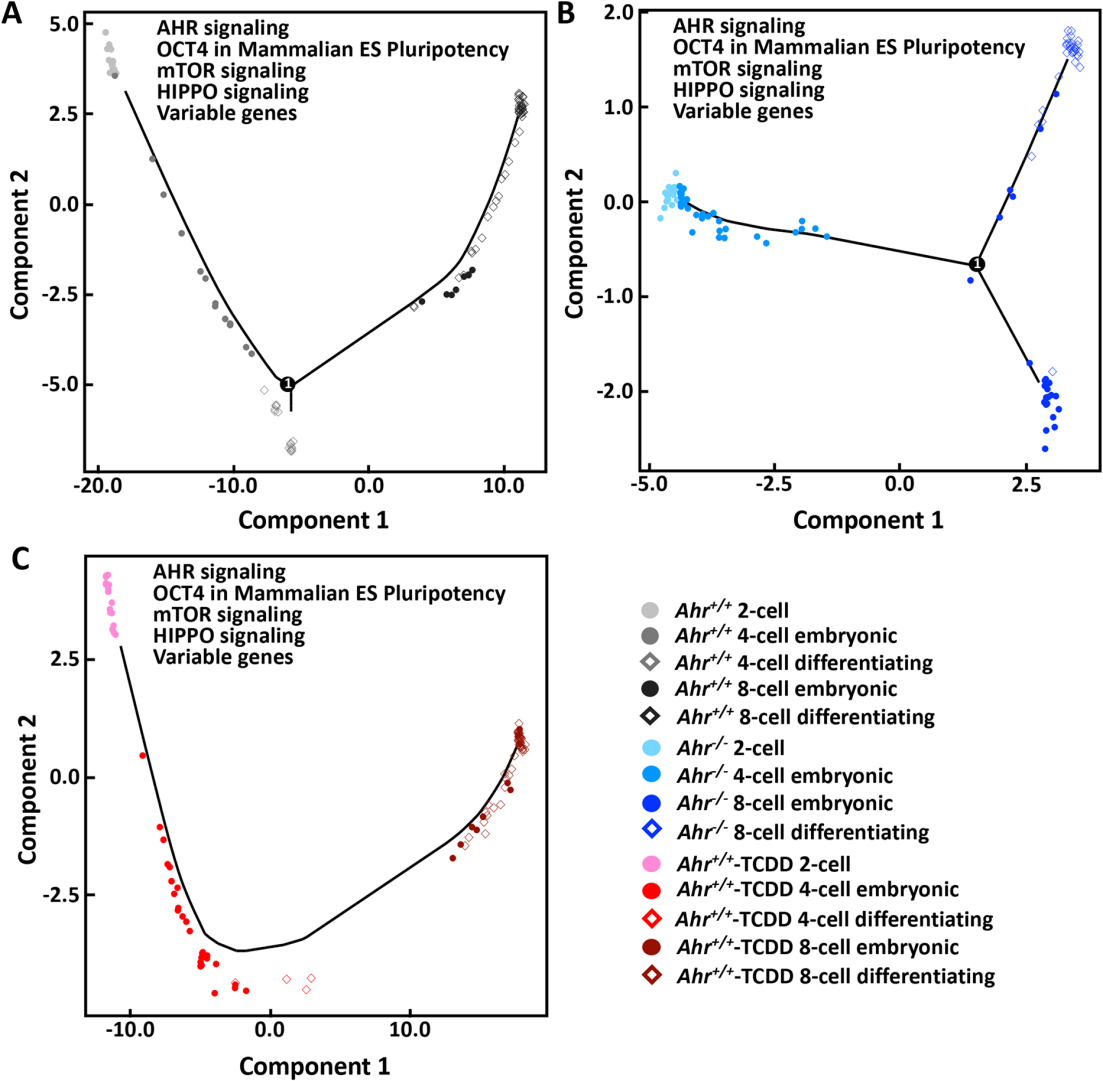
**AHR regulates the differentiation trajectory of progenitor blastomeres.** Projection of trajectories using the collection of genes involved in AHR signaling, role of OCT4 in mammalian ES pluripotency, mTOR signaling, HIPPO signaling, and all variable genes across *Ahr*^+*/*+^ (A) *Ahr*^*−/−*^ (B) and *Ahr*^+*/*+^-TCDD (C) blastomeres. Numbers showed on the branches indicate the changing points within the process of blastomere differentiation that may lead to differential outcome

To explore the consequence in progenitor blastomeres resulting from disruption of AHR functions, we compared the differentiation trajectory of *Ahr*^*−/−*^ and *Ahr*^+*/*+^-TCDD to *Ahr*^+*/*+^. If our finding of the segregation of progenitor blastomeres at the 4-cell stage was true, the structure of the *Ahr*^+*/*+^ trajectory may contain at least one branch indicating the 4-cell differentiating subpopulation. We found that the segregation of *Ahr*^+*/*+^ 4-cell differentiating subpopulation was only projected by genes involved in OCT4 and mTOR signaling and by the collection of genes involved in OCT4, mTOR, and HIPPO pathways, suggesting a role of these pathways and the possible regulatory role of the OCT4 on mTOR and HIPPO signaling in the initiation of progenitor blastomere differentiation (Supplementary Fig. 5A). On the other hand, no stage- and/or subpopulation-wide separation of *Ahr*^+*/*+^ blastomeres could be identified in trajectories projected using neither AHR nor HIPPO signaling genes alone. As expected, we did not observe the branch indicating 4-cell differentiating subpopulation in any of the *Ahr*^*−/−*^ and *Ahr*^+*/*+^-TCDD trajectories projected using genes involved in only one pathway (Supplementary Fig. 5B and 5C). Using the combination of genes of all four pathways and all variable genes, we obtained an *Ahr*^+*/*+^ differentiation trajectory containing the 4-cell differentiation branch, the cluster composed of 8-cell embryonic blastomeres, and the cluster including the majority of 8-cell differentiating blastomeres (Fig. 7A). Consistently, the *Ahr*^*−/−*^ 4-cell blastomeres were not separated from their 2-cell counterparts, and the earliest differentiation of *Ahr*^*−/−*^ embryonic cells was shown at the 8-cell stage (Fig. 7B). On the other hand, we identified neither 4-cell branch nor 8-cell segregation in the *Ahr*^+*/*+^-TCDD differentiation trajectory (Fig. 7C). These results indicate that the AHR regulates the transcriptional heterogeneity and pathways playing essential roles in preimplantation development to direct the differentiation of progenitor blastomeres.

## Discussion

**Fig. 8:**
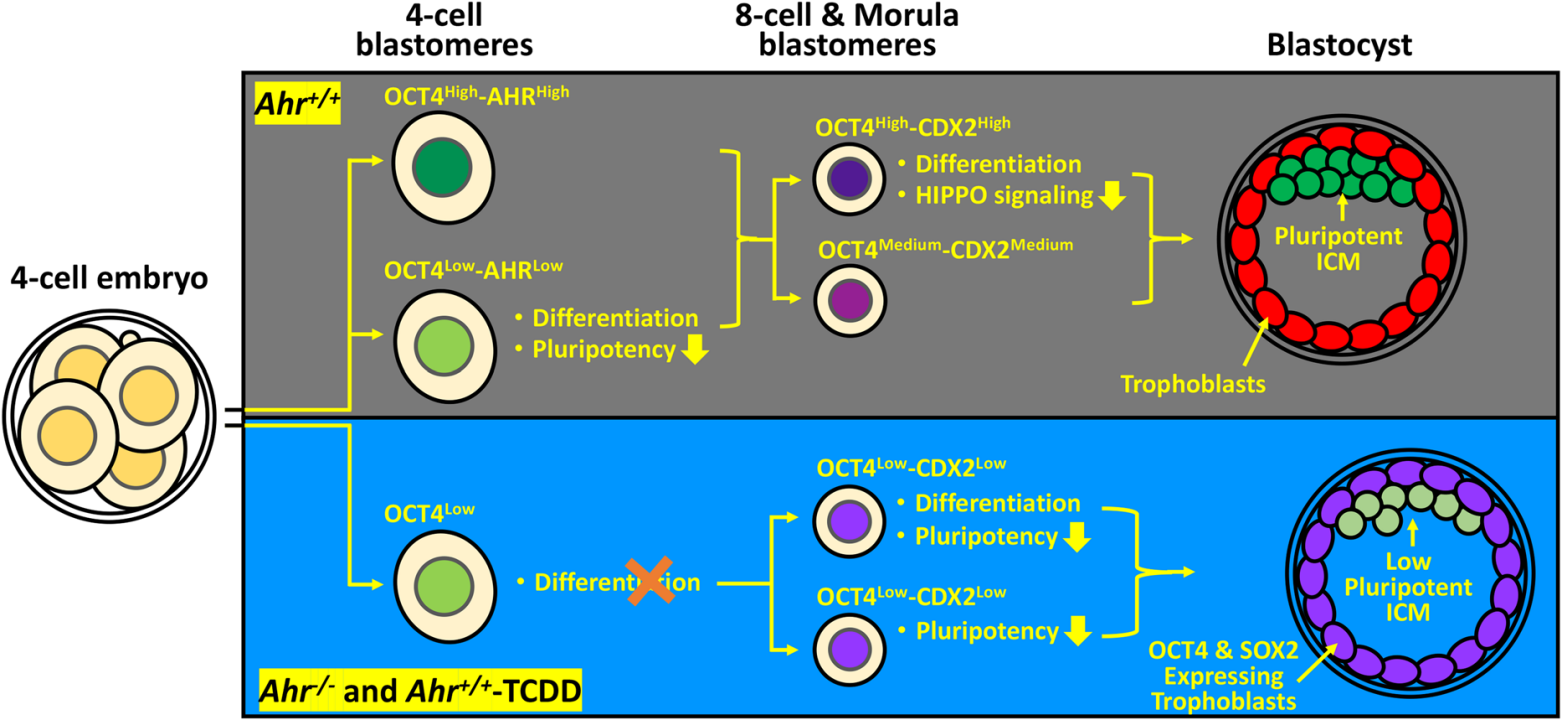
**AHR directs the differentiation of progenitor blastomeres.** Schematic illustration of differentiation of *Ahr*^+*/*+^, *Ahr*^*−/−*^, and *Ahr*^+*/*+^-TCDD progenitor blastomeres. In the wild-type 4-cell embryos where the AHR is heterogeneously expressed, OCT4^Low^-AHR^Low^ blastomeres undergo differentiation through downregulation of the pluripotency. Expression of both OCT4 and CDX2 subsequently upregulates in 8-cell embryos; and downregulation of the HIPPO signaling causes the mosaic of OCT4^High^-CDX2^High^ and OCT4^Medium^-CDX2^Medium^ heterogenous expression pattern, initiating differentiation of OCT4^High^-CDX2^High^ blastomeres. In contrast, disruption of AHR functions reduces the expression and suppresses the transcriptional heterogeneity of both OCT4 and CDX2, resulting in absence of differentiation and non-specific downregulation of pluripotency in *Ahr*^*−/−*^ and *Ahr*^+*/*+^-TCDD 4-cell and 8-cell embryos respectively. Different to the pluripotent ICM and the differentiated trophoblasts in the wild type *Ahr*^+*/*+^ blastocysts, aberrant differentiation of *Ahr*^*−/−*^ and *Ahr*.^+*/*+^-TCDD progenitor blastomeres leads to formation of blastocysts with low pluripotent state ICM and trophoblasts expressing pluripotency factors OCT4 and SOX2

In this article, we show that the AHR, an environmental sensor and transcription factor that regulates the pluripotency in ES cells, directs the differentiation of progenitor blastomeres that determine the pluripotent state of ICM cells in blastocysts. As illustrated in the summary Fig. 8, AHR functions to promote the downregulation of pluripotency in 4-cell blastomeres that initiate differentiation. Disruption of this function by *Ahr* ablation, or its xenobiotic activation, leads to repression of inter-blastomere transcriptional heterogeneity of OCT4 in 4-cell embryos and of CDX2 in 8-cell and morula stage embryos. As a consequence, *Ahr*^*−/−*^ and *Ahr*^+*/*+^-TCDD blastomeres follow derailed differentiation trajectories and form impaired blastocysts with low pluripotent state in the ICM and abnormal expression of pluripotency factors in trophoblasts, which may cause a poor reproductive outcome due to defective embryogenesis.

Previously, we have shown that AHR regulates the maintenance of pluripotency and interferes with the differentiation outcome of mouse ES cells (Ko et al. 2016). Unlike the association between AHR expression and the differentiated state of pluripotent stem cells shown by in vitro studies (Ko et al. 2014; Morales-Hernandez et al. 2016), expression of the AHR remains higher in the embryonic subpopulation than in the differentiating one, leading to AHR^high^-OCT4^high^ and AHR^low^-OCT4^low^ heterogeneity in 4-cell embryos. By sustaining heterogenous expression of OCT4, the AHR may upregulate CDX2 expression and direct the differentiation of 8-cell OCT4^high^-CDX2^high^ blastomeres. The AHR may also prevent the downregulation of pluripotency in embryonic blastomeres by maintaining a homogenous expression of *Foxa1* and *Jarid2* that have been shown to be involved in the sustenance of accessible promoters and WNT signaling in preimplantation embryos (Landeira et al. 2015; Jung et al. 2019). As a consequence, lack of AHR in *Ahr*^*−/−*^ and *Ahr*^+*/*+^-TCDD (plausibly due to the proteasomal degradation of the AHR protein after activation by TCDD) embryos results in repression of OCT4 and CDX2 in all blastomeres, causing lack of 4-cell and derail of 8-cell blastomere differentiation, and reduction of ICM pluripotency. Alongside the OCT4-mediated pluripotency control, the AHR differentially regulates HIPPO and mTOR signaling to establish a distinct regulatory profile between embryonic and differentiating blastomeres. HIPPO and mTOR signaling pathways have been shown to control the self-renewal of stem-like cells and the reprogramming to pluripotency (Bora-Singhal et al. 2015; Lee et al. 2020). Yet, the interaction between these two signalings was considered a response to stress stimuli arising from energy metabolism, hypoxia, reactive oxygen species, and mechanical forces in stem cells (Zeybek et al. 2021). Accordingly, upregulated expression and variability of genes involved in HIPPO signaling such as *Smad4* and *Ajuba* in *Ahr*^*−/−*^ blastomeres suggest that the coordinating function of the AHR is required to support the specification of trophoblast differentiation in corresponding precursor cells (Anani et al. 2014; Hirate et al. 2015). Similarly, the AHR may also sustain mTOR signaling-mediated metabolism and embryonic physiology to ensure the segregation of differentiating blastomeres at the 8-cell stage (O'Neill 2008).

Concurrently, the AHR may downregulate the metabolism of inositol phosphate compounds to initiate blastomere differentiation. The derivatives of inositol phosphate metabolism are involved in intracellular calcium signaling and mTOR signaling essential to the maintenance of preimplantation embryonic physiology (O'Neill 2008; Armant 2015). Furthermore, inositol phosphate compounds may be one of the critical energy sources of preimplantation embryos due to the low mitochondrial activities in embryos at these stages and the hypoxic environment in the female reproductive tract (Kumar et al. 2018). Therefore, AHR may regulate the energy supply requirements that are closely related to the outcome of epigenetic reprogramming in blastomeres undergoing fate commitment (Chason et al. 2011; Harvey 2019). Induction of compensative metabolism in developing embryos under stress has been suggested to cause the later-onset pathological conditions, notably metabolic and cardiovascular diseases (Watkins et al. 2008; Chason et al. 2011; Fleming et al. 2017). In line with this hypothesis, AHR is known to mediate metabolism-related syndromes including obesity, insulin resistance, and liver fibrosis, and cardiovascular conditions such as cardiac hypertrophy (Kerley-Hamilton et al. 2012; Carreira et al. 2015; Brulport et al. 2017; Duval et al. 2017; Girer et al. 2020; Bock 2021). In conclusion, results of the present study suggest that a functional AHR is necessary to maintain the pluripotency in ICM and the proper differentiation of preimplantation embryos. Due to the importance of the preimplantation embryonic cells to the subsequent developmental program, disruption of preimplantation AHR functions may contribute to long-lasting pathological conditions to increase disease susceptibility.

## Materials and methods

### Preimplantation exposure, embryo collection, and in vitro embryo culture

#### Mice

All experiments were conducted using the highest standards of human care in accordance with the NIH Guide for the Care and Use of Laboratory Animals and were approved by the University of Cincinnati Institutional Animal Care and Use Committee. Mice were housed in a pathogen-free animal facility under a standard 12-h light/12-h dark cycle with ad libitum water and chow. Eight-week-old mice were used for all experiments, and all *Ahr*^*−/−*^ mice used for experiments were the F1 generation obtained from the breeding between *Ahr*^+/-^ female and *Ahr*^*−/−*^ male.

#### Superovulation and dioxin exposure

Female mice were superovulated by intraperitoneal injection of 7.5-I.U. pregnant mare serum gonadotropin (PMSG, Calbiochem, Burlington, MA), followed by 7.5-I.U. human urine chorionic gonadotropin (hCG, Sigma, Saint Louis, MO) before overnight mating with individually housed stud males of the same genotype. Preimplantation exposure to 1 μg/kg TCDD (Cambridge Isotope Laboratory, Tewkesbury, MA) and equivalent volume of corn oil for vehicle control were performed via oral gavage at gestational days E-0.5 and E2.5 (Supplementary Fig. 6).

#### Litter size and neonatal body weight determination

At E19.5, the numbers of newborns and the numbers of implantation sites in uteri of the same dam were scored no later than 10 h after delivery. The numbers of missing and dead pups were determined by subtracting the number of newborns from that of implantation sites. The body weight of each of the postnatal day 1 pups were scaled and recorded individually.

#### Embryo collection

Details of experimental scheme regarding embryo collection can be found in Supplementary Fig. 6. Two-cell, 4-cell, 8-cell, and blastocyst stage embryos were collected from superovulated naïve *Ahr*^+*/*+^and *Ahr*^*−/−*^ females that were mated at E-0.5 with males of the corresponding genotype and exposed to control oil vehicle or TCDD at 46-, 55-, 70-, and 97-h post-hCG time into M2 media (Millipore, Burlington, MA), respectively. Litter sizes and morphology of 2-cell embryos and blastocysts were observed using a binocular stereomicroscope (AmScope, Irvine, CA).

#### *In vitro* embryo culture

Naïve *Ahr*^+*/*+^ and *Ahr*^*−/−*^ zygotes were recovered from plugged *Ahr*^+*/*+^ and *Ahr*^*−/−*^ females, respectively, into M2 medium at 21-h post-hCG time (Supplementary Fig. 6). After removing granulosa cells by a short incubation in M2 medium containing hyaluronidase (Millipore) at 37 °C, embryos were cultured in KSOM media (Millipore and CytoSpring, Mountain View, CA) in a humidified incubator at 37 °C 5% CO_2_ for 5 days. In vitro exposure to 1-nM TCDD or to 10-μM AHR antagonist CH223191 (Cambridge Chemical Technologies, Cambridge, MA) was initiated before both male and female pronuclei could be clearly visualized, using DMSO dissolved in KSOM at ≤ 0.05% of the final volume as vehicle control. More than 4 replicates were performed for each experimental condition; the numbers of embryos that had reached the 2-cell stage and of embryos that formed blastocyst were scored. Efficiency of blastocyst formation was calculated as percent embryo relative to the number of embryos observed at the 2-cell stage.

### Immunofluorescence and scoring of labeled embryonic cells

#### Immunofluorescence

Embryos were fixed in 2% paraformaldehyde (Sigma) for 10 min at room temperature and washed in 3 µg/ml polyvinylpyrrolidone dissolved in 1X PBS (Gibco, Waltham, MA, hereafter referred to as PVP-PBS). Primary antibodies against CDX2 (MA5-14,494, Thermo Fisher Scientific, Waltham, MA), OCT4 (AF-1759, R&D Systems, Minneapolis, MN), SOX2 (AF2018, R&D Systems), and NANOG (PA5-47,376, Invitrogen-Thermo Fisher Scientific), the conjugated antibody against AHR (12–5925-82, eBiosciences-Thermo Fisher Scientific) as well as Alexa-488 and Alexa-594 conjugated secondary antibodies (Invitrogen-Thermo Fisher Scientific) were used. After permeabilized in 0.25% Triton X-100 (Sigma) prepared in PVP-PBS at room temperature for 10 min, embryos were incubated with blocking buffer containing 5% donkey serum (D9663, Sigma), 2% BSA (4,022,052, Bio-World, Irving, TX), and 0.01% Tween-20 (Sigma) in PVP-PBS at room temperature for 1 h before applying primary antibody at 4 °C overnight. The next day, embryos were washed in series of PVP-PBS drops before applying secondary antibodies diluted in blocking buffer at room temperature for an hour. After washes, embryos were mounted in cavity slides (71,883–79, Globe Scientific Inc, Mahwah, NJ) with 45 μl of fluorescent mounting medium containing DAPI (H1200, Vector Laboratories, Burlingame, CA) and settled down in the dark overnight before visualization using confocal microscopy (LSM700 Carl Zeiss Microscopy, Dublin, CA).

#### Scoring of fluorescent embryonic cell

The number of OCT4- (green channel), SOX2- (green channel), NANOG- (green channel), and CDX2-expressing (red channel) embryonic cells were scored by manual counting throughout the z-stacks of each of the immunostained blastocysts; one example using OCT4 and CDX2-immunostained blastocyst was illustrated in Supplementary Fig. 7. The number of DAPI-stained embryonic cells was recorded as the reference number of embryonic cells in each of the blastocysts analyzed. For each of the immunostained blastocysts, the numbers of OCT4/SOX2/NANOG-CDX2 double-stained, OCT4/SOX2/NANOG-expressing, and CDX2-expressing cells were scored individually. The number of OCT4/SOX2/NANOG-expressing embryonic cells was further subdivided into either ICM or trophoblast fractions based on the relative location of each cell within blastocysts.

### Isolation of single blastomeres and single-cell RNA sequencing

#### Isolation of single blastomeres

Embryos were incubated in Tyrode’s solution (Sigma) at room temperature to remove the *zona pellucida* before manual isolation of healthy single blastomeres using Femtotip II (Eppendorf, Hauppauge, NY) and carefully pipetted into cell lysis buffer containing RNase inhibitor.

#### Single-cell RNA-sequencing

Messenger RNAs obtained from lysed single blastomeres were converted into full-length cDNA by directly following the manufacture’s protocol of SMART-seq v4 ultra-low input RNA kit with some modifications (Takara Bio, Mountain View, CA). A 1.5-μl volume of 1X PBS containing 0.4% of BSA and one single blastomere was transferred into PCR tubes containing the lysis buffer and 10X RNase inhibitor provided by the kit. cDNA of each single blastomere was prepared by first-strand-cDNA conversion from polyA-RNA followed by 18 cycles of full-length amplification. After quality control using a Bioanalyzer High Sensitivity DNA kit (Agilent, Santa Clara, CA) and concentration measurement using Qubit dsDNA HS assay (Thermo Fisher Scientific), 150 pg of amplified cDNA were subjected to library preparation using a Nextera XT DNA library preparation kit (Illumina, San Diego, CA). After quantification using NEBNext Library Quant kit (NEB, Ipswich, MA), libraries were pooled and sequenced on the next-generation sequencer HiSeq 1000 (Illumina) under setting of 2 × 101-bp pair-ends base pairs.

### Data Analysis of scRNA-seq

#### Data processing and quality control

Three hundred thirty-six single blastomeres were isolated and submitted to scRNA-seq, and 322 single-cell transcriptomes were generated. Fastq files with sequence reads were generated with bcl2fastq version 2.18 (Illumina). RNA-seq pair-end fastq were validated with fast-QC and summarized with multi-QC, and low quality cells, outliers were removed from further consideration in appropriate stages of analyses. The transcriptomes of 322 single blastomeres were sequenced at an averaged depth of ~ 2,600,000 reads/cell, of which an average of 86.52% was mapped to single or multiple gene loci. Count and transcript per million (TPM) data for reads were generated based on output of STAR aligner.

#### Cluster analysis

Three-dimensional t-distributed stochastic neighbor embedding (3D-tSNE) visualization of the all variable genes across all cells were generated using the tSNE plotly R packages. Hierarchical clustering of cells based on log2 transformed TPM for all genes with expression level ≥ 1 was performed with the Morpheus platform (https://software.broadinstitute.org/morpheus) using 1-Pearson’s correlation as the distance measurement. The cluster analysis of cell–cell correlation matrix was performed by first calculating all vectors of pairwise correlations between a single cell and all other cells followed by hierarchical clustering of rows and columns of the resulting correlation matrix using 1-Pearson’s correlation as the distance measurement.

#### Comprehensive transcriptomic analysis

Lists of differentially expressed genes between groups and subpopulations were submitted to comprehensive transcriptomic analysis via Ingenuity Pathway Analysis (IPA, Qiagen, Germantown, MD). Canonical pathways that were commonly identified in analyses of comparisons using six statistical cutoff levels were considered to be differentially enriched.

#### Identification of variable genes

The expression variability of each gene was estimated by following the parametric equation CV^2^ = a_1_/μ + a_0_, where the square of coefficient of variation CV^2^ was a function of the mean after normalization with the count number μ. Genes with an adjusted *p*-value (FDR) < 0.1 were considered as significantly variable.

#### Trajectory analysis

Projection of blastomere differentiation trajectories was performed with count data of selected genes using Monocle-2 algorithm. Analytic steps included the estimation of size factors, the estimation of dispersion and normalization, the selection of subspace for analysis, the reduction of dimension, and the visualization of trajectories.

### Statistical analysis

Statistical analyses were performed via R and/or Prism. Details of all statistical analyses carried out throughout this study are provided in the file entitled Statistical Information; a *p-*value < 0.05 was considered significant.

## Supplementary Information

Below is the link to the electronic supplementary material.

Supplementary file1 (DOCX 53953 KB)

Supplementary file2 (DOCX 35.1 KB)

Supplementary file3 (xlsx 14.9 KB)

Supplementary file4 (xlsx 21.8 KB)

Supplementary file5 (xlsx 13.9 KB)

Supplementary file6 (xlsx 927 KB)

Supplementary file7 (xlsx 17.9 KB)

## Data Availability

Supporting data will be available from the corresponding author upon reasonable request.
