# Supplementary material for: The aryl hydrocarbon receptor directs the differentiation of murine progenitor blastomeres
Source: Cell Biol Toxicol. 2022 Aug 27;39(4):1657–76. doi: 10.1007/s10565-022-09755-9 (PMC10425484; doi:10.1007/s10565-022-09755-9)
Supplement: Supplementary file 1 — Supplementary file1 (DOCX 53953 KB) [file 10565_2022_9755_MOESM1_ESM.docx]

Supplemental Information for

**The Aryl Hydrocarbon Receptor Directs the Differentiation of Murine Progenitor Blastomeres**

**Ko *et al.***

**Table of contents**

Table of contents. **……………………………………………………………………………. 01**

Supplemental Figure 1. AHR Directs the Segregation of the 4-cell Blastomeres. **…………. 02**

Supplemental Figure 2. Differentially Enriched Canonical Pathways Identified in *Ahr^+/+^* and *Ahr^-/-^*, embryonic development. **………………………………………………………………... 16**

Supplemental Figure 3. AHR Regulates the Expression Levels and Interblastomere Heterogeneity of OCT4 and CDX2. **………………………………………………………………………. 31**

Supplemental Figure 4. AHR Regulates the Transcriptional Heterogeneity. **……………… 37**

Supplemental Figure 5. AHR Regulates the Differentiation Trajectory of Progenitor Blastomeres. **……………………………………………………………………………… 42**

Supplemental Figure 6. Experimental Scheme for Superovulation and Embryo Collection. **……………………………………………………………………………….... 45**

Supplemental Figure 7. Example of Scoring for Pluripotency Factor- and CDX2-expressing Embryonic Cells within Blastocyst. **………………………………………………………… 46**

**Additional Files**

Supplemental Data 1

Supplemental Data 2

Supplemental Data 3

Supplemental Data 4

Supplemental Data 5

Supplemental Table 1

1

**Supplemental Figure 1. AHR Directs the Segregation of 4-cell Blastomeres.**

**
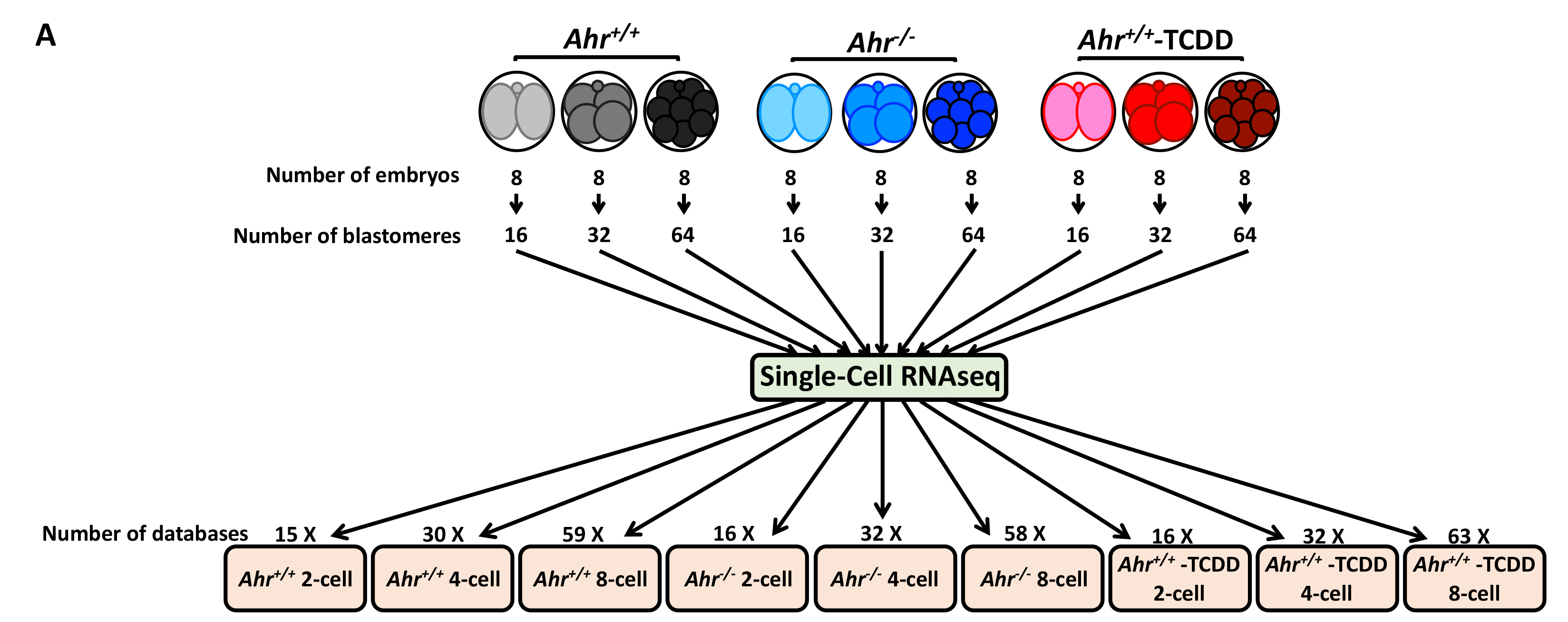
**

**Supplemental Fig. 1A.** Experimental scheme of Single-Cell RNA-sequencing (scRNA-seq).

**
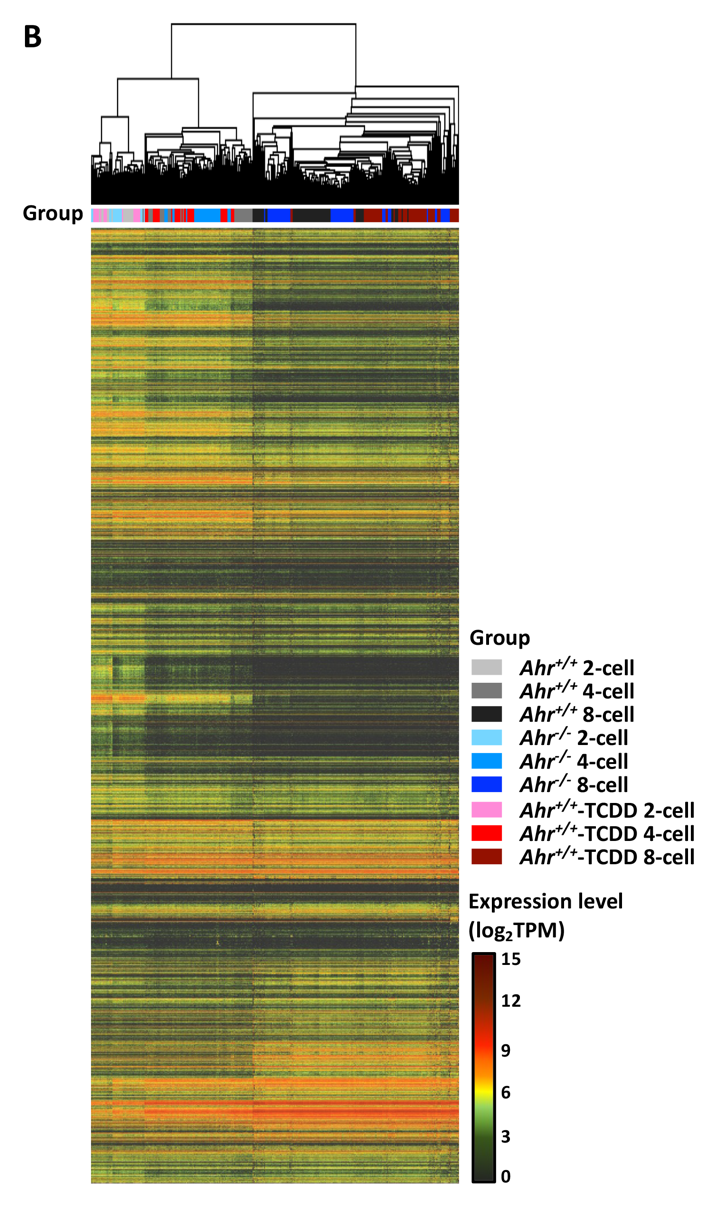
**

**Supplemental Fig. 1B.** Heatmap composed of genes with expression level *≥* 1 transcript per million (TPM) across all 322 single blastomeres showed 2 groups of blastomeres classified by unsupervised hierarchical clustering.

**
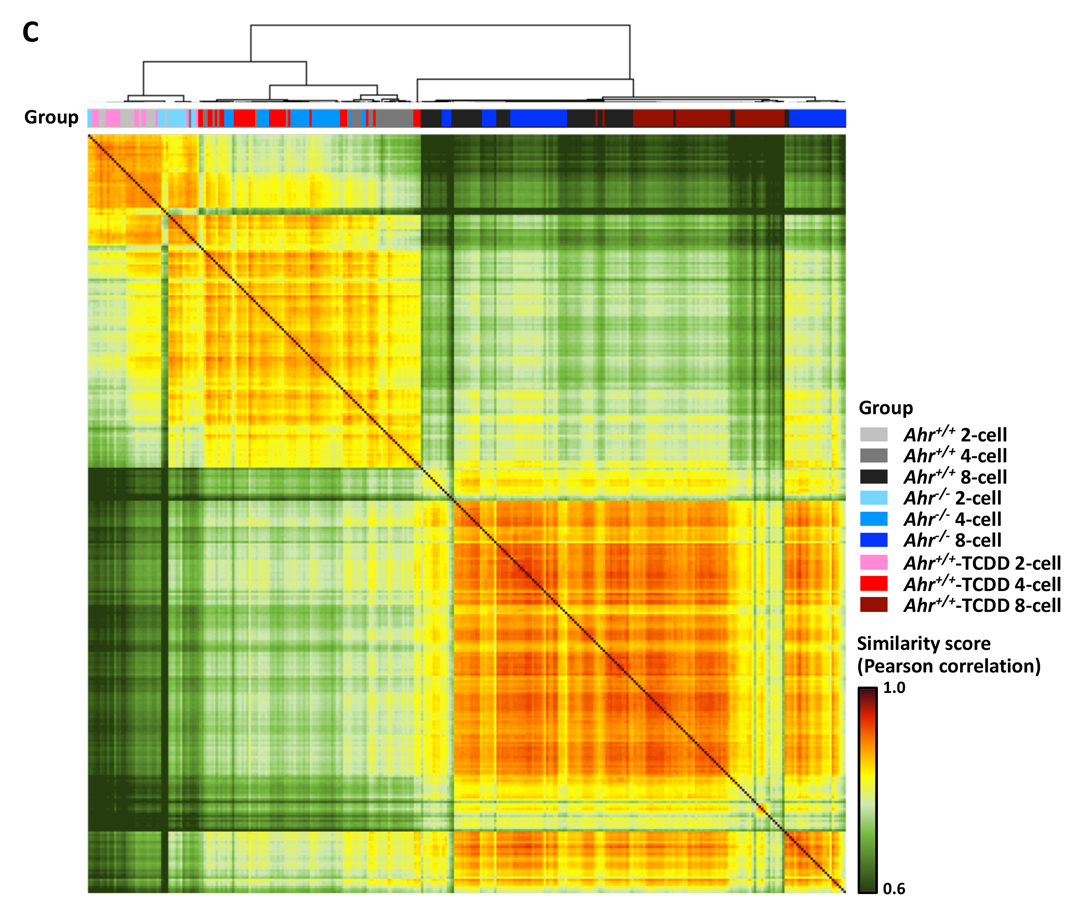
**

**Supplemental Fig. 1C.** Cell-cell Pearson’s correlation matrix using all expressed genes across all 322 single blastomeres followed by unsupervised hierarchical clustering. Top and right-side color bars indicate group and correlation coefficient of each blastomere.

**Supplemental Fig. 1D.** Identification of differentiating blastomeres in the bulk of *Ahr^+/+^* 2-cell blastomere population**
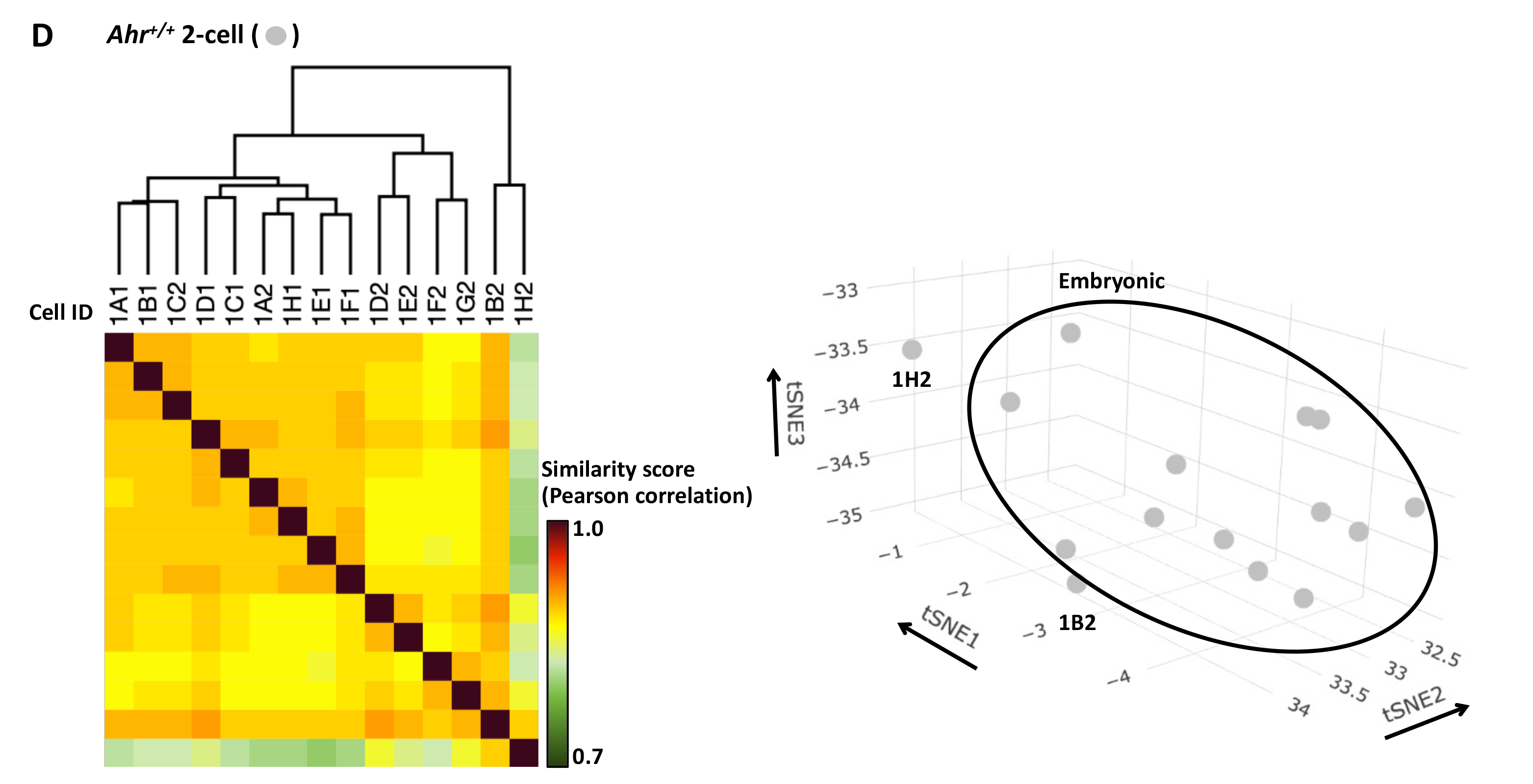
.**

**Supplemental Fig. 1E.** Identification of differentiating blastomeres in the bulk of *Ahr^-/-^* 2-cell blastomere population **
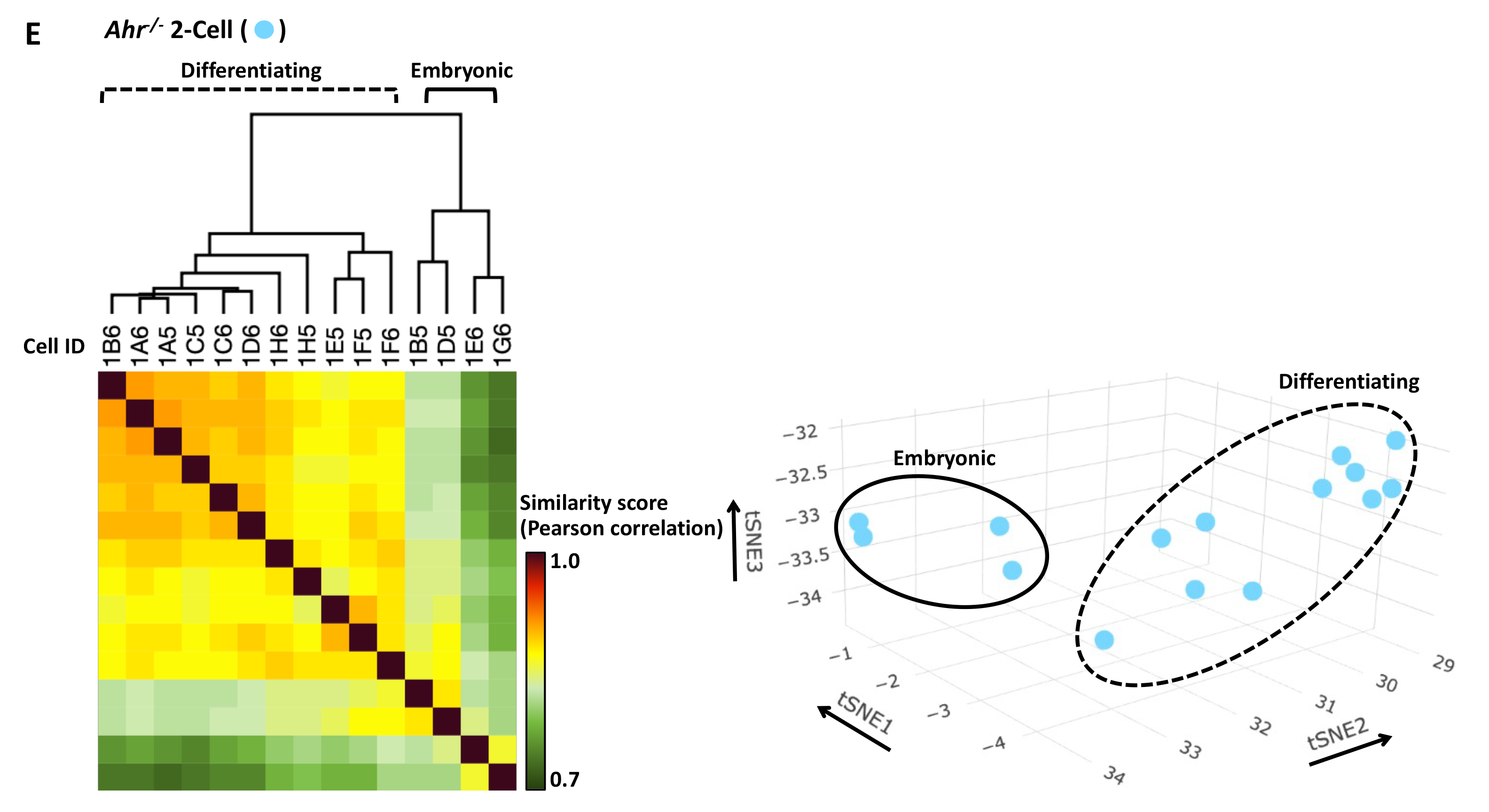
**

**Supplemental Fig. 1F.** Identification of differentiating blastomeres in the bulk of *Ahr^+/+^*-TCDD 2-cell blastomere population**.
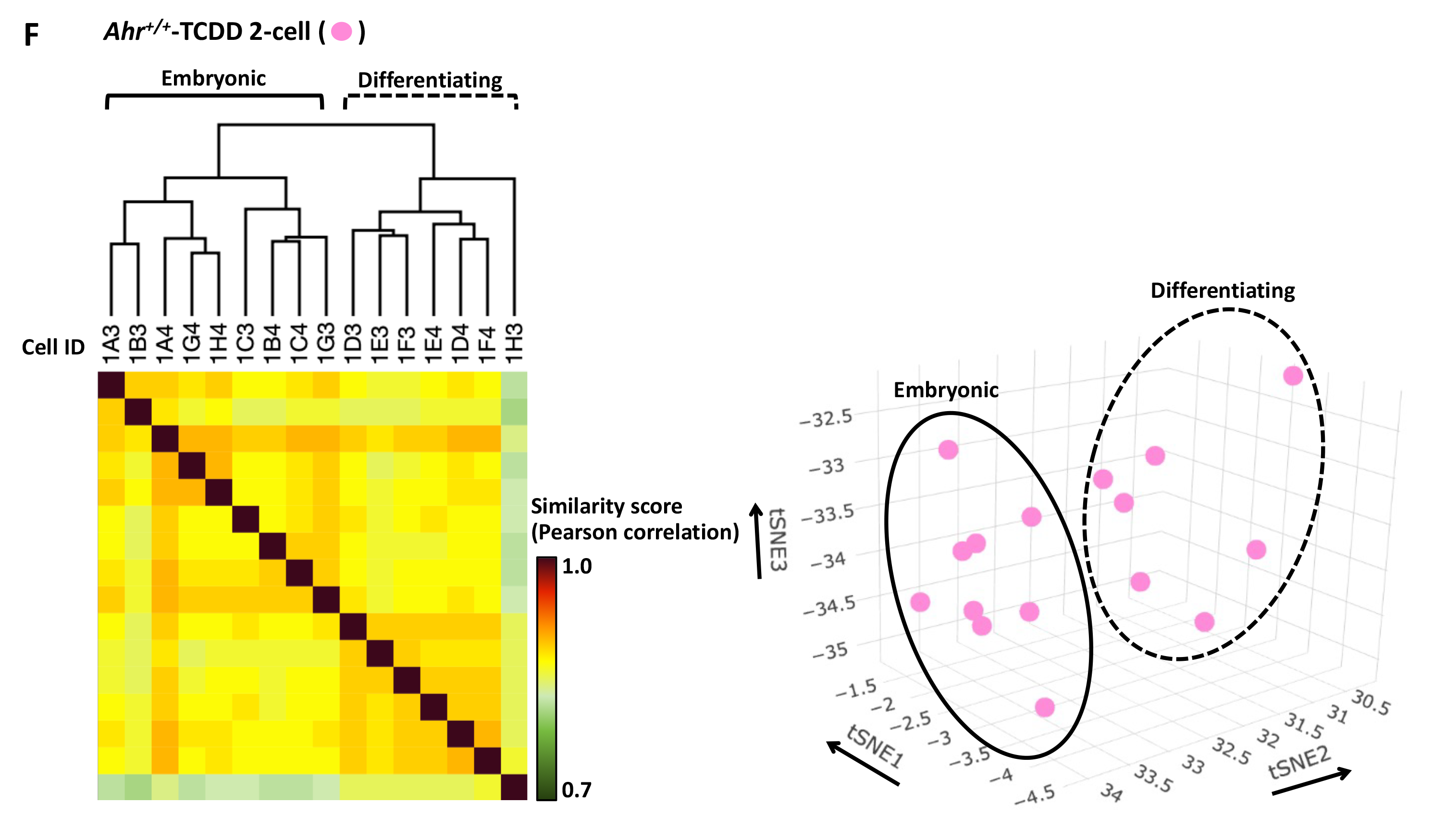
**

**
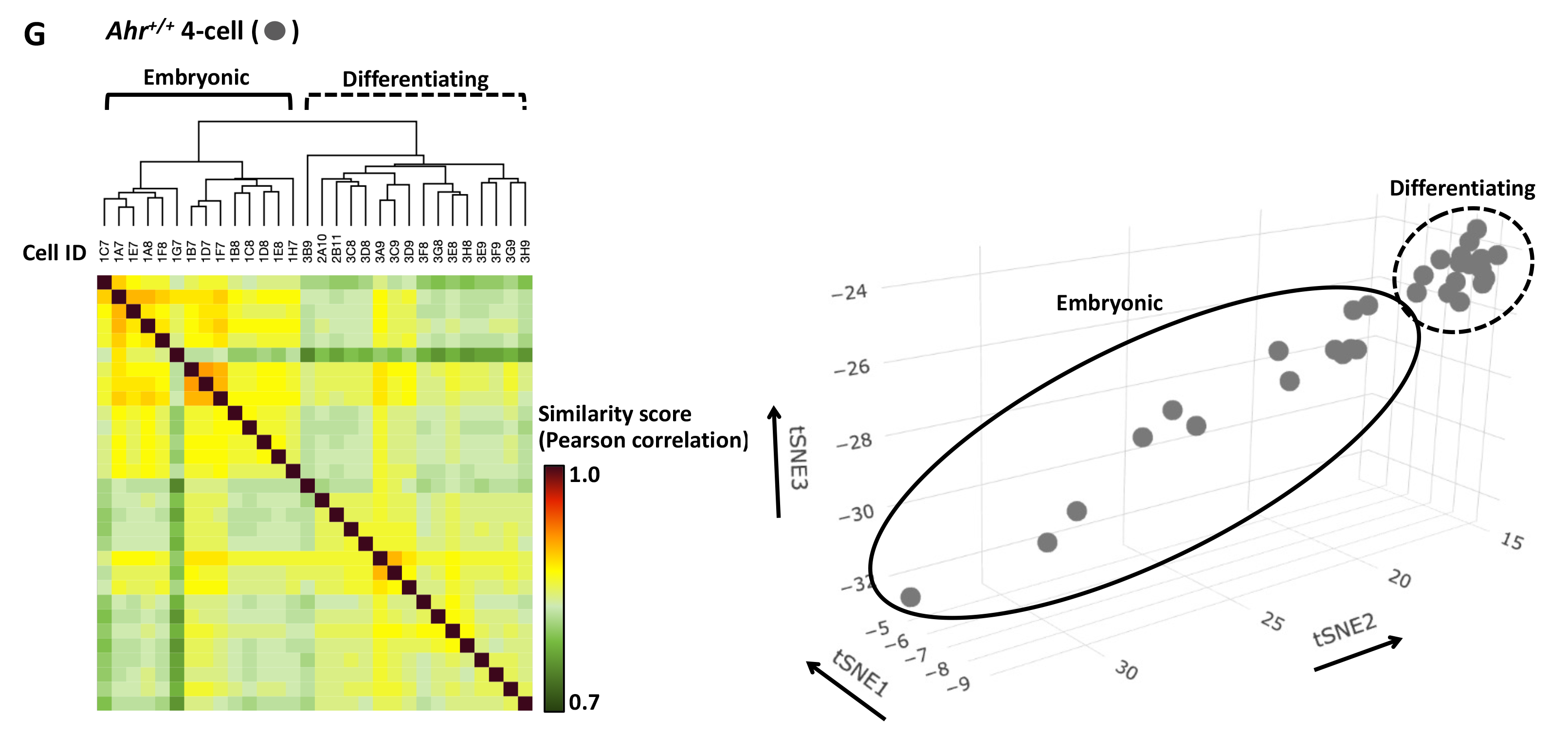
**

**Supplemental Fig. 1G.** Identification of differentiating blastomeres in the bulk of *Ahr^+/+^* 4-cell blastomere population.

**
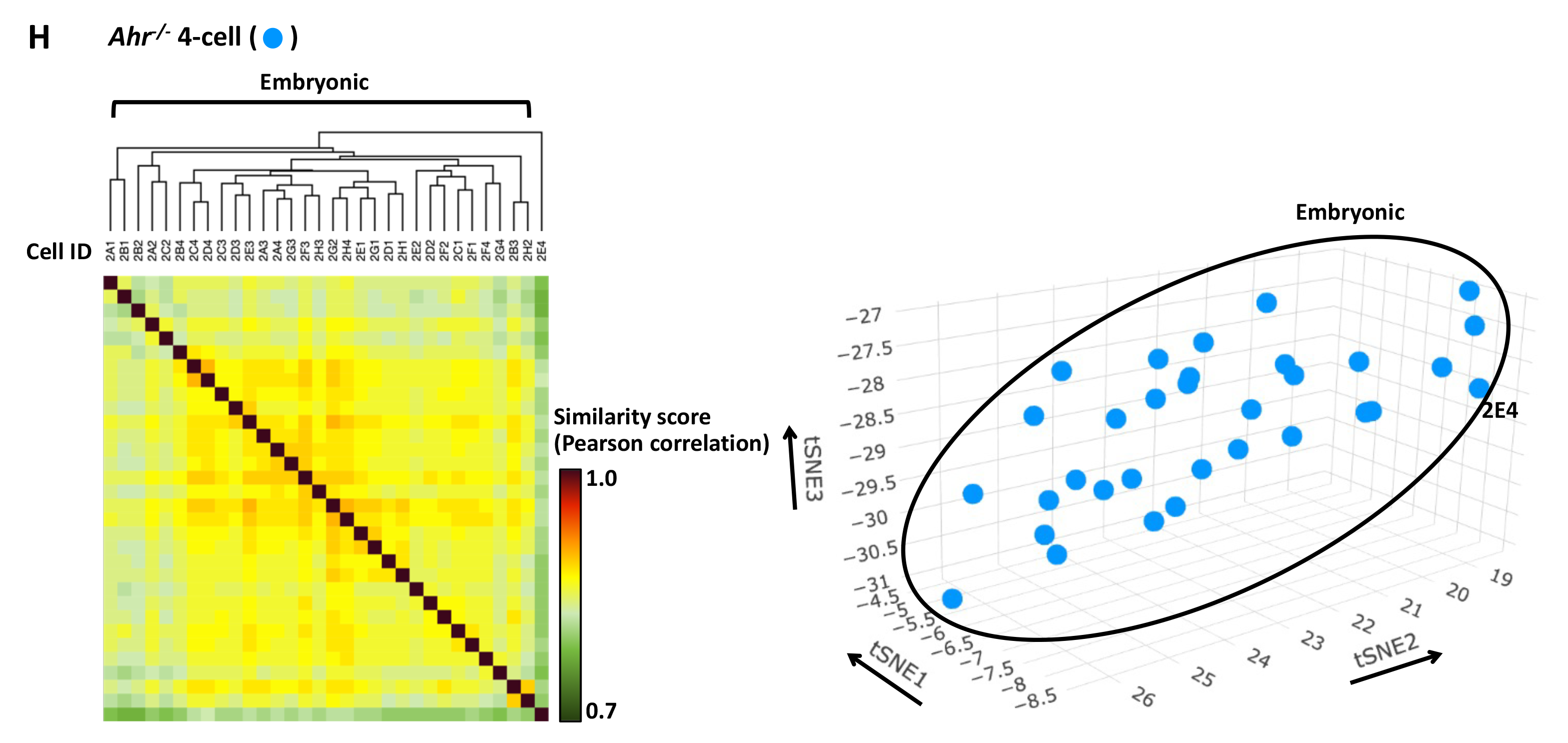
**

**Supplemental Fig. 1H.** Identification of differentiating blastomeres in the bulk of *Ahr^-/-^* 4-cell blastomere population**.**

**
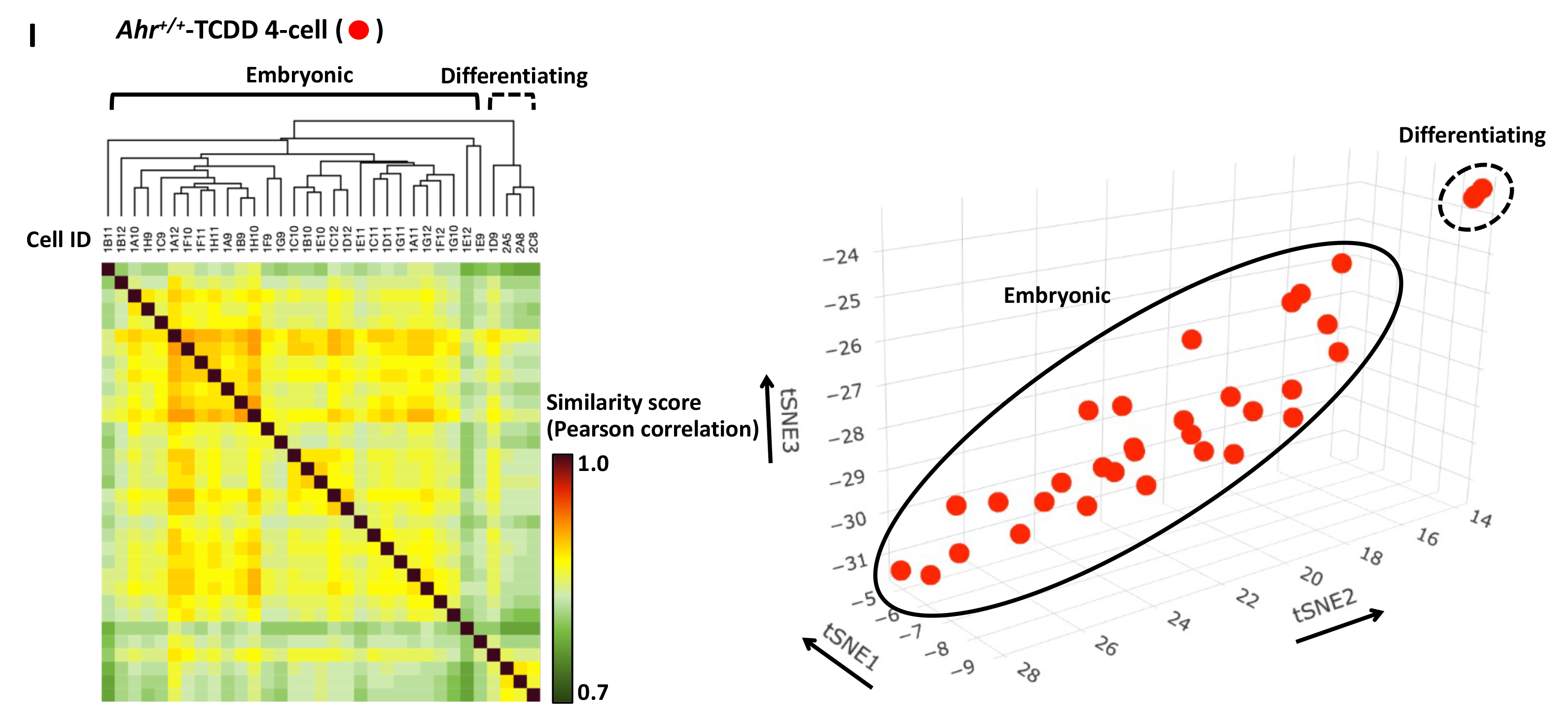
**

**Supplemental Fig. 1I.** Identification of differentiating blastomeres in the bulk of *Ahr^+/+^*-TCDD 4-cell blastomere population.

**
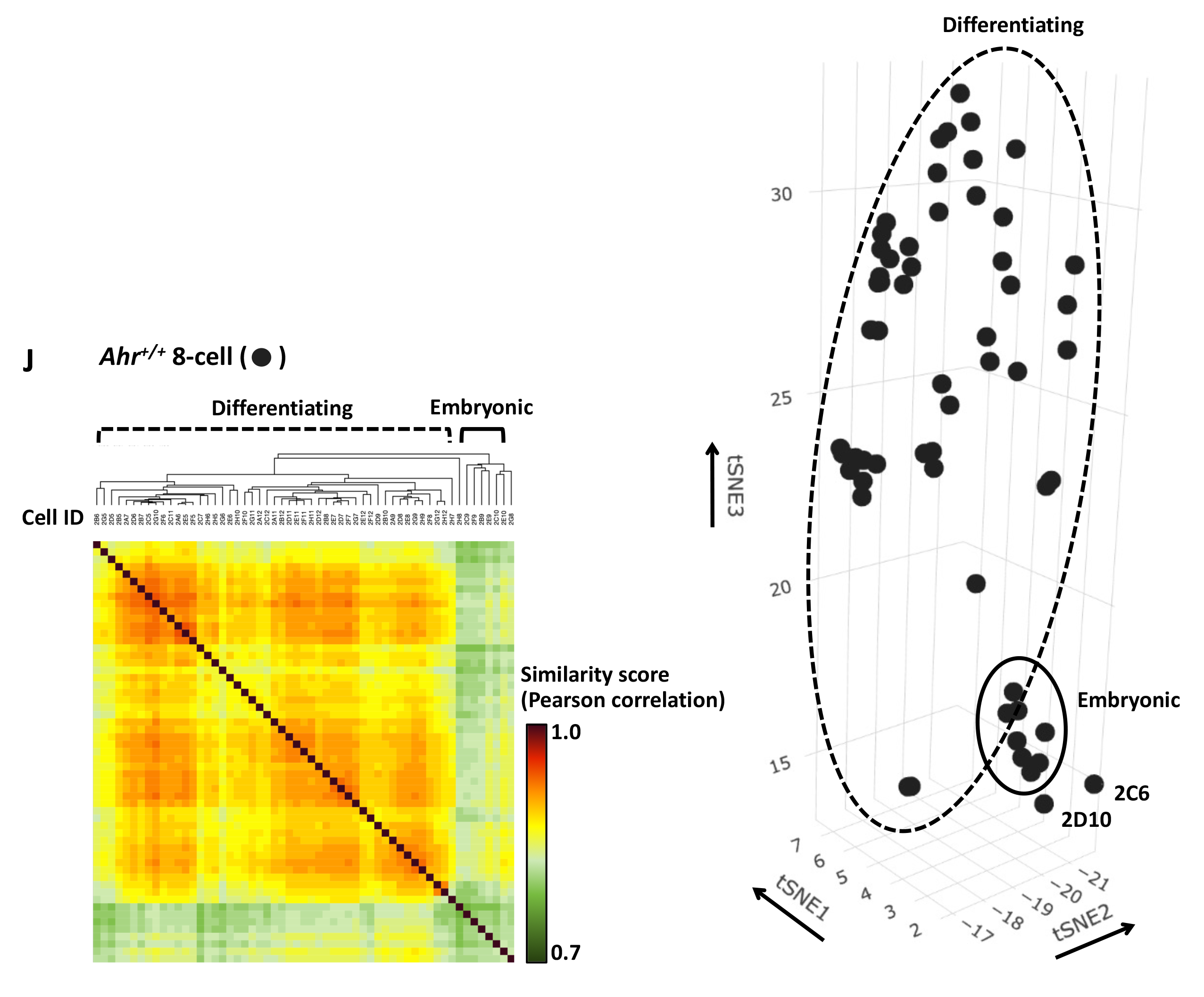
**

**Supplemental Fig. 1J.** Identification of differentiating blastomeres in the bulk of *Ahr^+/+^* 8-cell blastomere population.

**
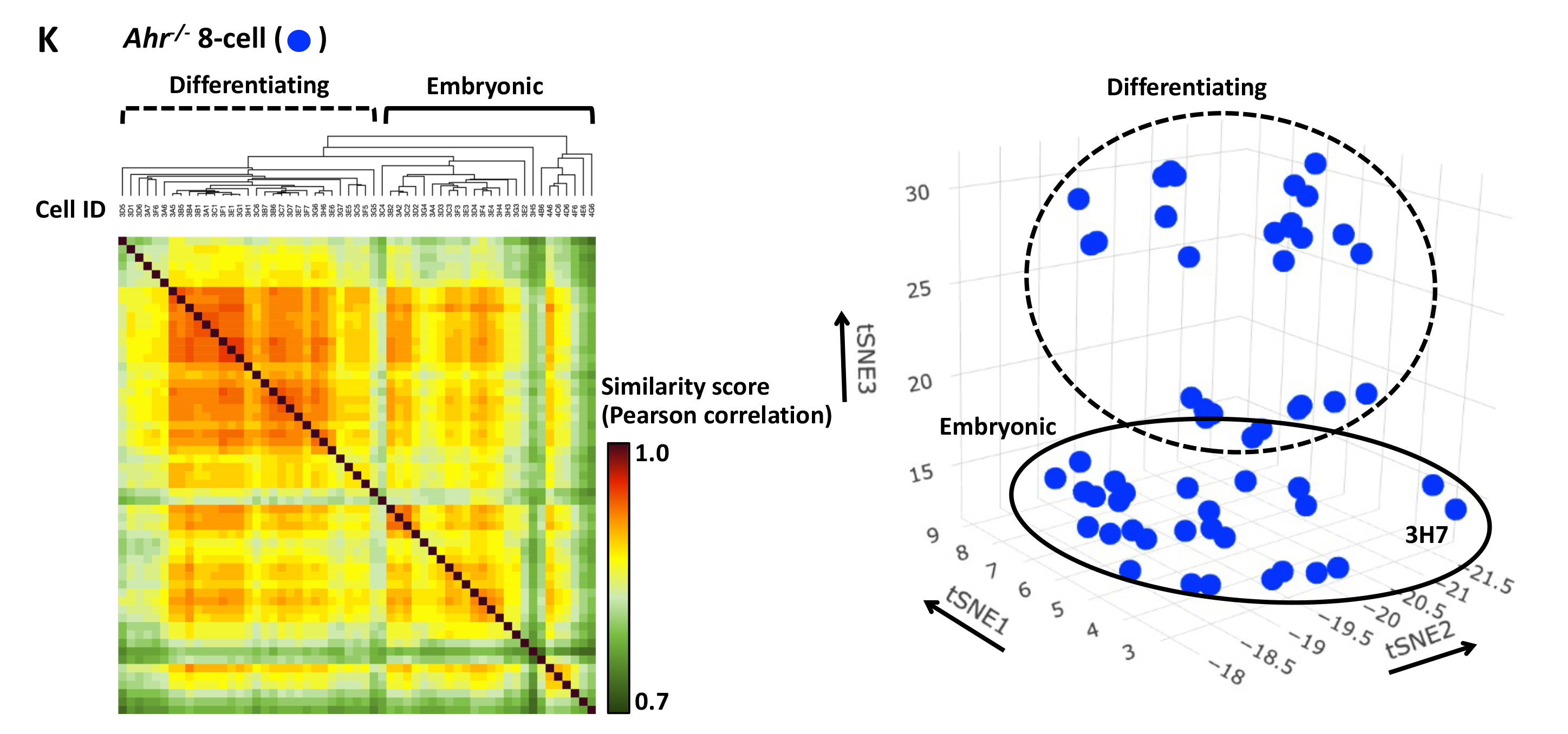
**

**Supplemental Fig. 1K.** Identification of differentiating blastomeres in the bulk of *Ahr^-/-^* 8-cell blastomere population.

**
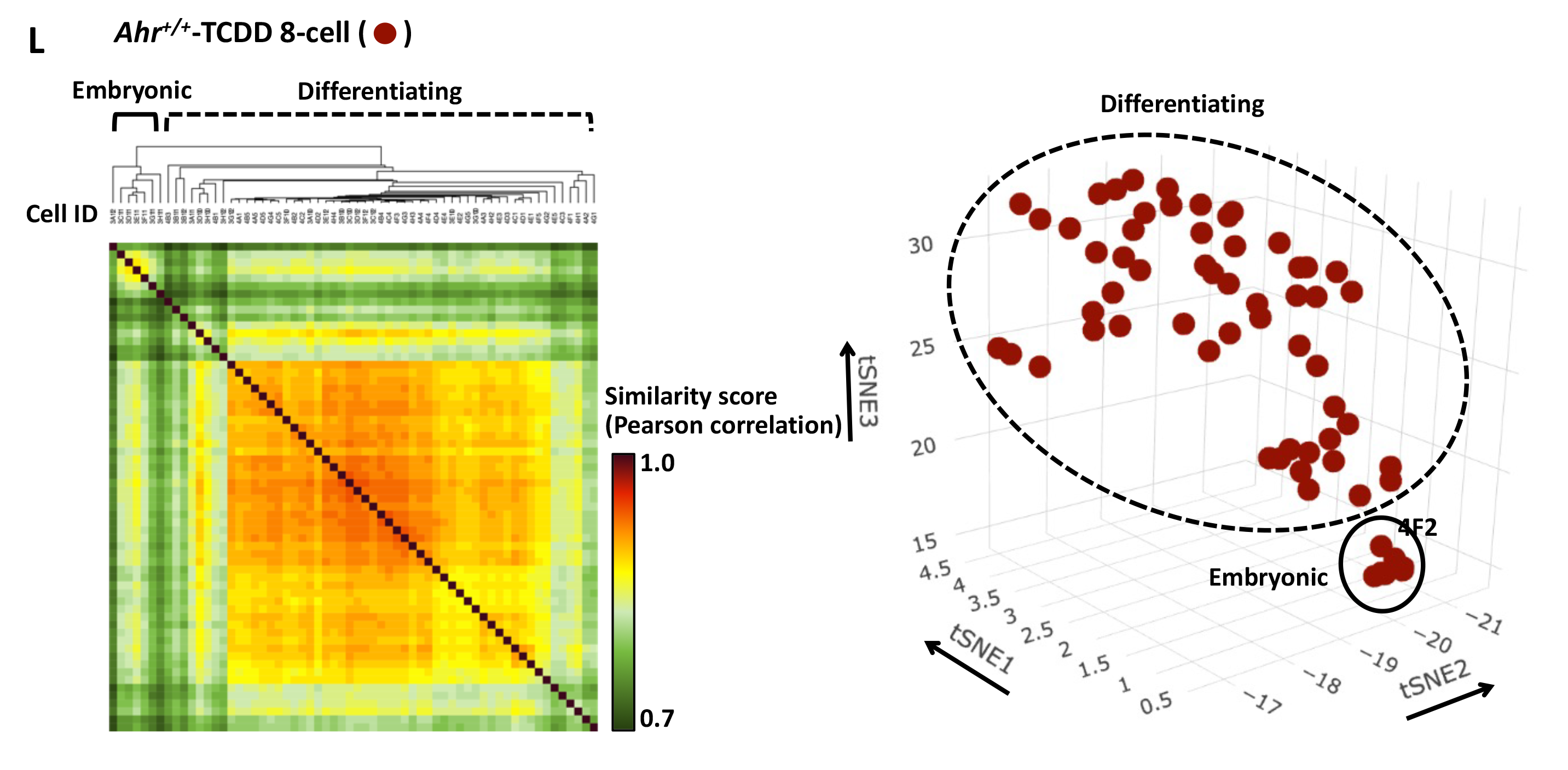
**

**Supplemental Fig. 1L.** Identification of differentiating blastomeres in the bulk of *Ahr^+/+^*-TCDD 8-cell blastomere population.

**Supplemental Fig. 1M.** Table recapitulating the number of embryonic and differentiating blastomeres in each of the 9 groups.**
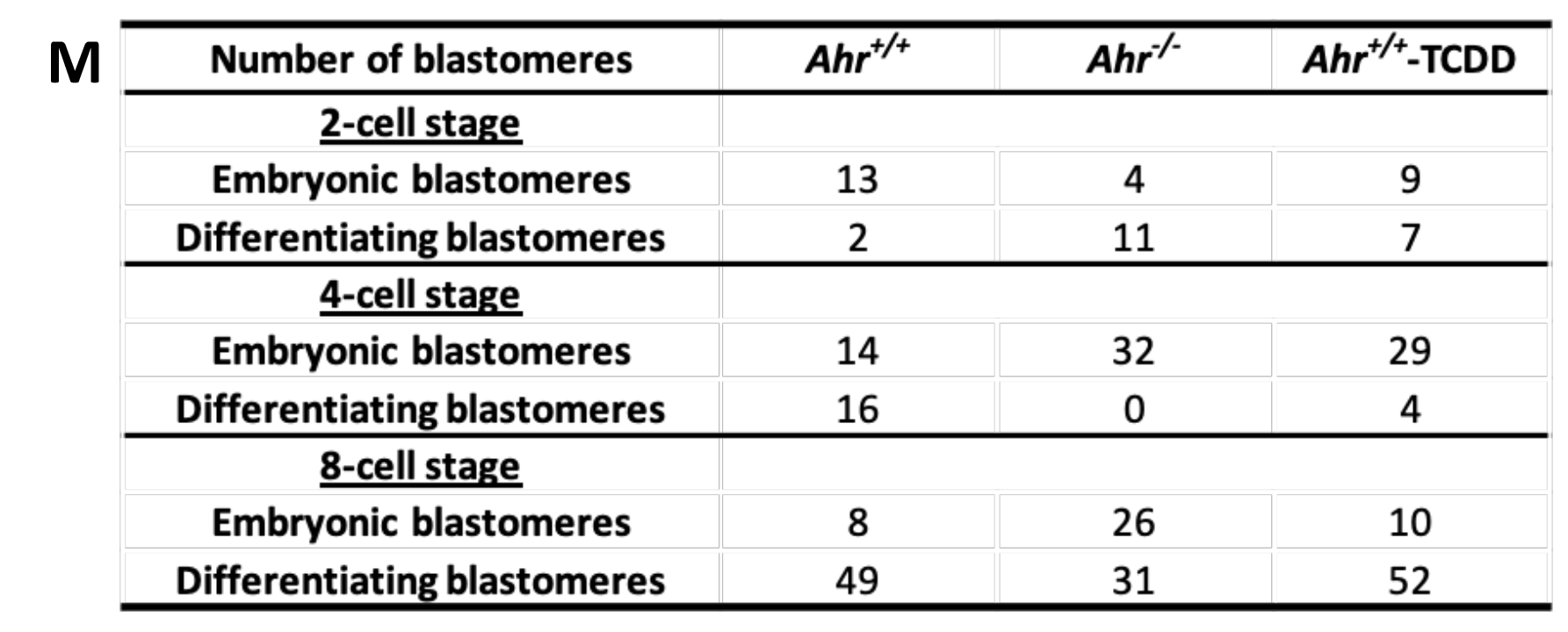
**

**Supplemental Fig. 1N.
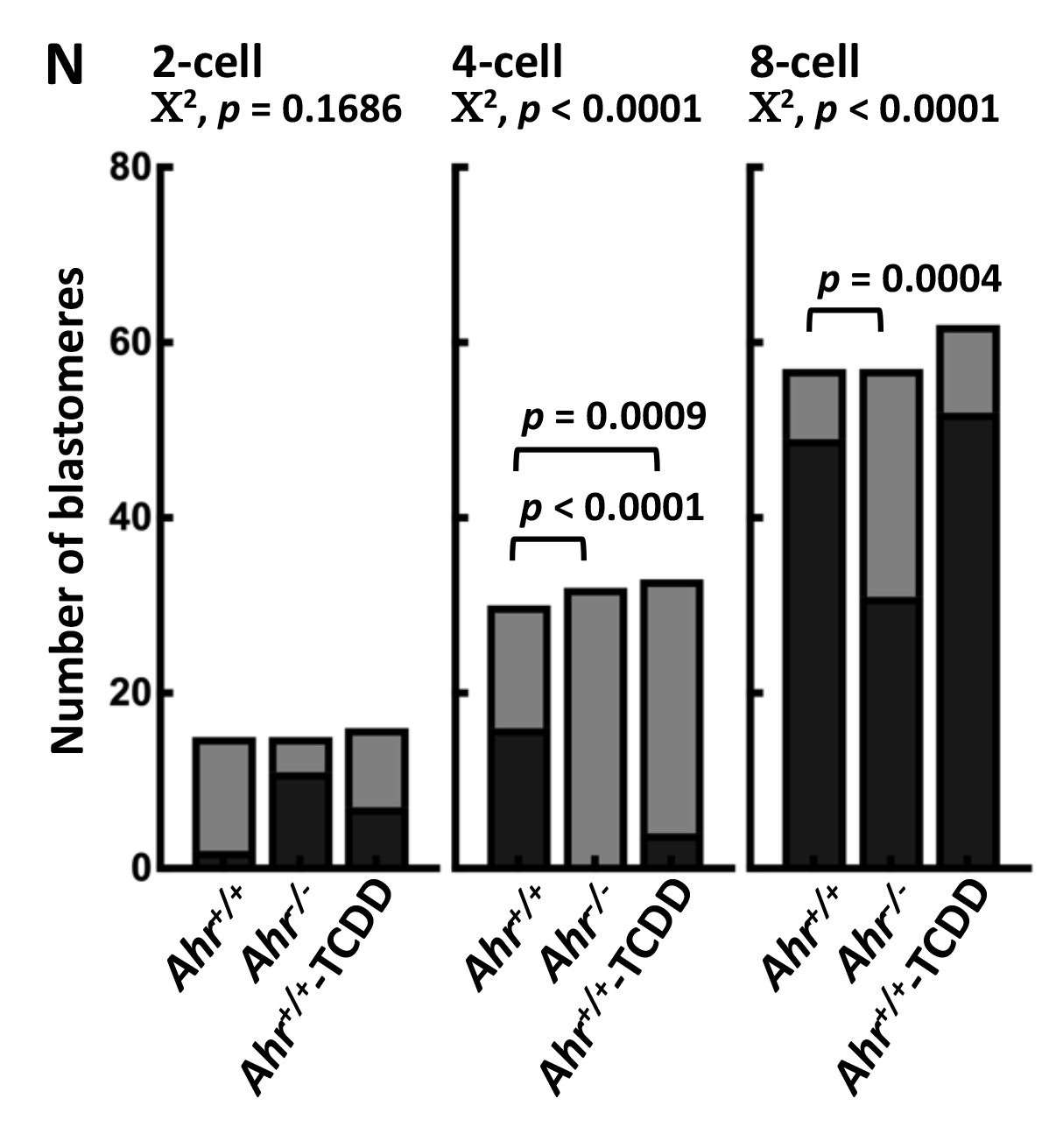
**Verification of significance of the number of differentiating blastomeres identified in each of the 9 groups by Chi-square test followed by Fisher’s exact test.

**Supplemental Figure 2. Differentially Enriched Canonical Pathways Identified in *Ahr^+/+^* and *Ahr^-/-^* embryonic development.**

**
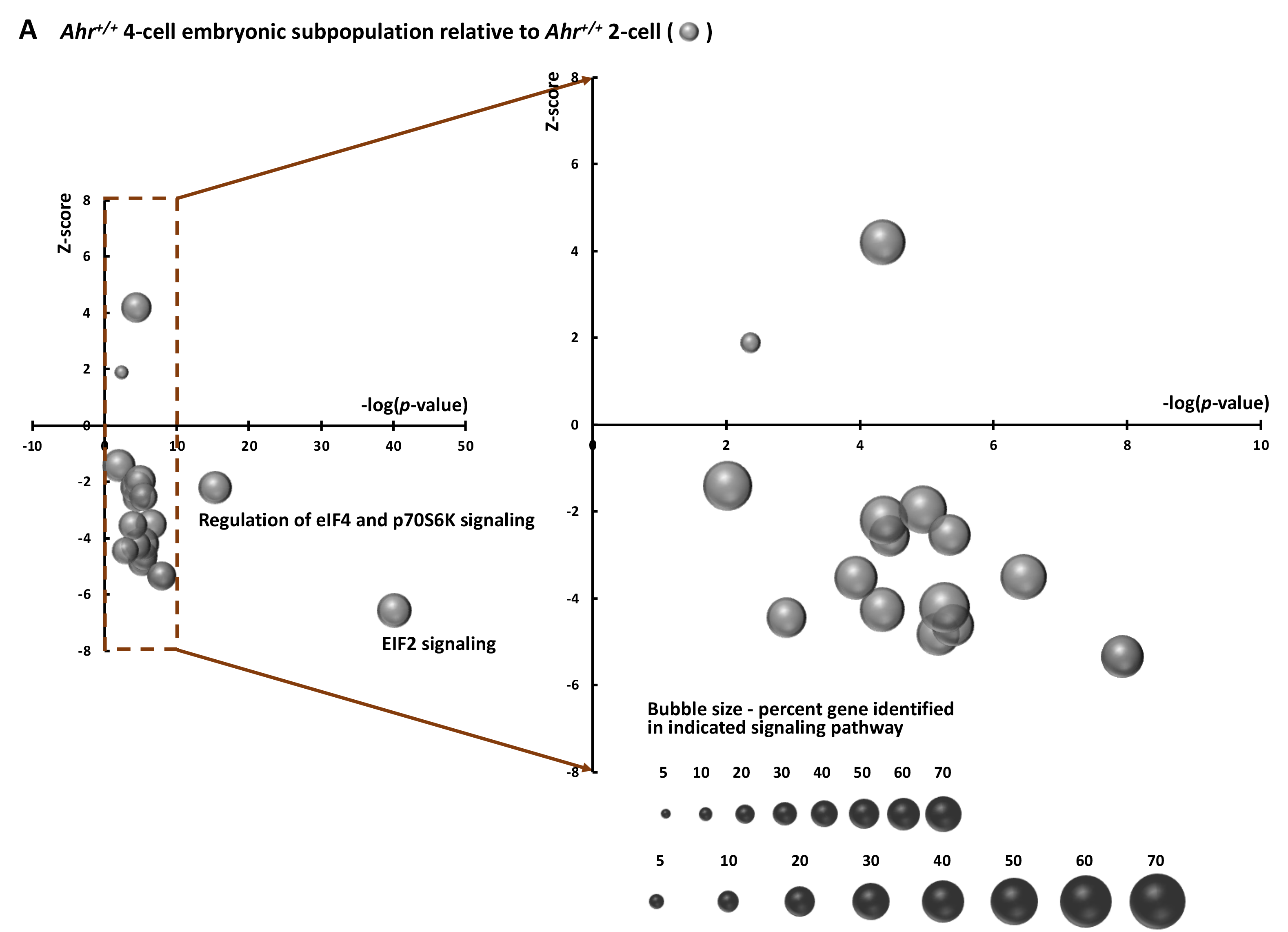
**

**Supplemental Fig. 2A.** Differentially enriches canonical pathways identified in the comparison of *Ahr^+/+^* 4-cell embryonic subpopulation to 2-cell blastomeres.


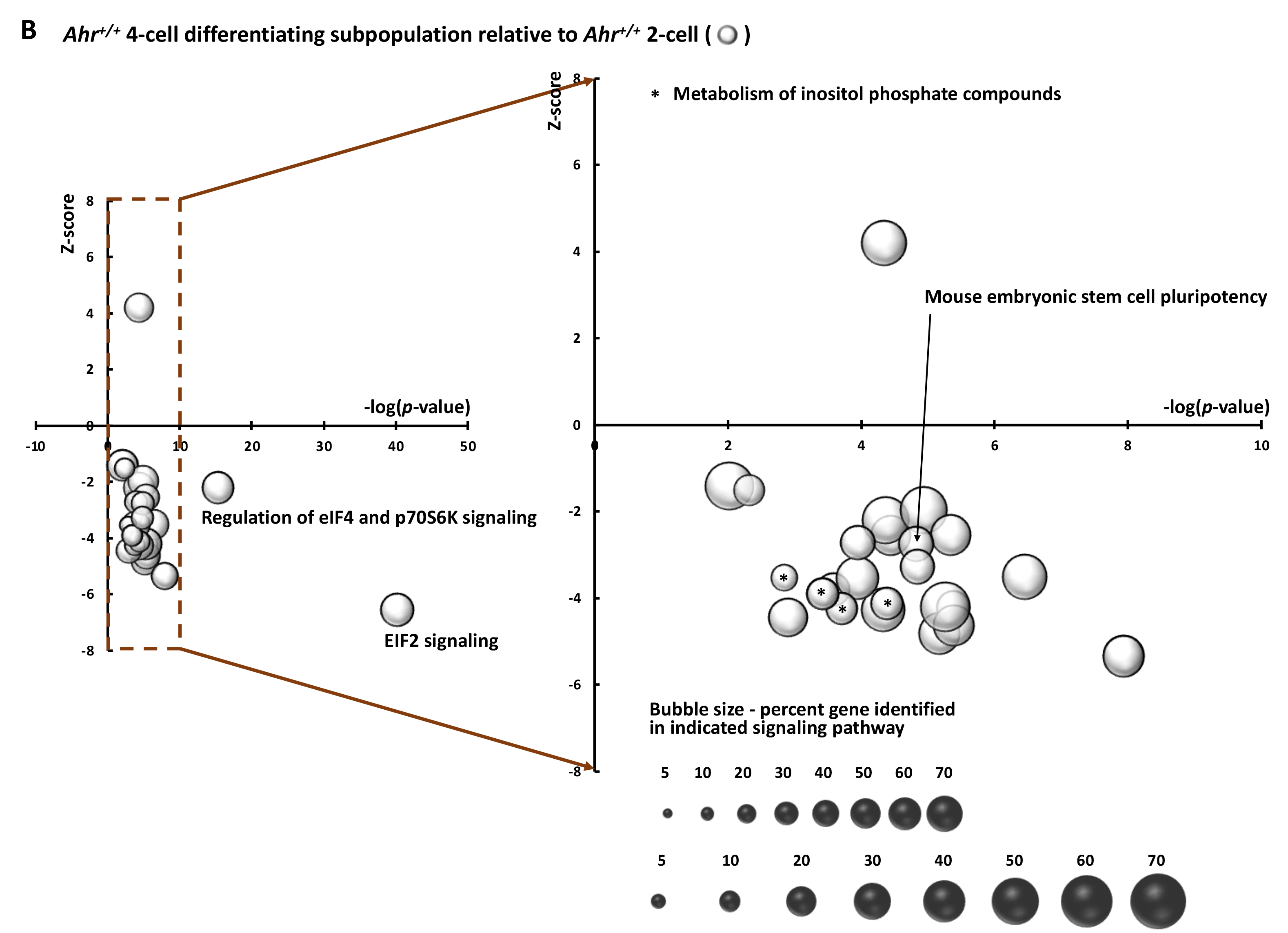


**Supplemental Fig. 2B.** Differentially enriches canonical pathways identified in the comparison of *Ahr^+/+^* 4-cell differentiating subpopulation to 2-cell blastomeres.


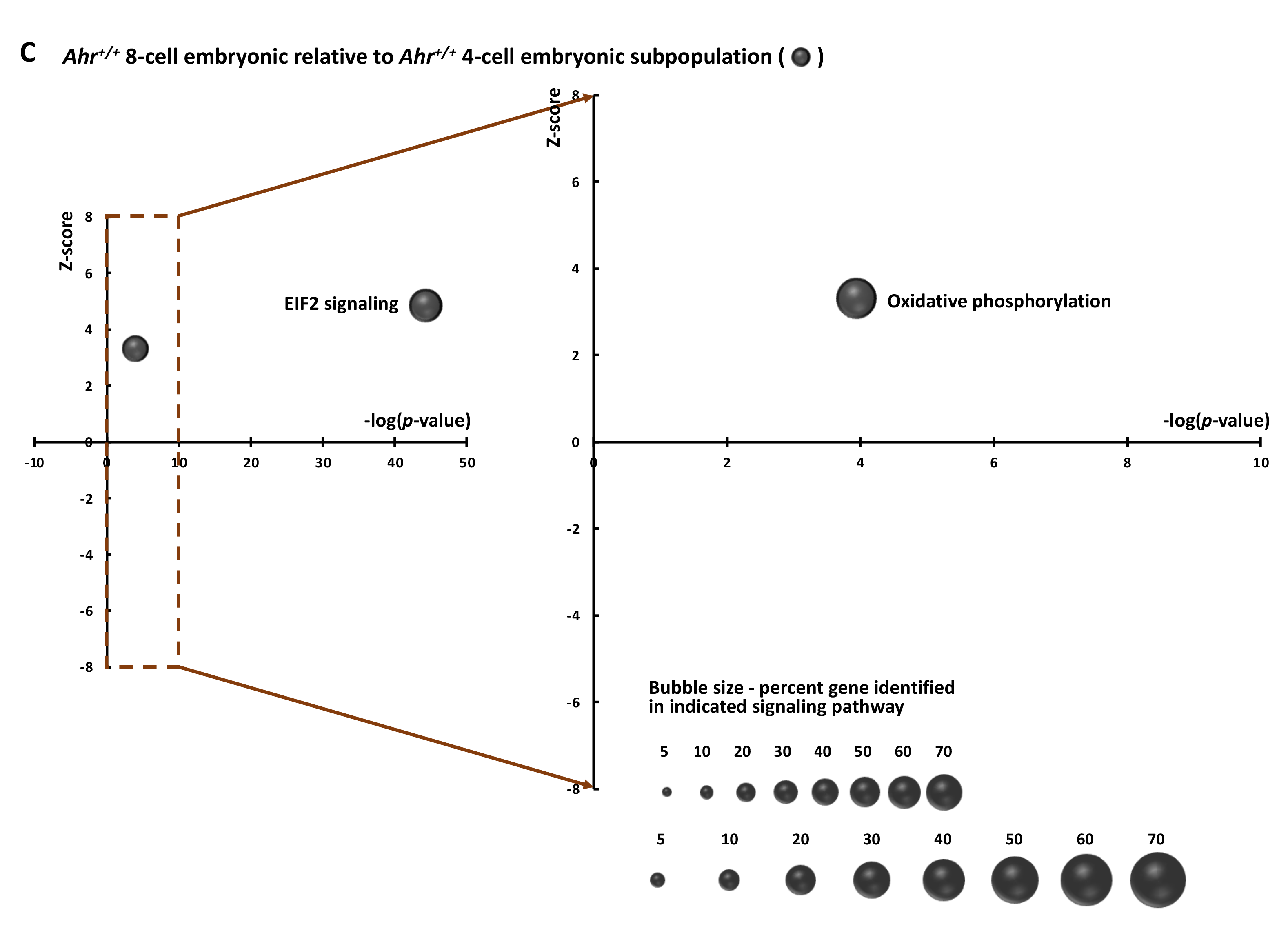


**Supplemental Fig. 2C.** Differentially enriches canonical pathways identified in the comparison of *Ahr^+/+^* 8-cell embryonic to 4-cell embryonic subpopulation.


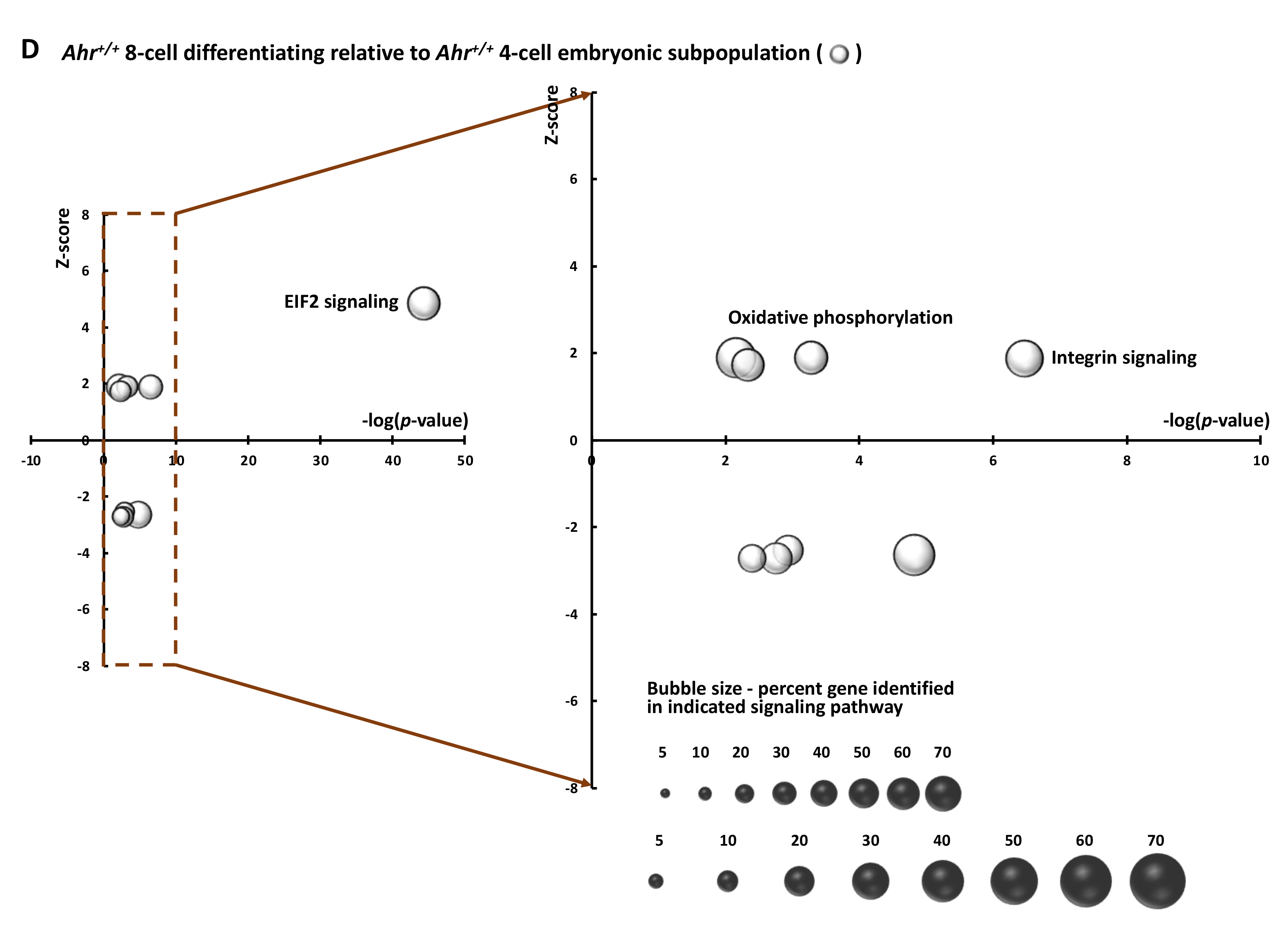


**Supplemental Fig. 2D.** Differentially enriches canonical pathways identified in the comparison of *Ahr^+/+^* 8-cell differentiating to 4-cell embryonic subpopulation.


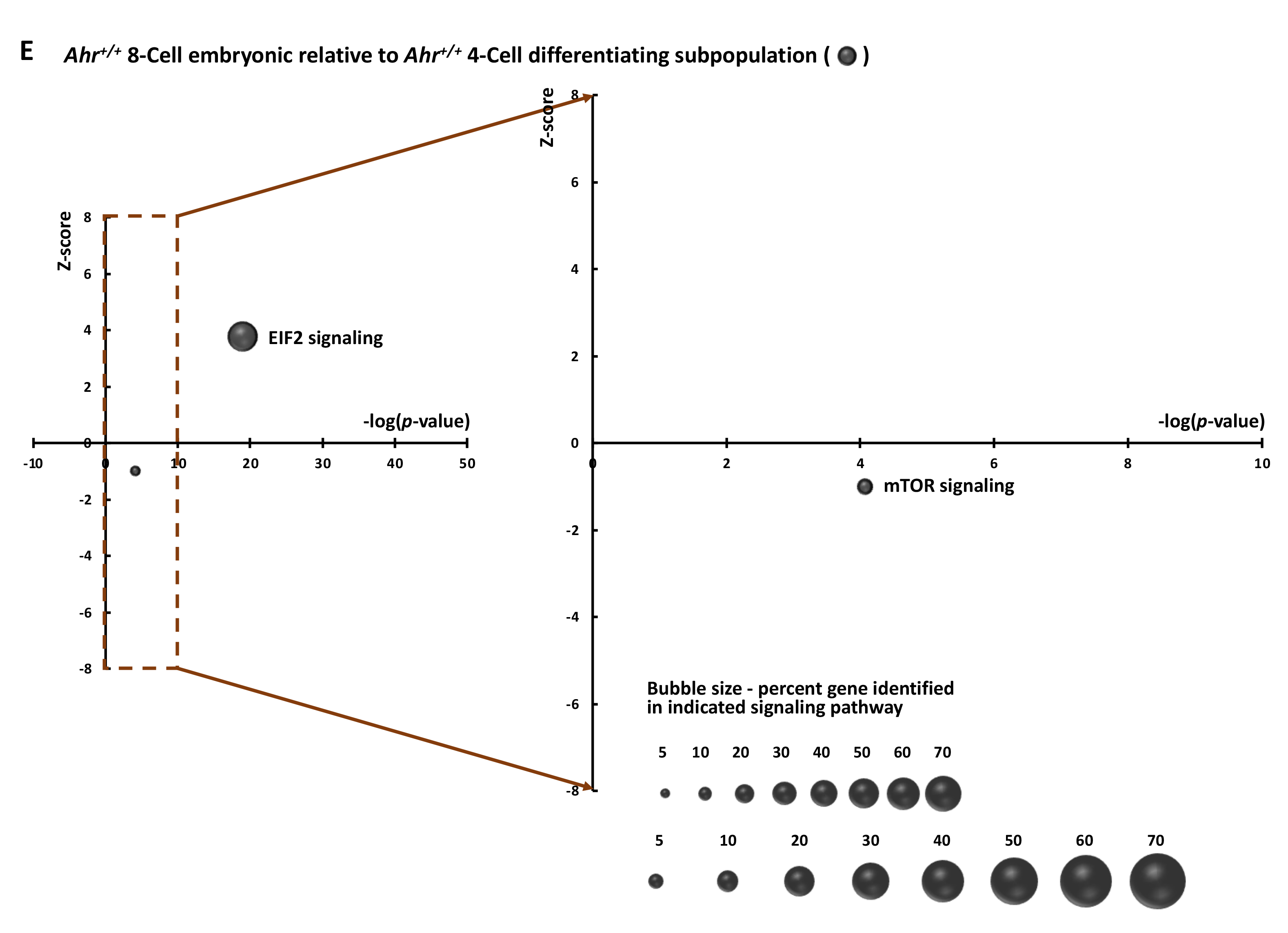


**Supplemental Fig. 2E.** Differentially enriches canonical pathways identified in the comparison of *Ahr^+/+^* 8-cell embryonic to 4-cell differentiating subpopulation.


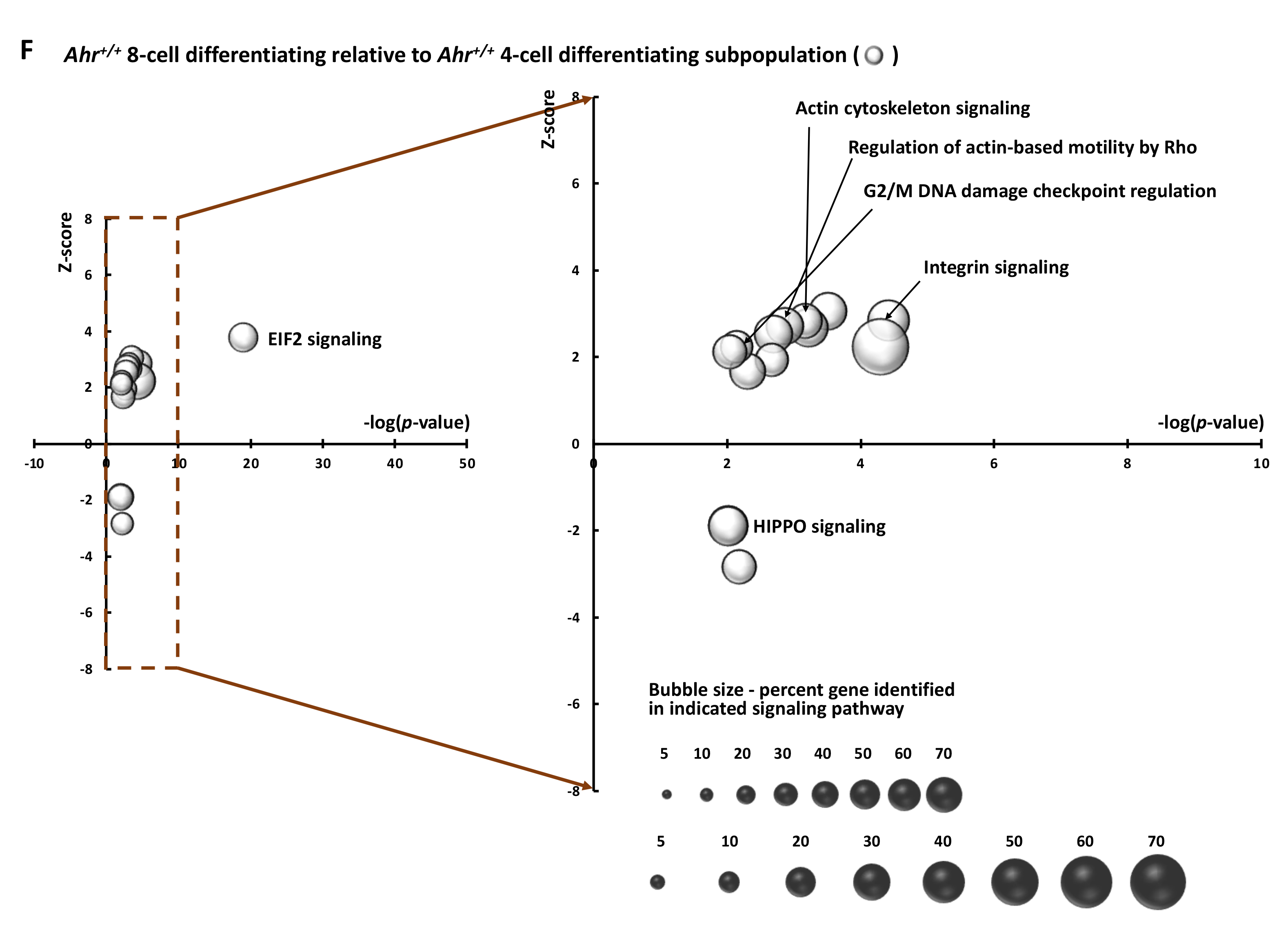


**Supplemental Fig. 2F.** Differentially enriches canonical pathways identified in the comparison of *Ahr^+/+^* 8-cell differentiating to 4-cell differentiating subpopulation.


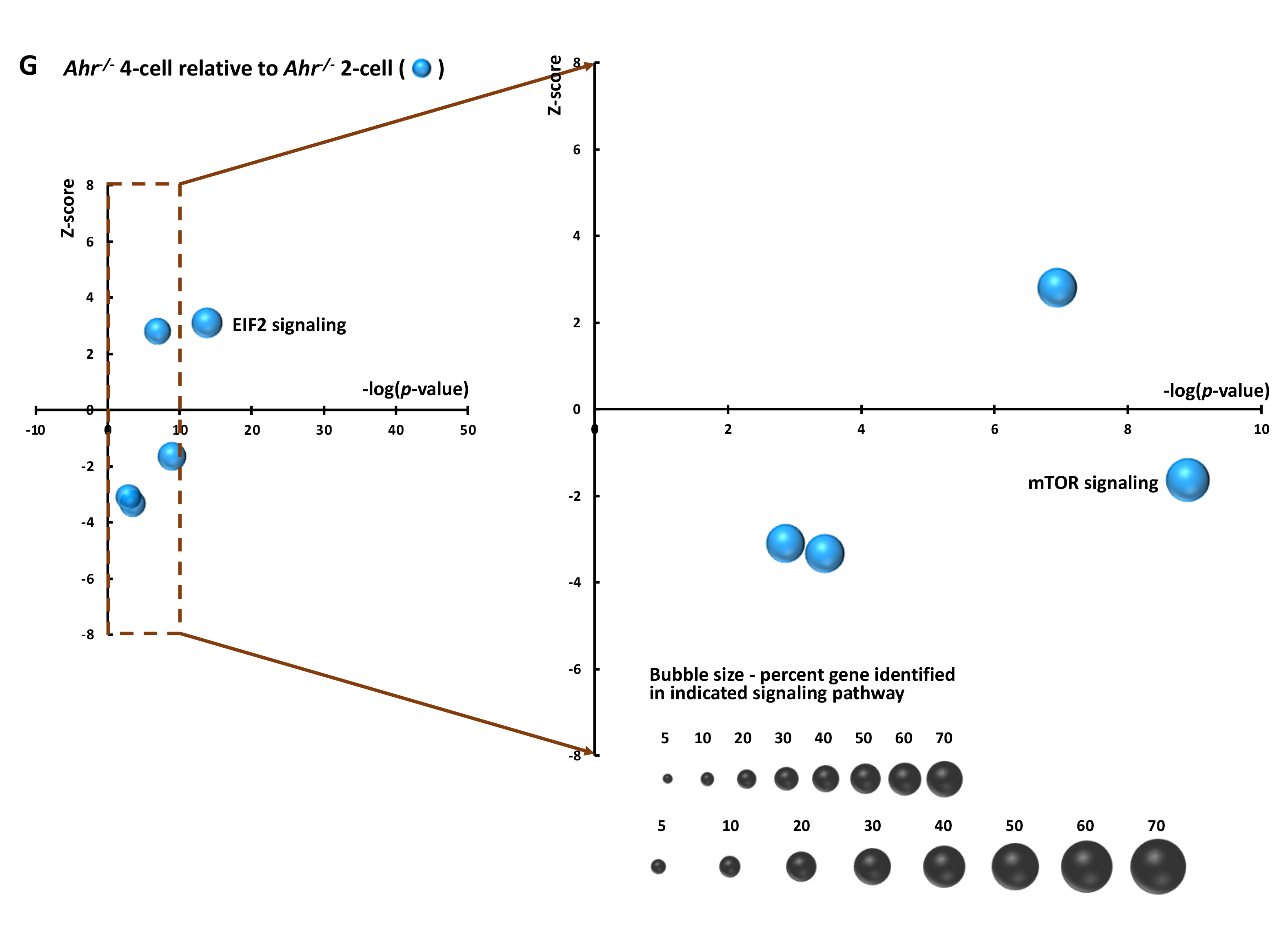


**Supplemental Fig. 2G.** Differentially enriches canonical pathways identified in the comparison of *Ahr^-/-^* 4-cell embryonic subpopulation to 2-cell blastomeres.


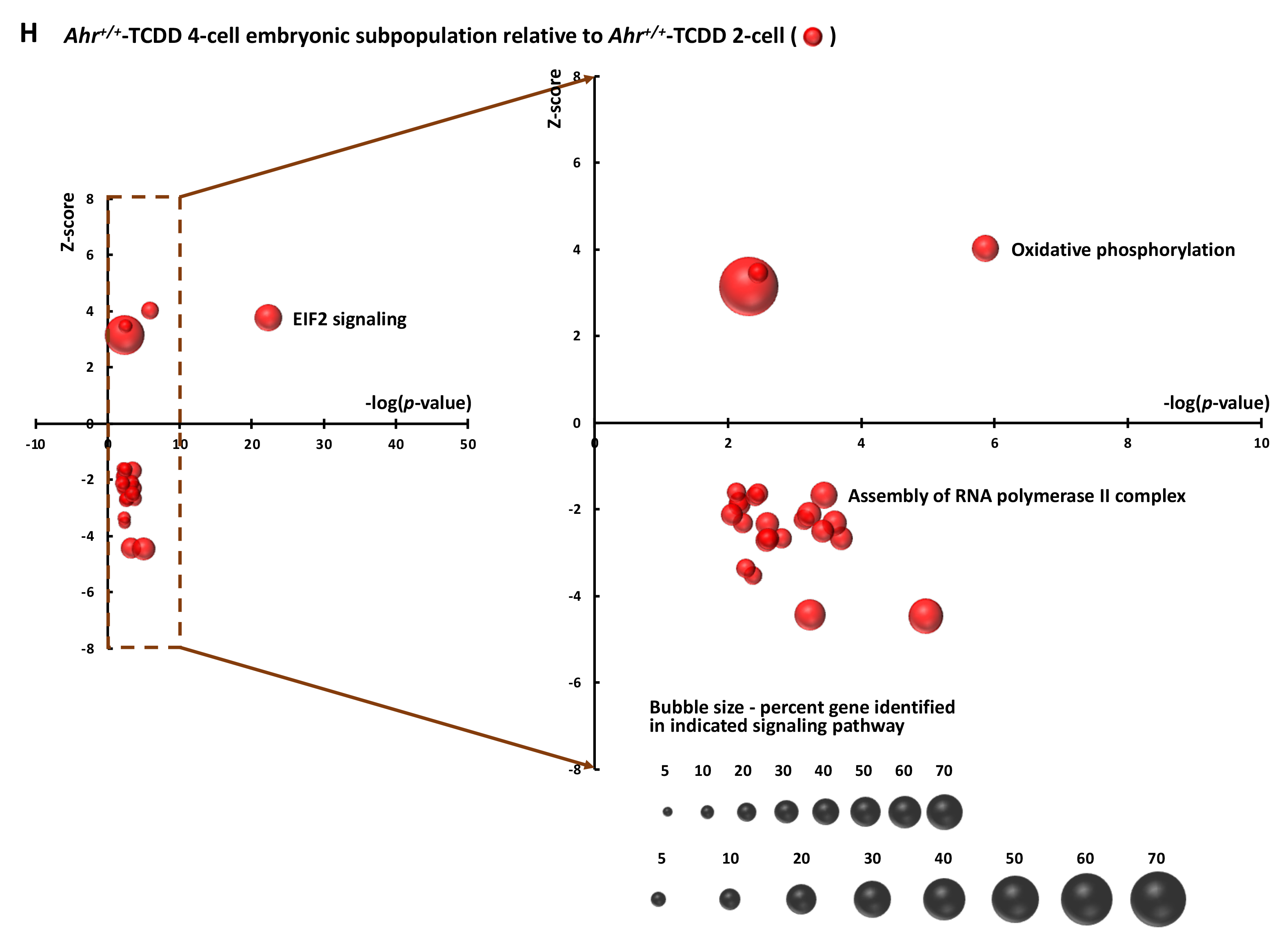


**Supplemental Fig. 2H.** Differentially enriches canonical pathways identified in the comparison of *Ahr^+/+^*-TCDD 4-cell embryonic subpopulation to 2-cell blastomeres.


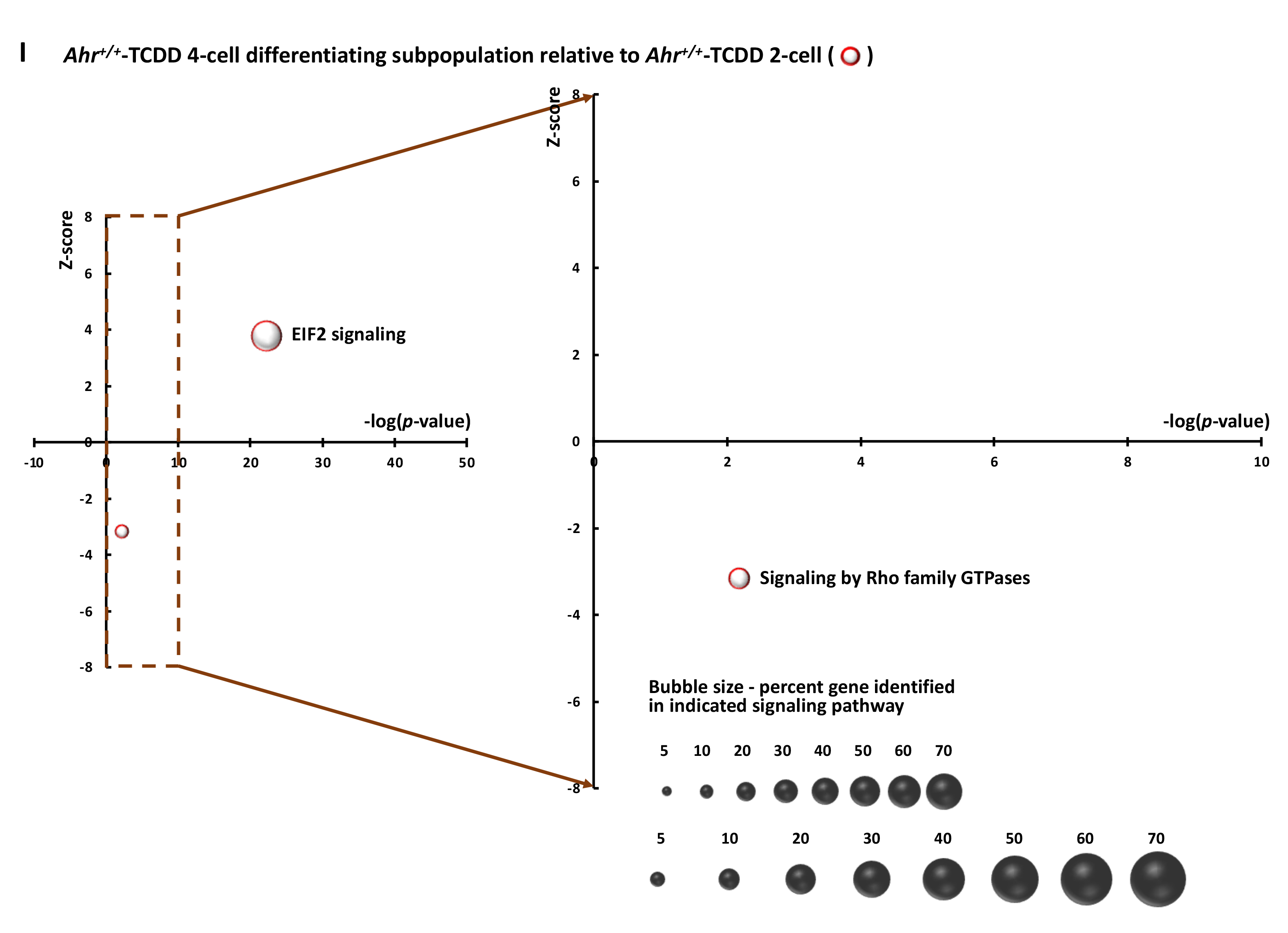


**Supplemental Fig. 2I.** Differentially enriches canonical pathways identified in the comparison of *Ahr^+/+^*-TCDD 4-cell differentiating subpopulation to 2-cell blastomeres.


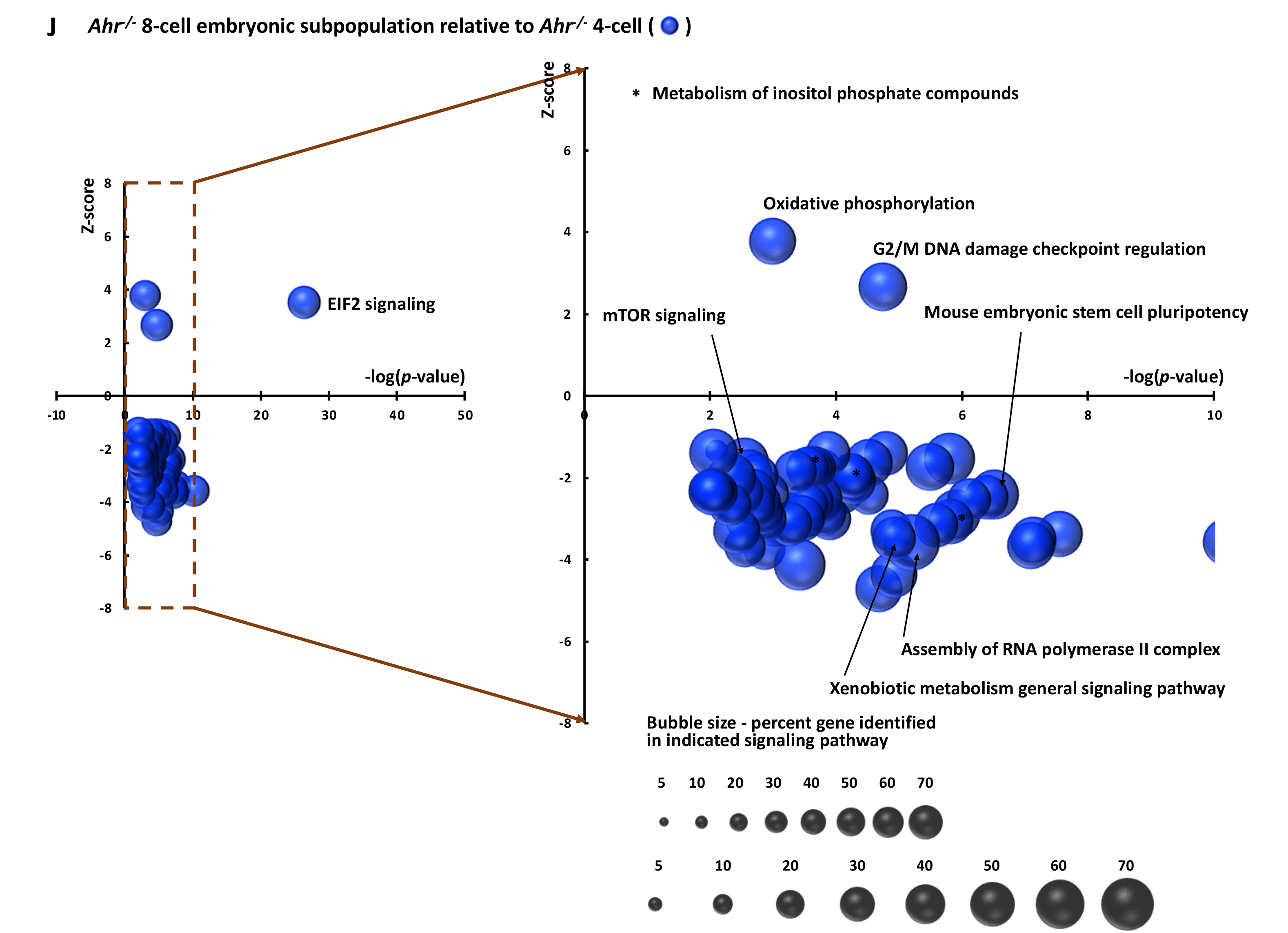


**Supplemental Fig. 2J.** Differentially enriches canonical pathways identified in the comparison of *Ahr^-/-^* 8-cell embryonic subpopulation to 4-cell blastomeres.


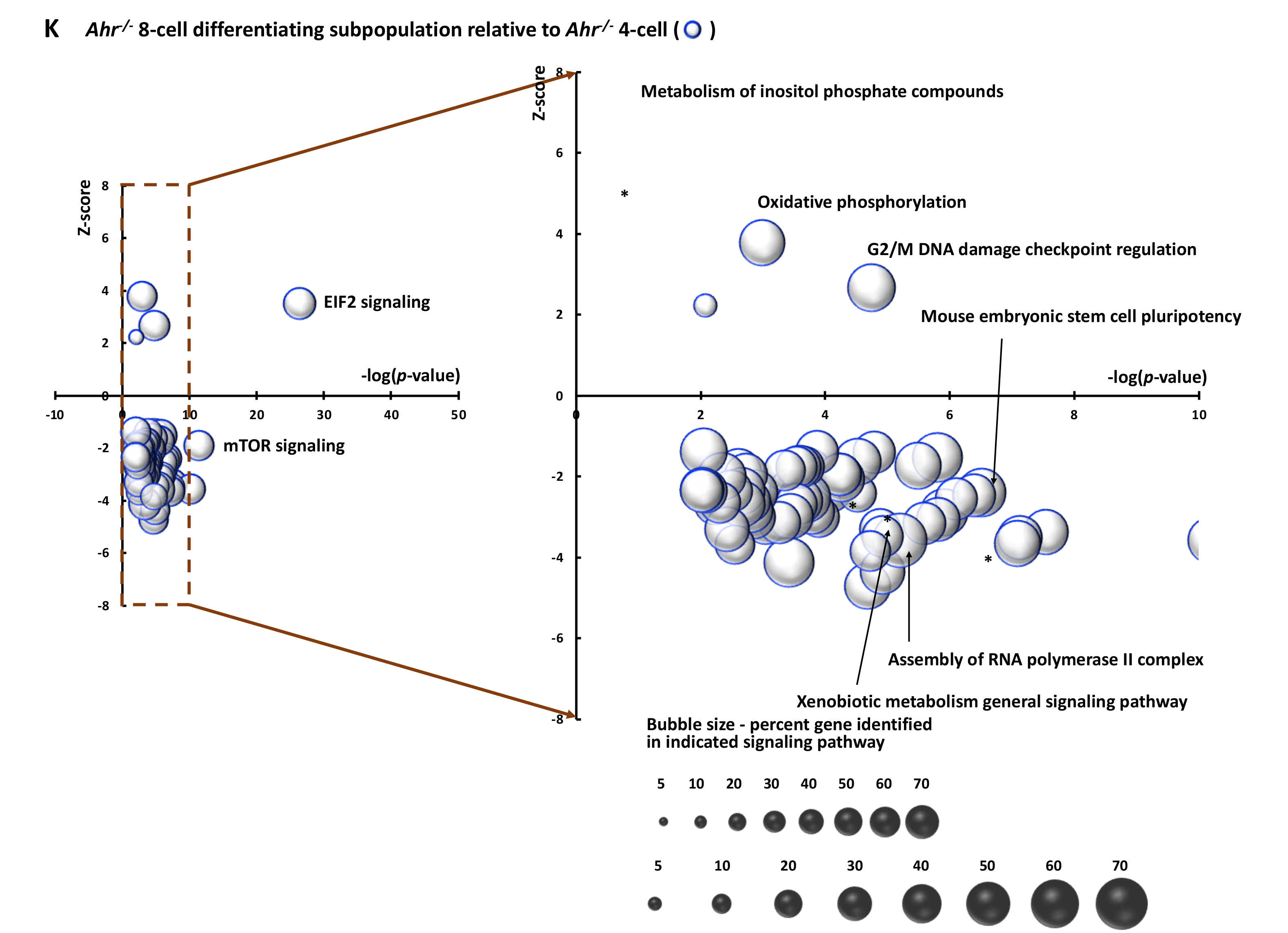


**Supplemental Fig. 2K.** Differentially enriches canonical pathways identified in the comparison of *Ahr^-/-^* 8-cell differentiating subpopulation to 4-cell blastomeres.


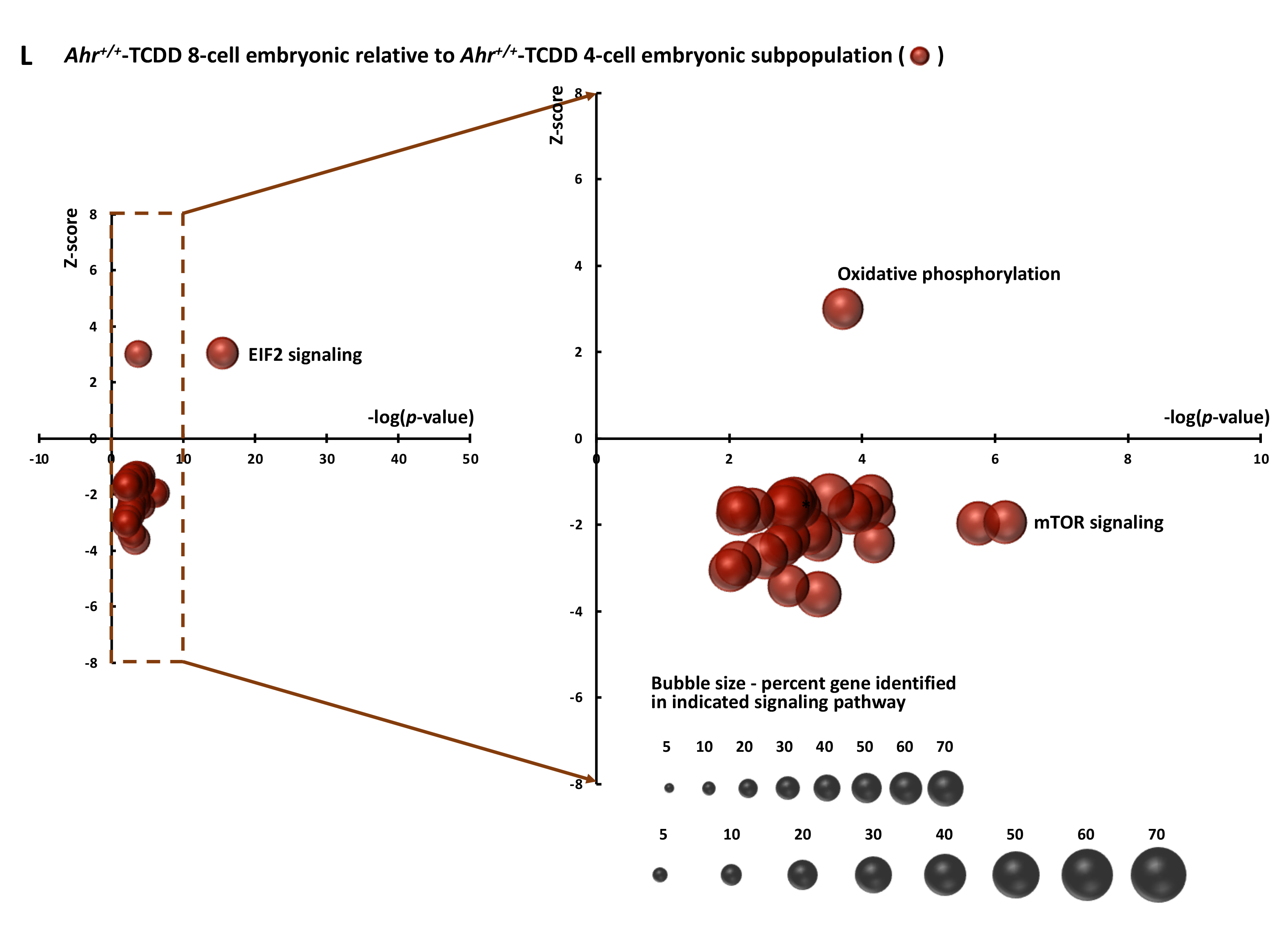


**Supplemental Fig. 2L.** Differentially enriches canonical pathways identified in the comparison of *Ahr^+/+^*-TCDD 8-cell embryonic to 4-cell embryonic subpopulation.


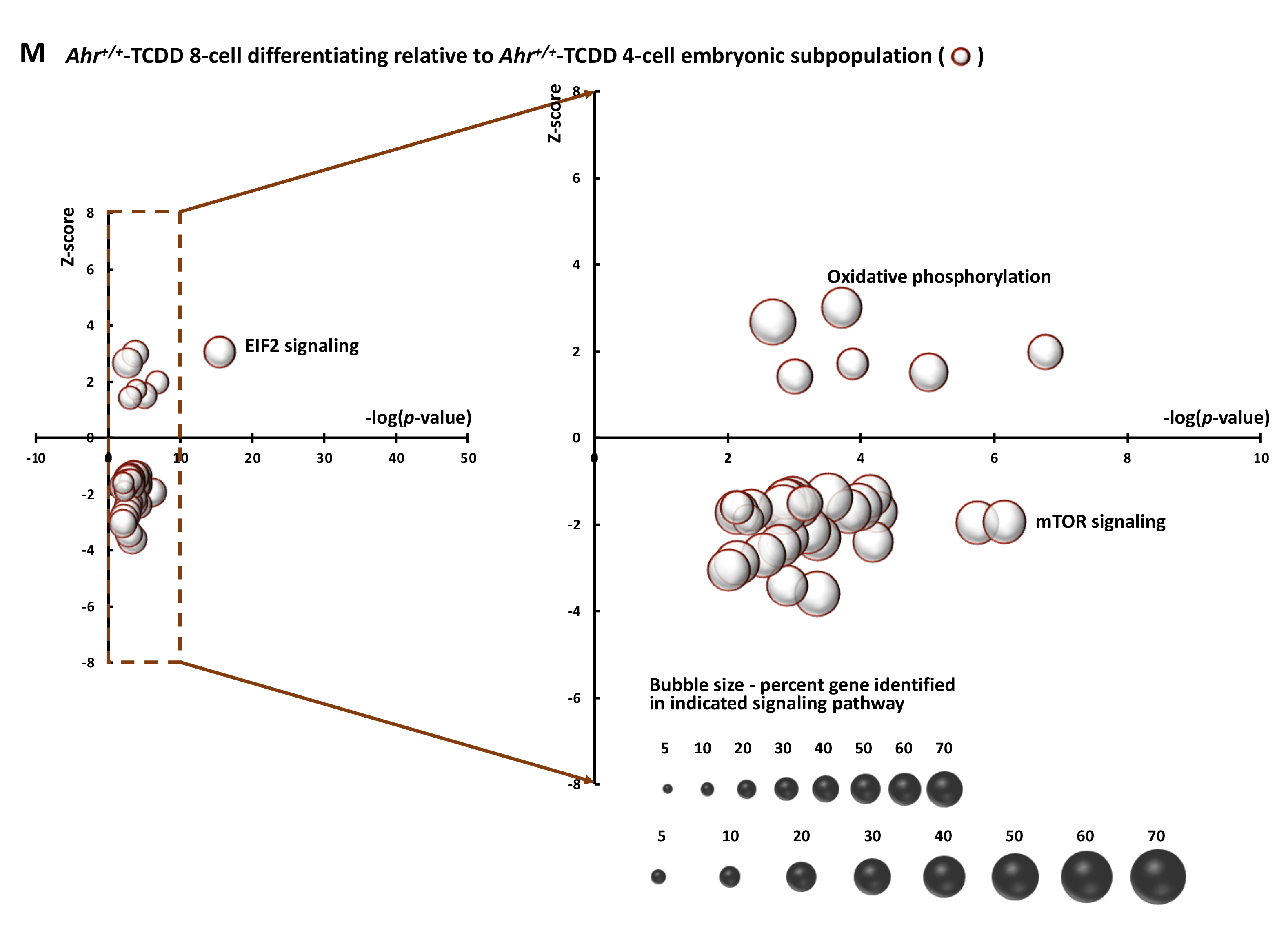


**Supplemental Fig. 2M.** Differentially enriches canonical pathways identified in the comparison of *Ahr^+/+^*-TCDD 8-cell differentiating to 4-cell embryonic subpopulation.


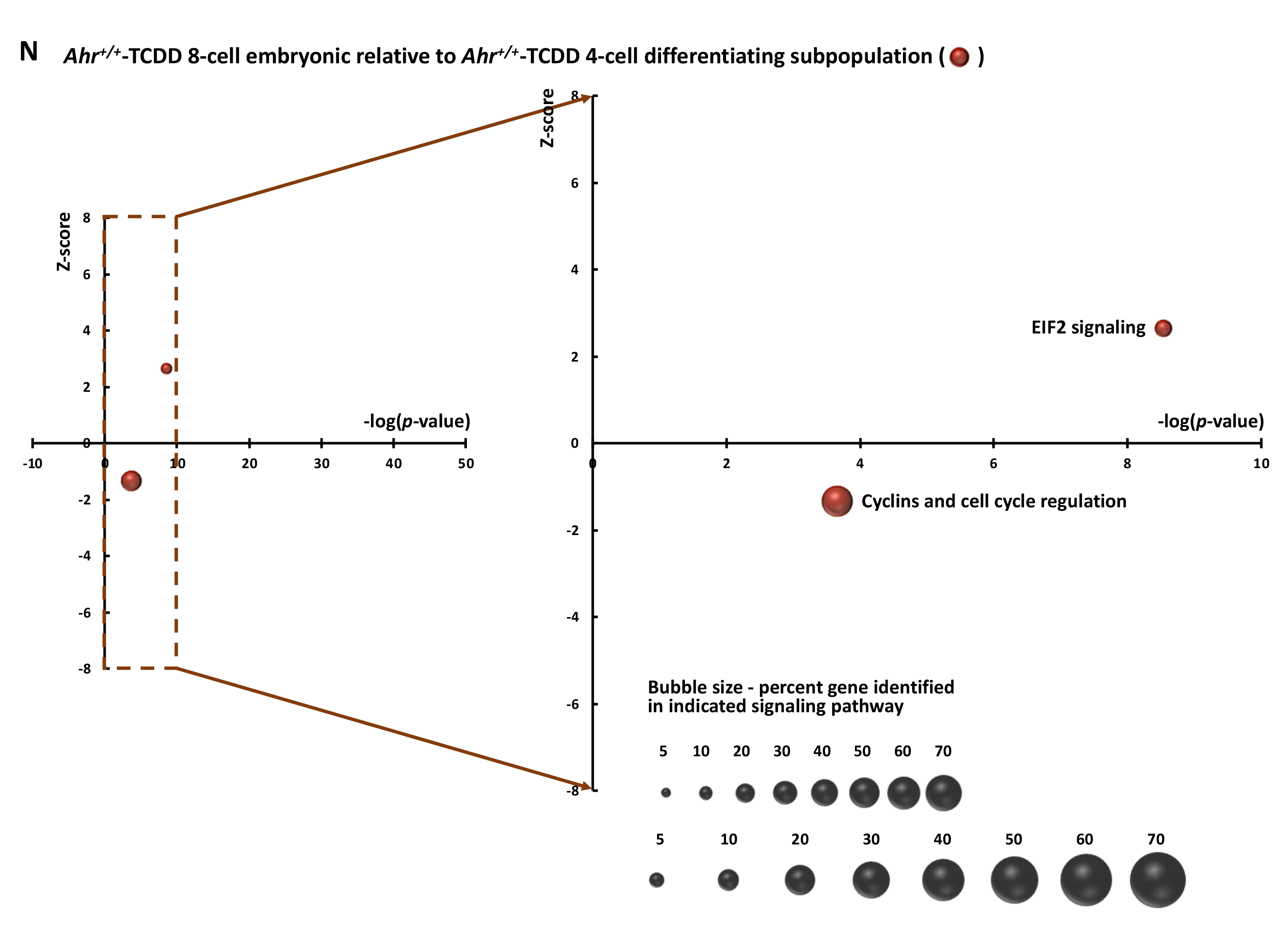


**Supplemental Fig. 2N.** Differentially enriches canonical pathways identified in the comparison of *Ahr^+/+^*-TCDD 8-cell embryonic to 4-cell differentiating subpopulation.


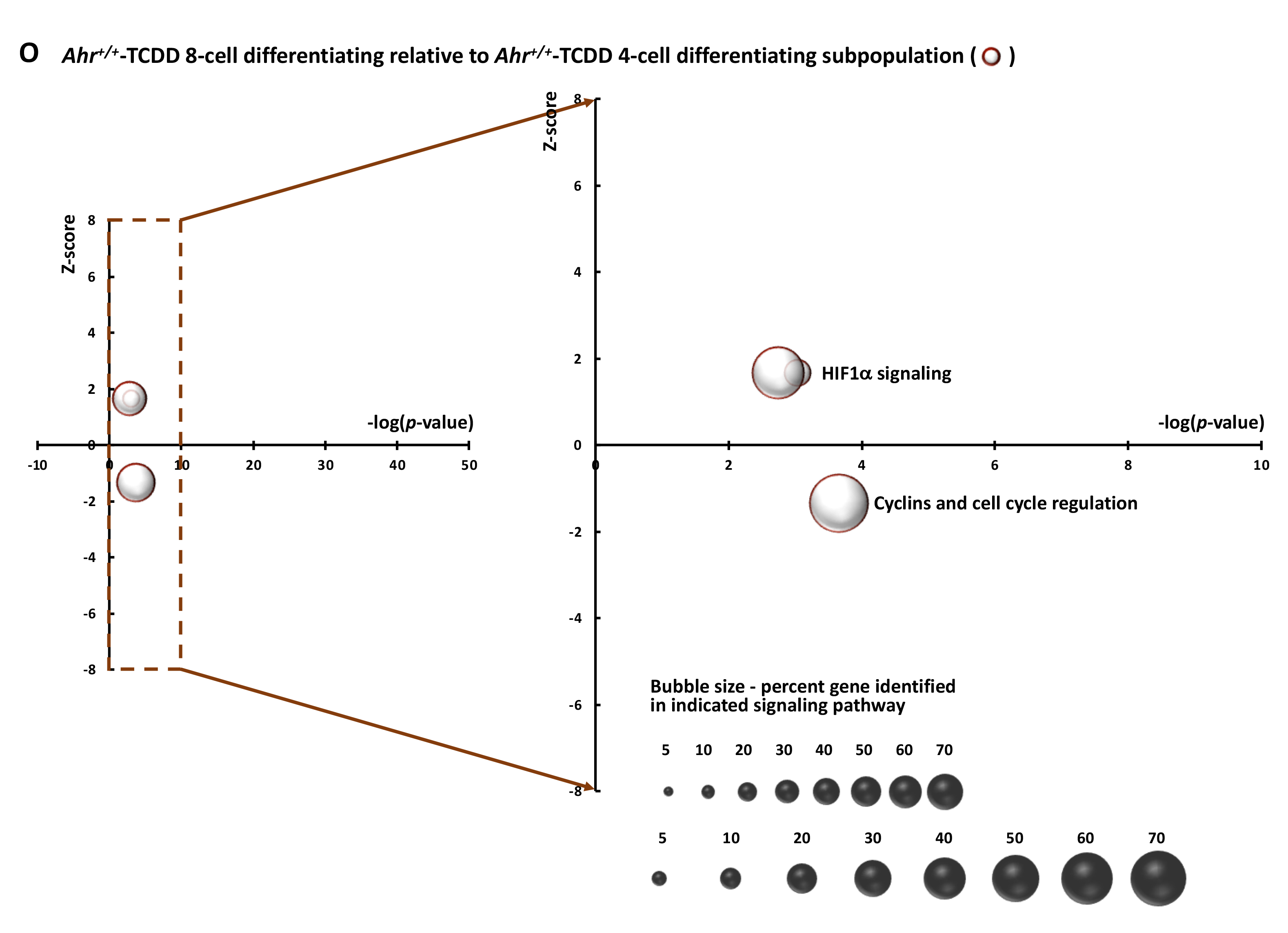


**Supplemental Fig. 2O.** Differentially enriches canonical pathways identified in the comparison of *Ahr^+/+^*-TCDD 8-cell differentiating to 4-cell differentiating subpopulation.

**Supplemental Figure 3. AHR Regulates the Expression Levels and Interblastomere Heterogeneity of OCT4 and CDX2.**


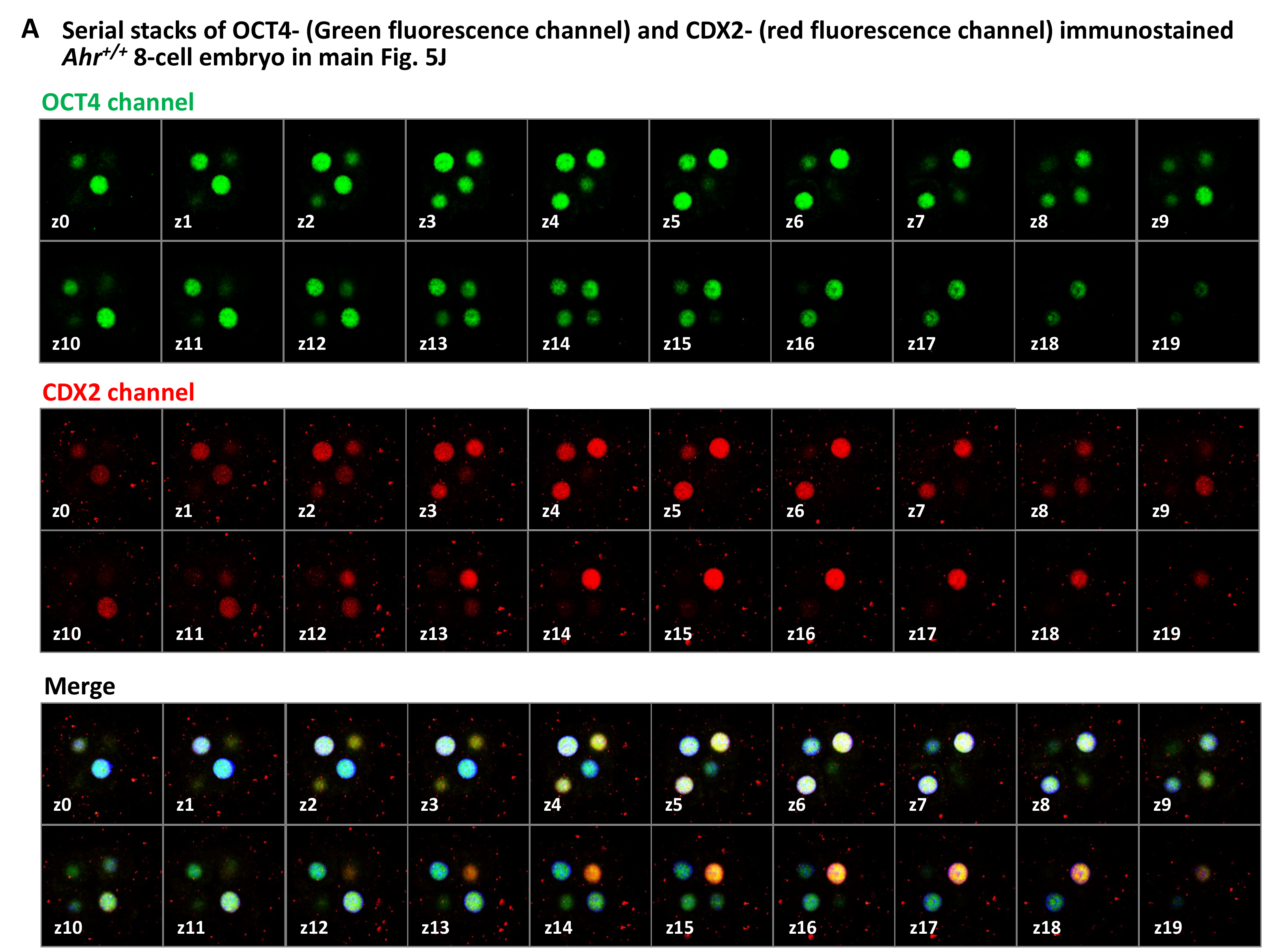


**Supplemental Fig. 3A.** Serial stacks of OCT4- (green fluorescent channel) and CDX2- (red fluorescent channel) immunostained *Ahr^+/+^* 8-cell embryo in main figure 5J.


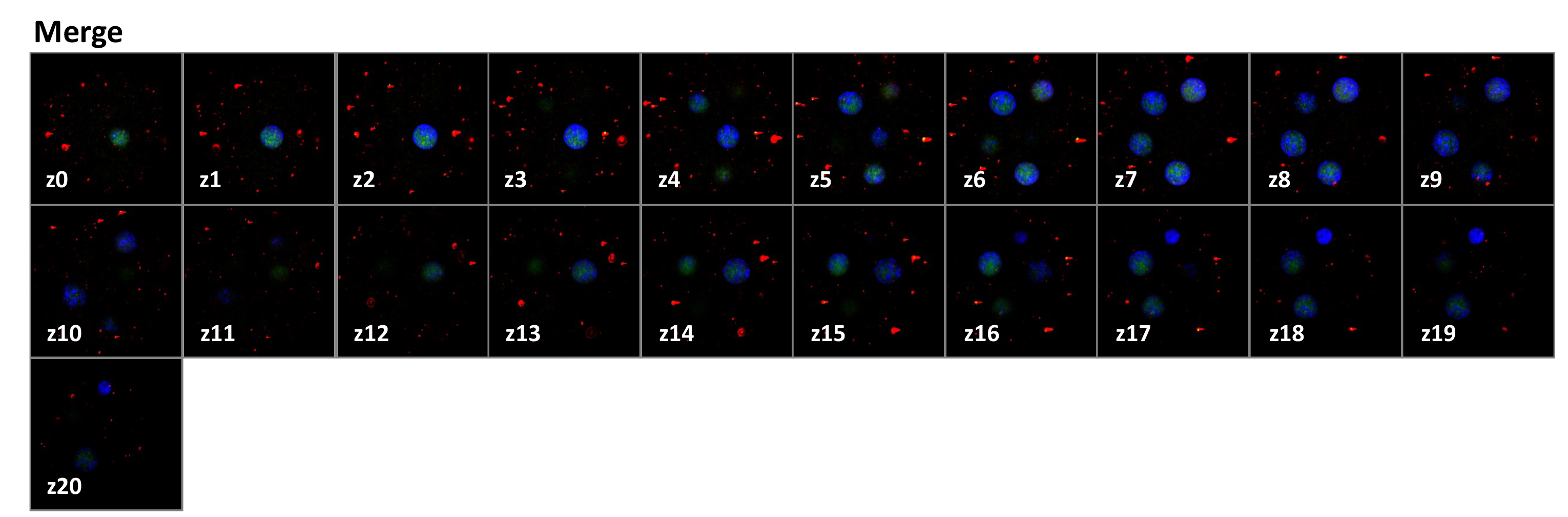

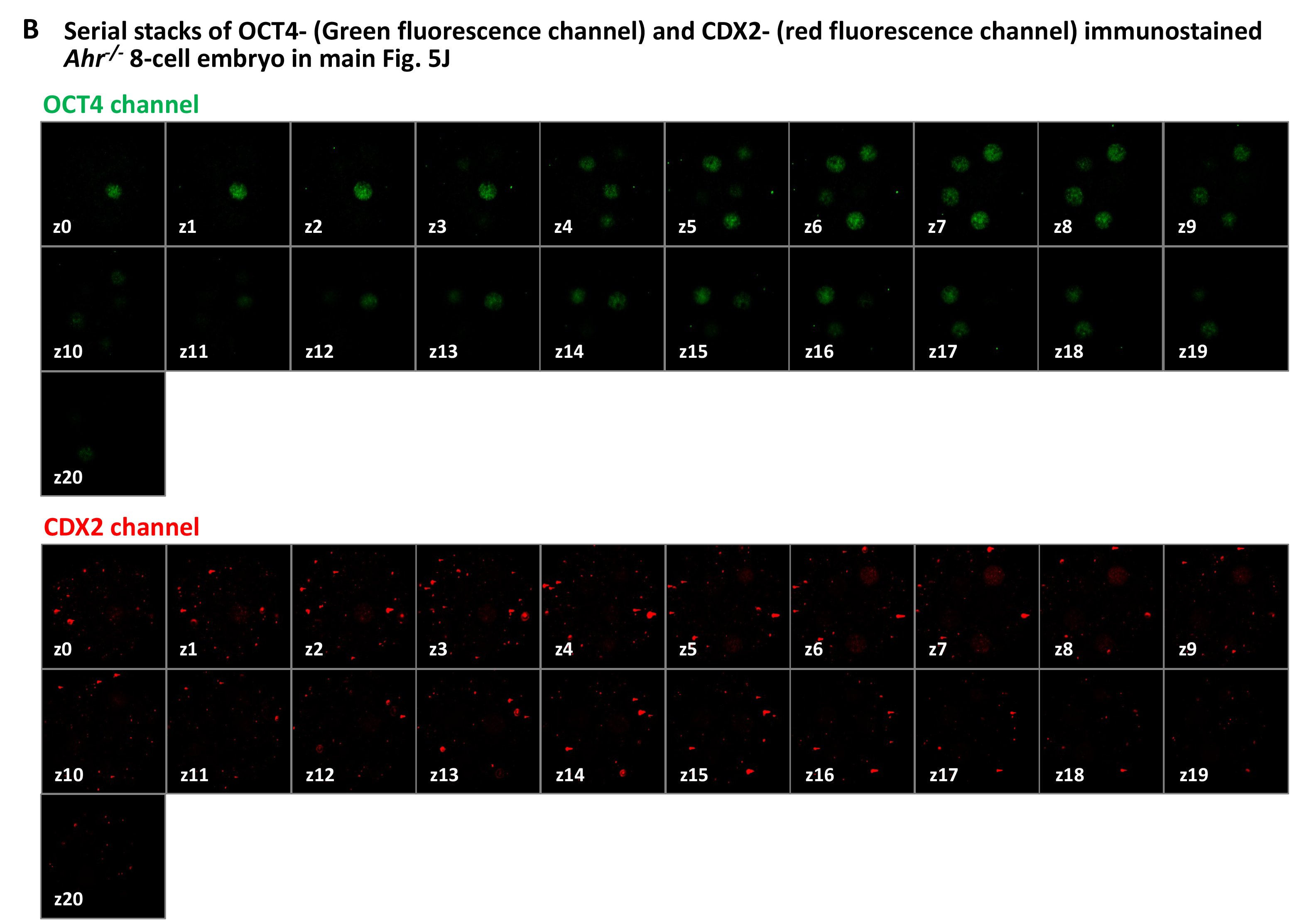


**Supplemental Fig. 3B.** Serial stacks of OCT4- (green fluorescent channel) and CDX2- (red fluorescent channel) immunostained *Ahr^-/-^* 8-cell embryo in main figure 5J.


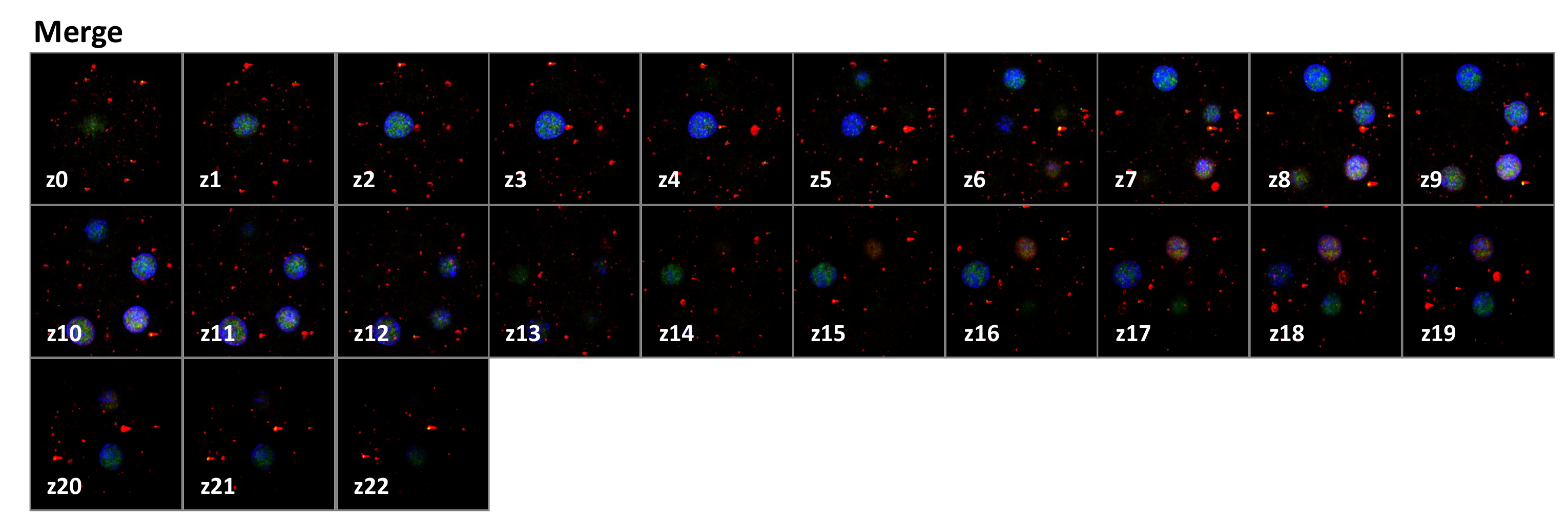

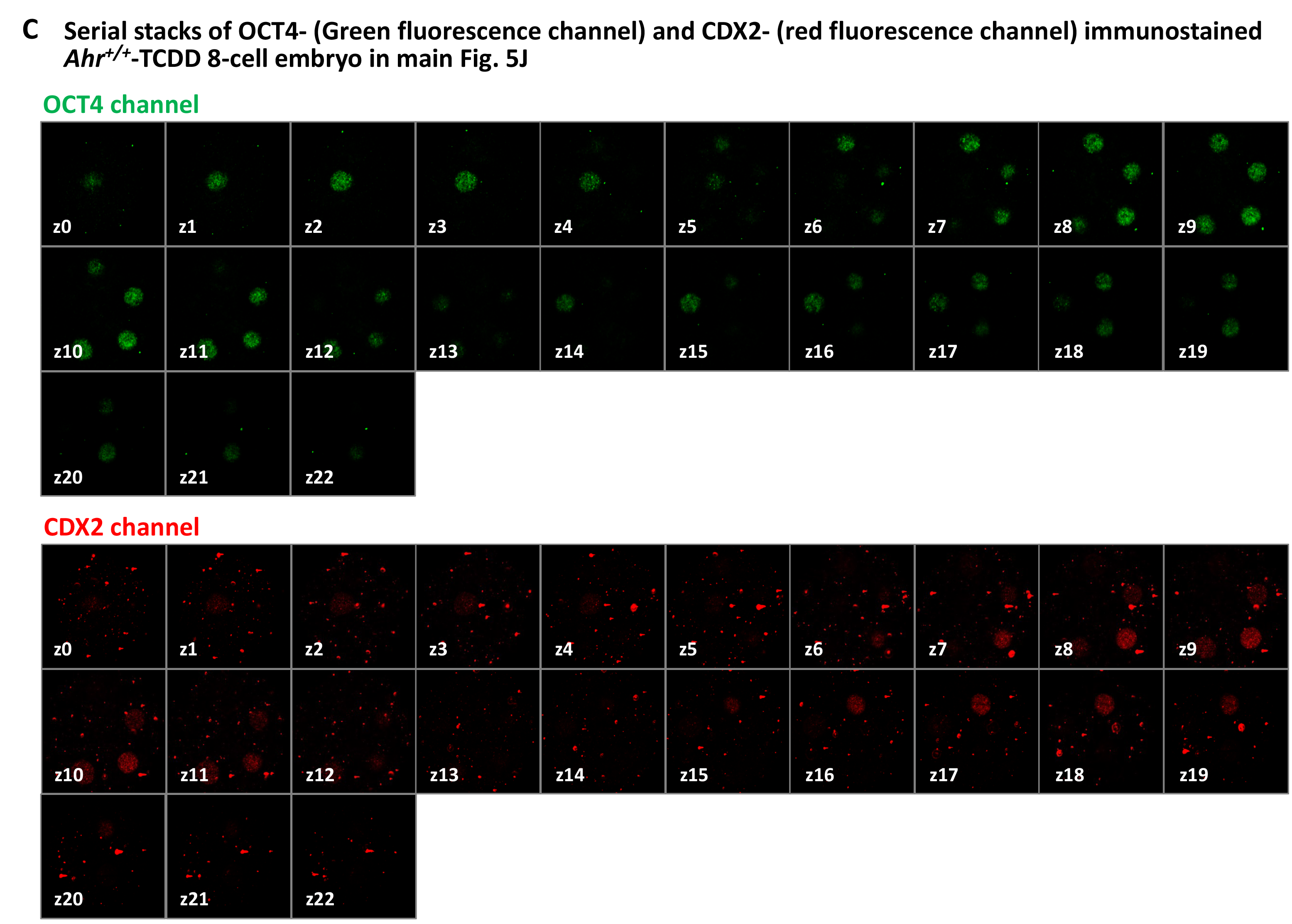


**Supplemental Fig. 3C.** Serial stacks of OCT4- (green fluorescent channel) and CDX2- (red fluorescent channel) immunostained *Ahr^+/+^*-TCDD 8-cell embryo in main figure 5J.


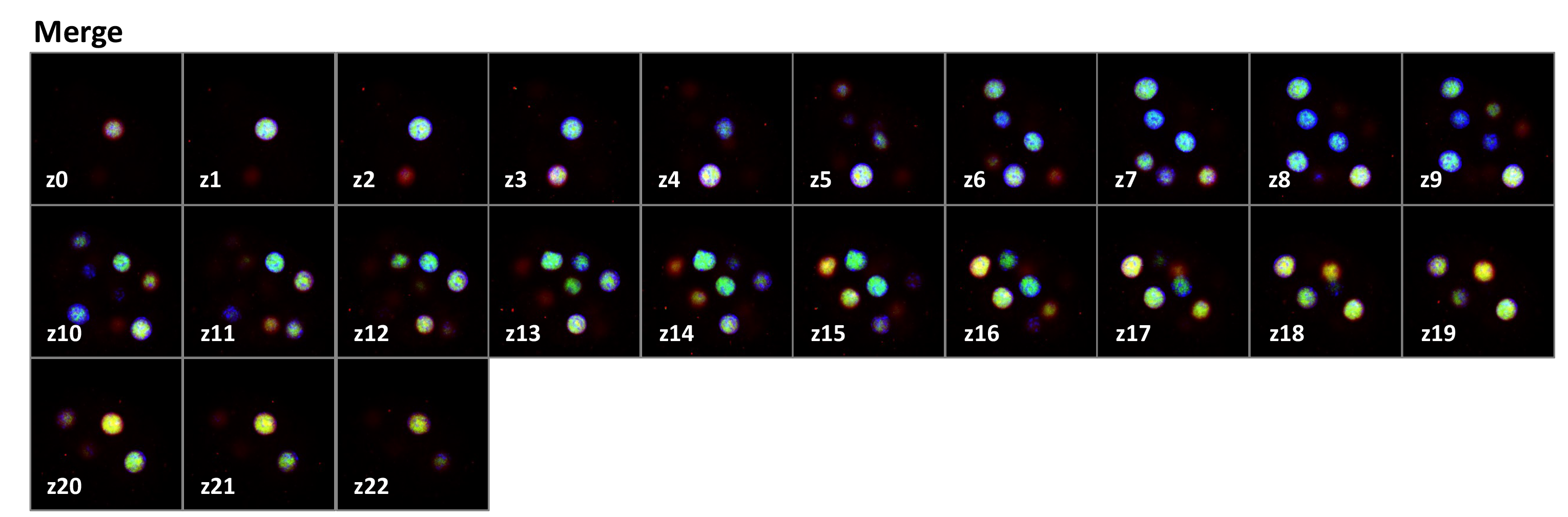

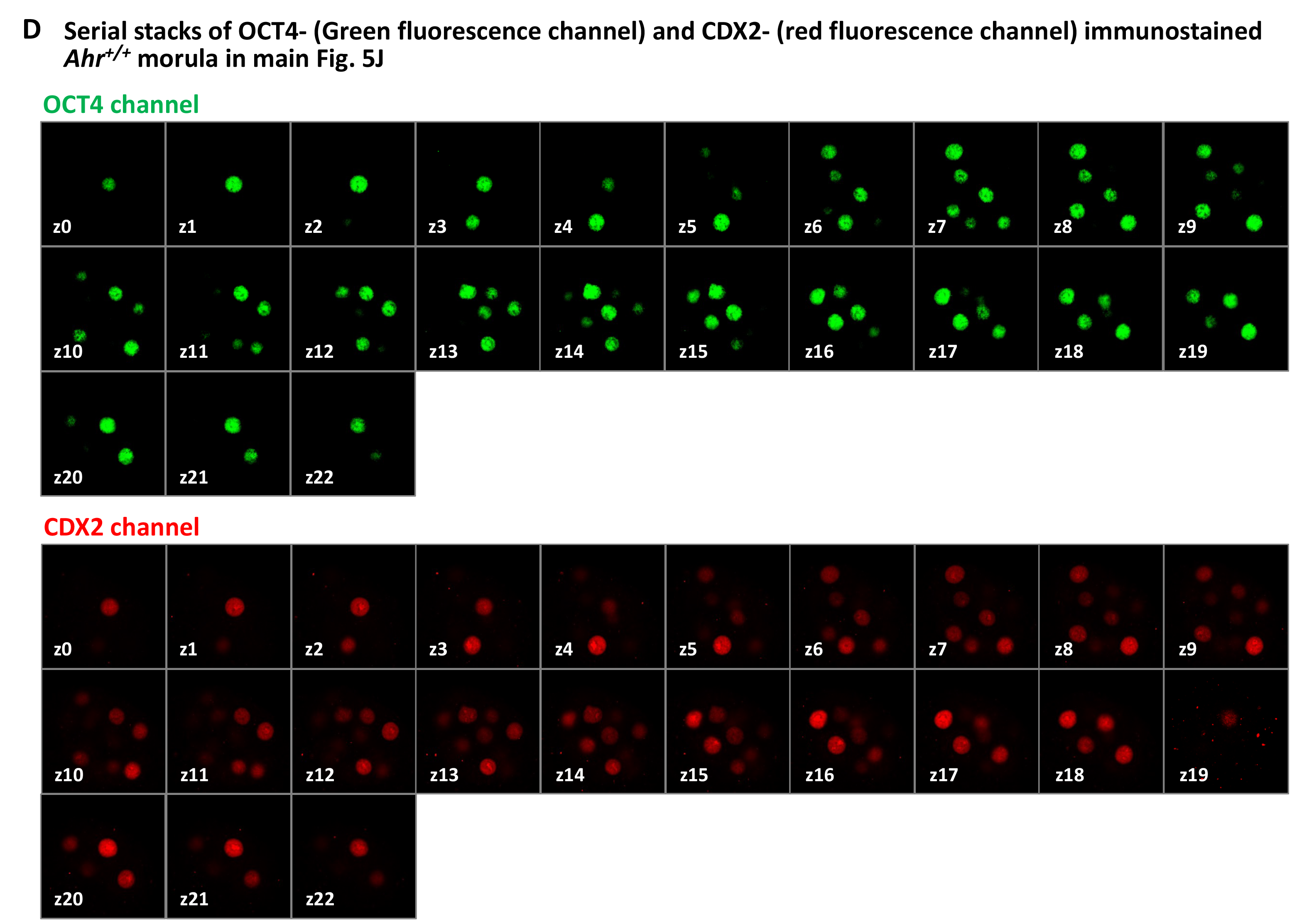


**Supplemental Fig. 3D.** Serial stacks of OCT4- (green fluorescent channel) and CDX2- (red fluorescent channel) immunostained *Ahr^+/+^* morula in main figure 5J.


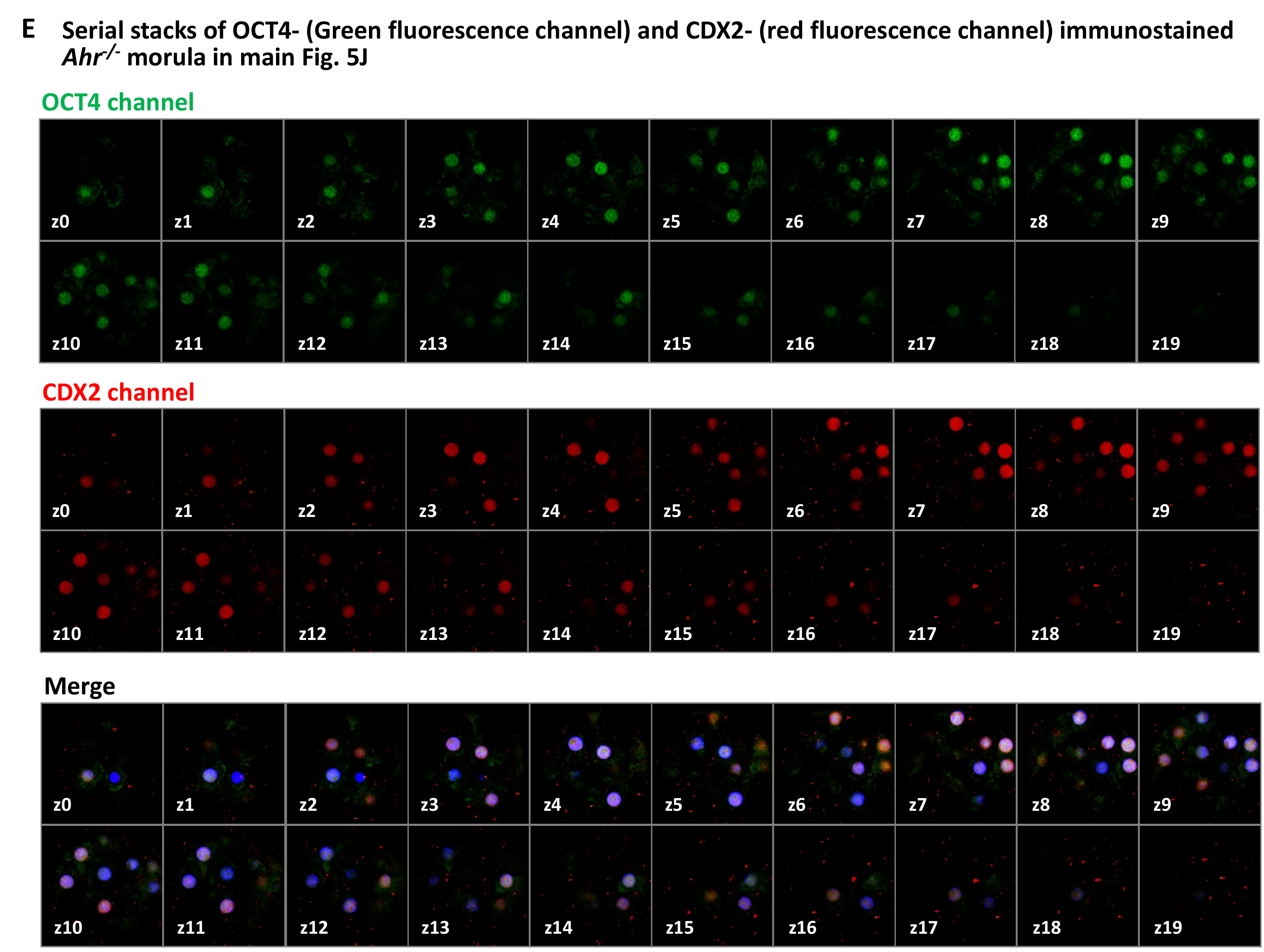


**Supplemental Fig. 3E.** Serial stacks of OCT4- (green fluorescent channel) and CDX2- (red fluorescent channel) immunostained *Ahr^-/-^* morula in main figure 5J.


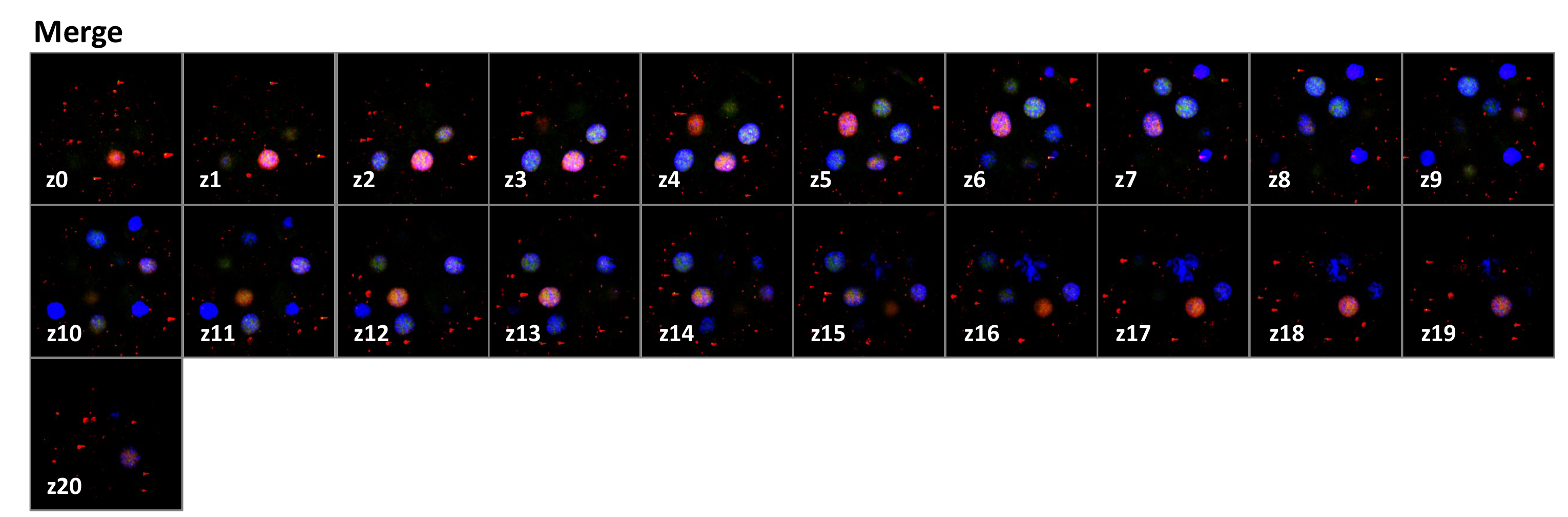

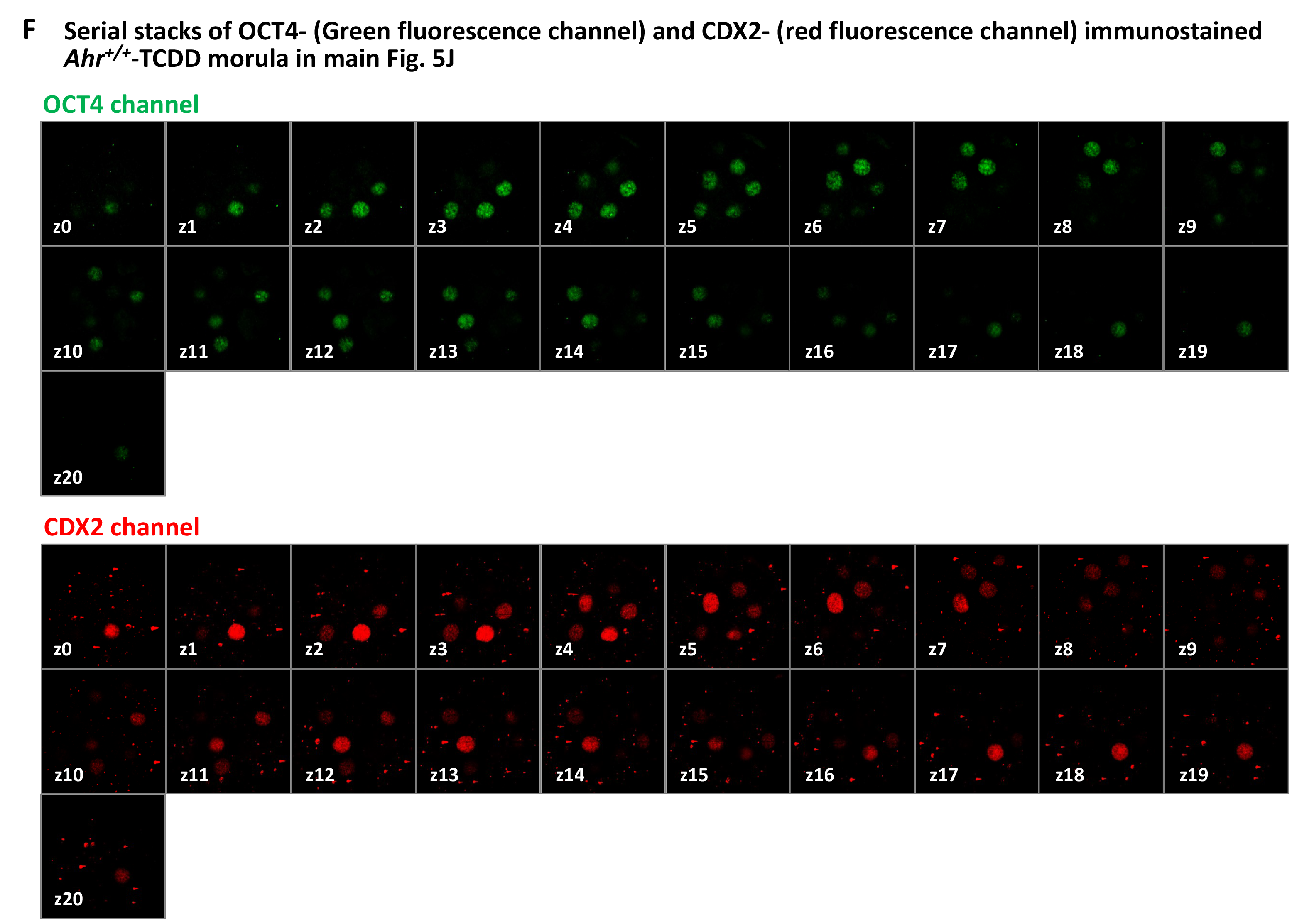


**Supplemental Fig. 3C.** Serial stacks of OCT4- (green fluorescent channel) and CDX2- (red fluorescent channel) immunostained *Ahr^+/+^*-TCDD morula in main figure 5J.

**Supplemental Figure 4. AHR Regulates the Transcriptional Heterogeneity.**


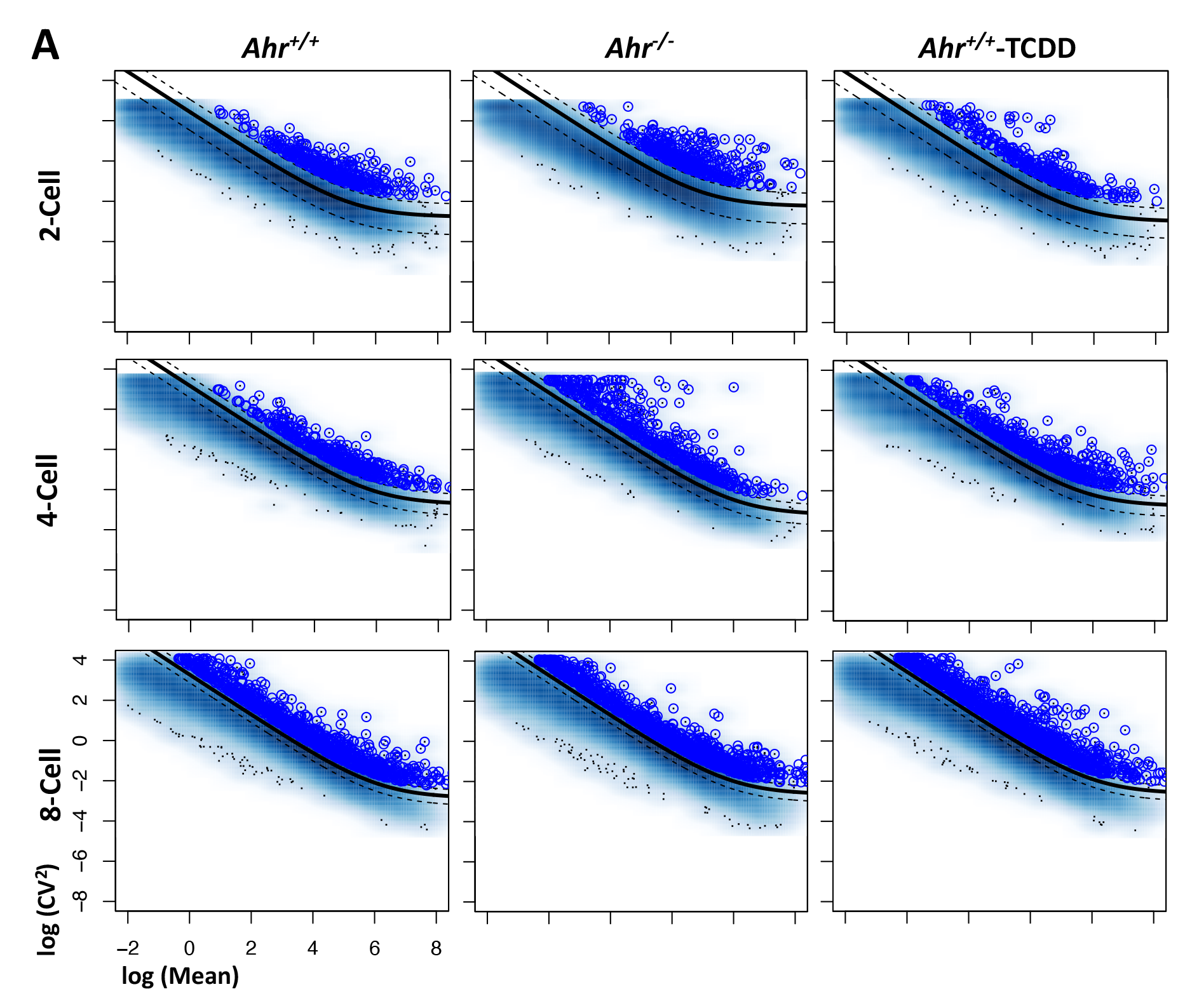


**Supplemental Fig. 4A.** Variability plots with genes displaying significant variable transcriptional expression level based on the parameter fitting the square of the coefficient of variance (CV^2^) and the average expression level. The solid line represents fitting variance-mean dependence and the dashed lines represent a 95% confidence interval for expected residual distribution.

**Supplemental Fig. 4B.** Variability plots showing variable genes labelled in blue and genes involved in AHR signaling in red. *p*-value obtained from Fisher’s exact test for significance is indicated.**
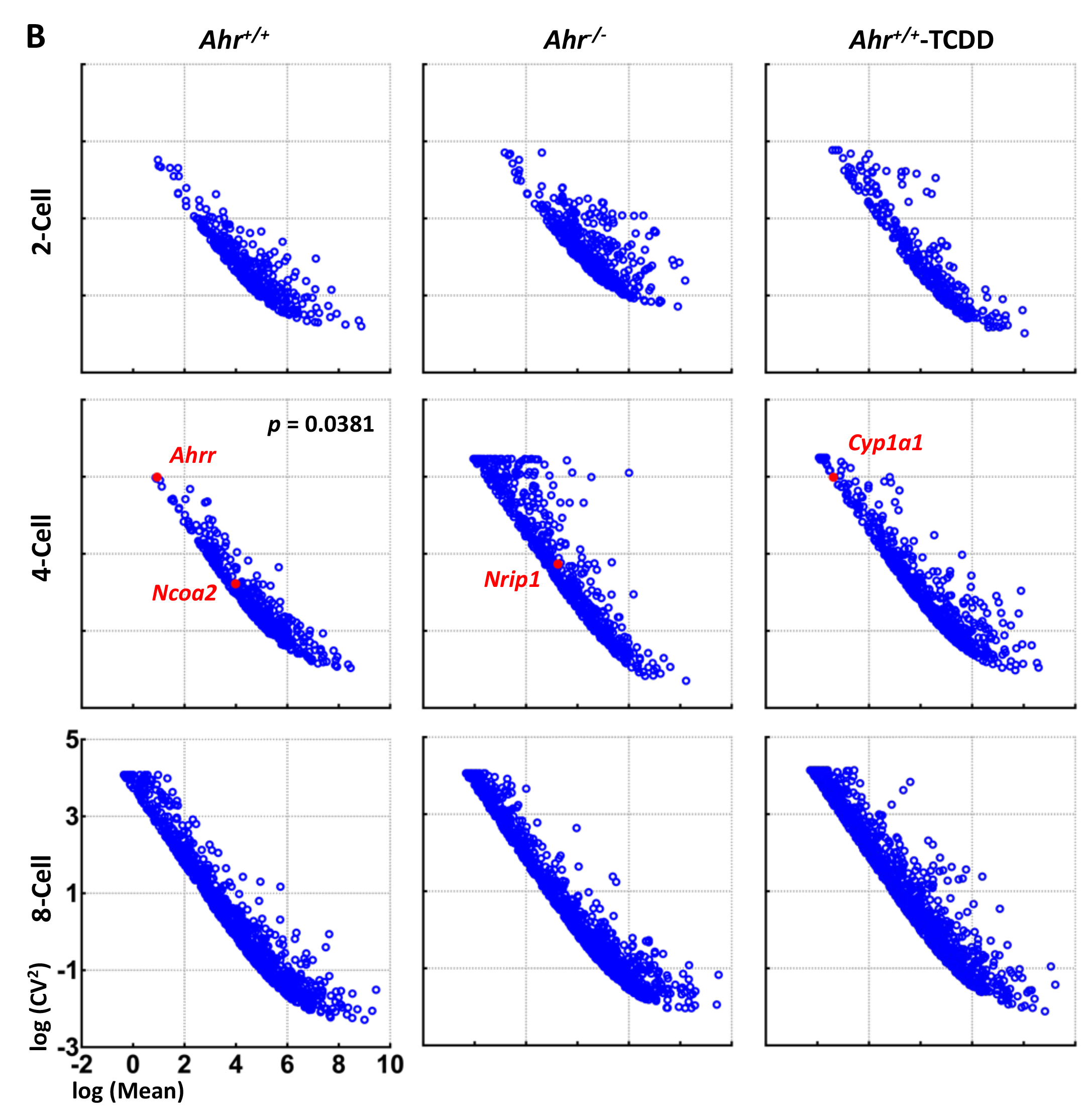
**

**Supplemental Fig. 4C.** Variability plots showing variable genes labelled in blue and genes involved in Role of OCT4 in mammalian ES pluripotency signaling in red.**
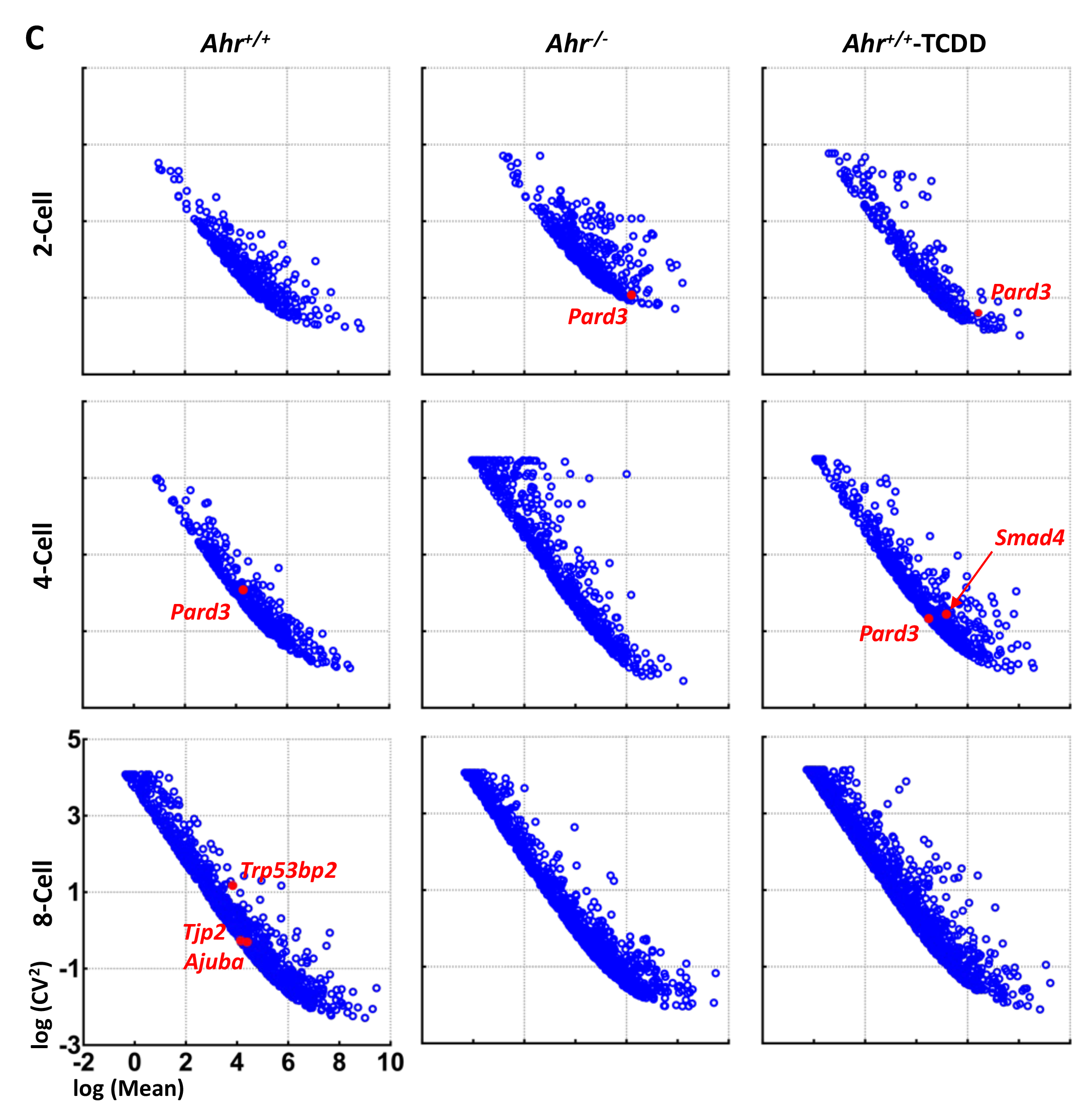
**

**Supplemental Fig. 4D.** Variability plots showing variable genes labelled in blue and genes involved in HIPPO signaling in red. *p*-values obtained from Fisher’s exact test for significance are indicated**
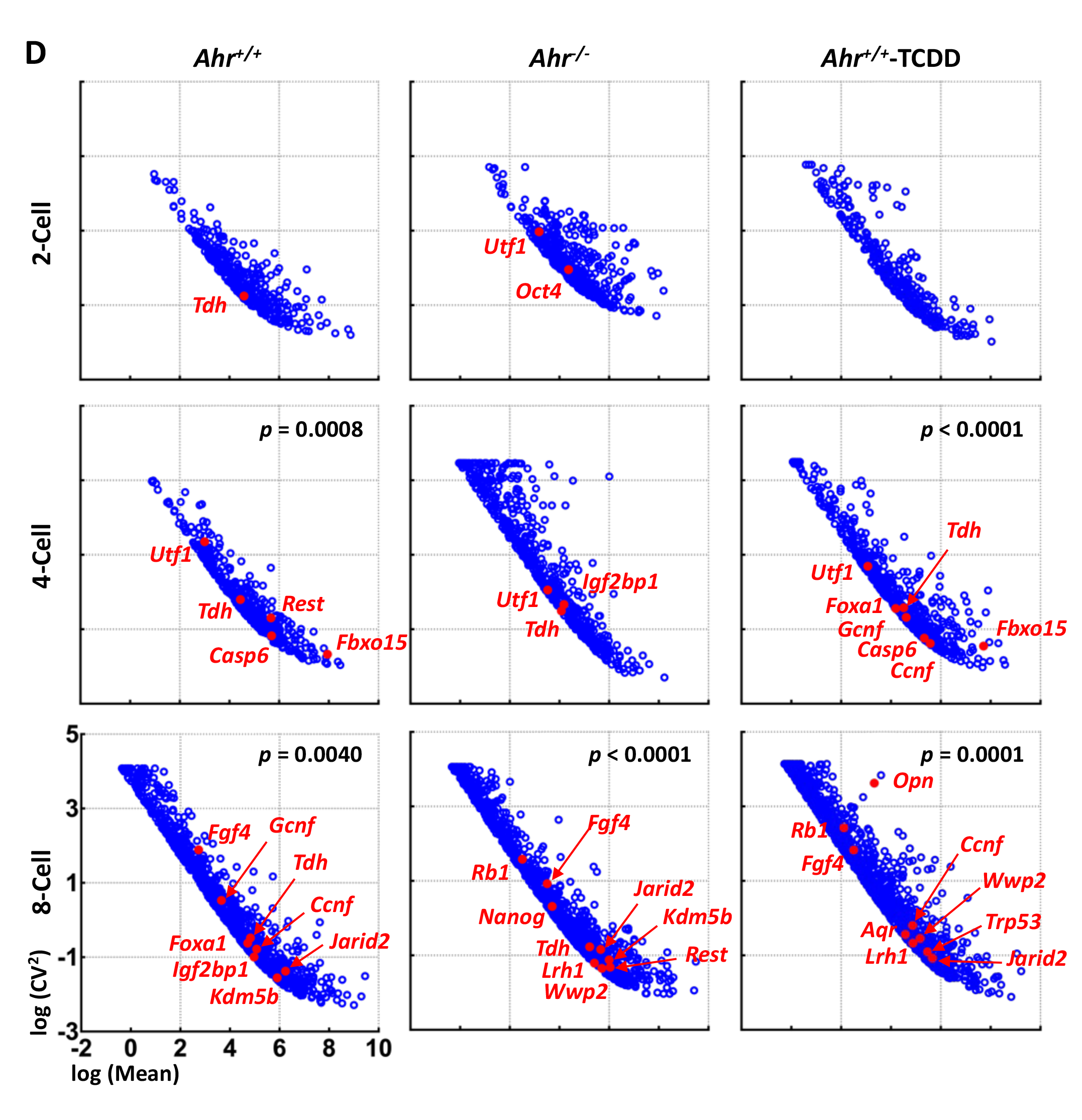
.**

**Supplemental Fig. 4E.** Variability plots showing variable genes labelled in blue and genes involved in mTOR signaling in red. *p*-values obtained from Fisher’s exact test for significance are indicated**
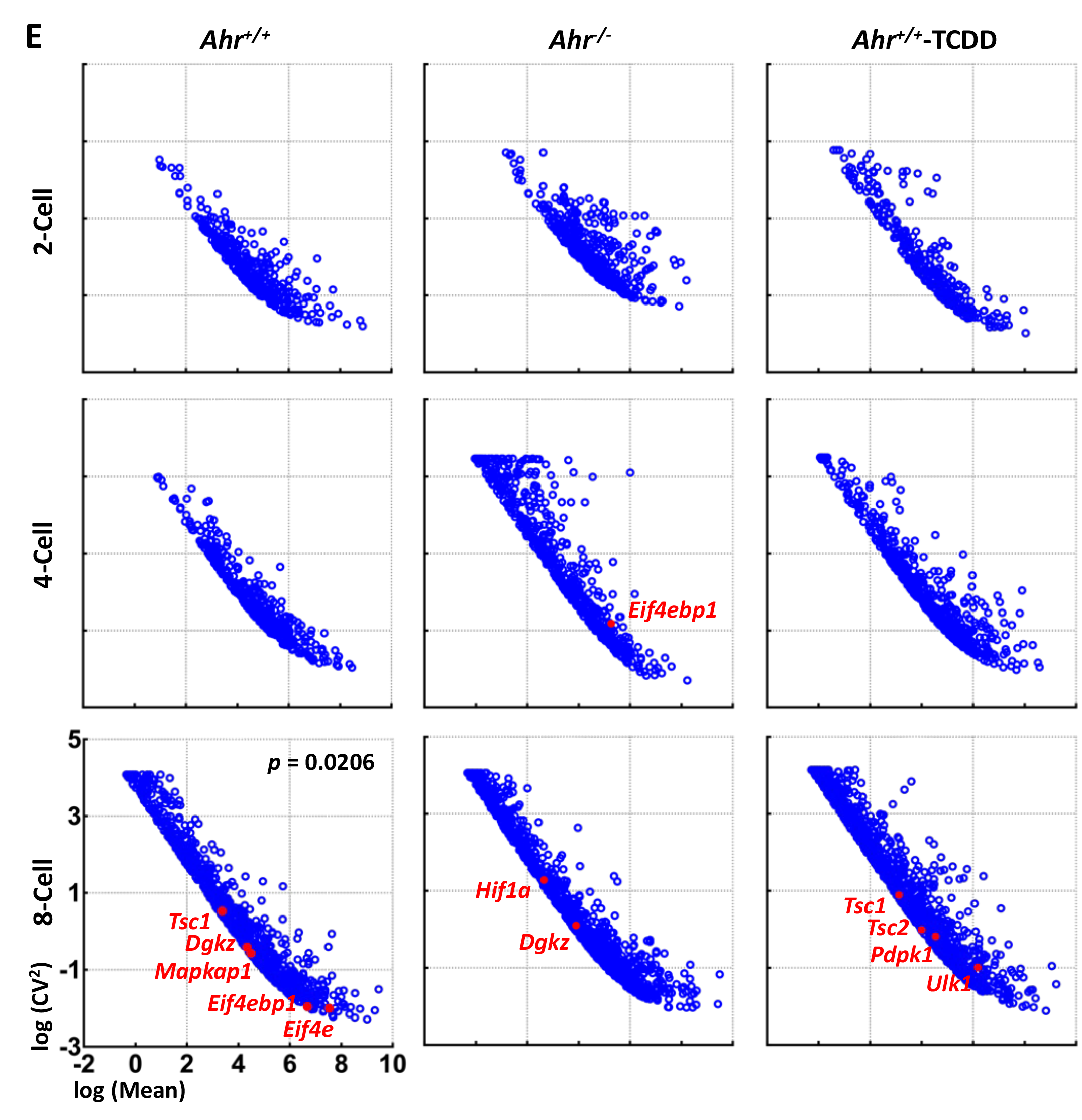
**.

**Supplemental Figure 5. AHR Regulates the Differentiation Trajectory of Progenitor Blastomeres.**


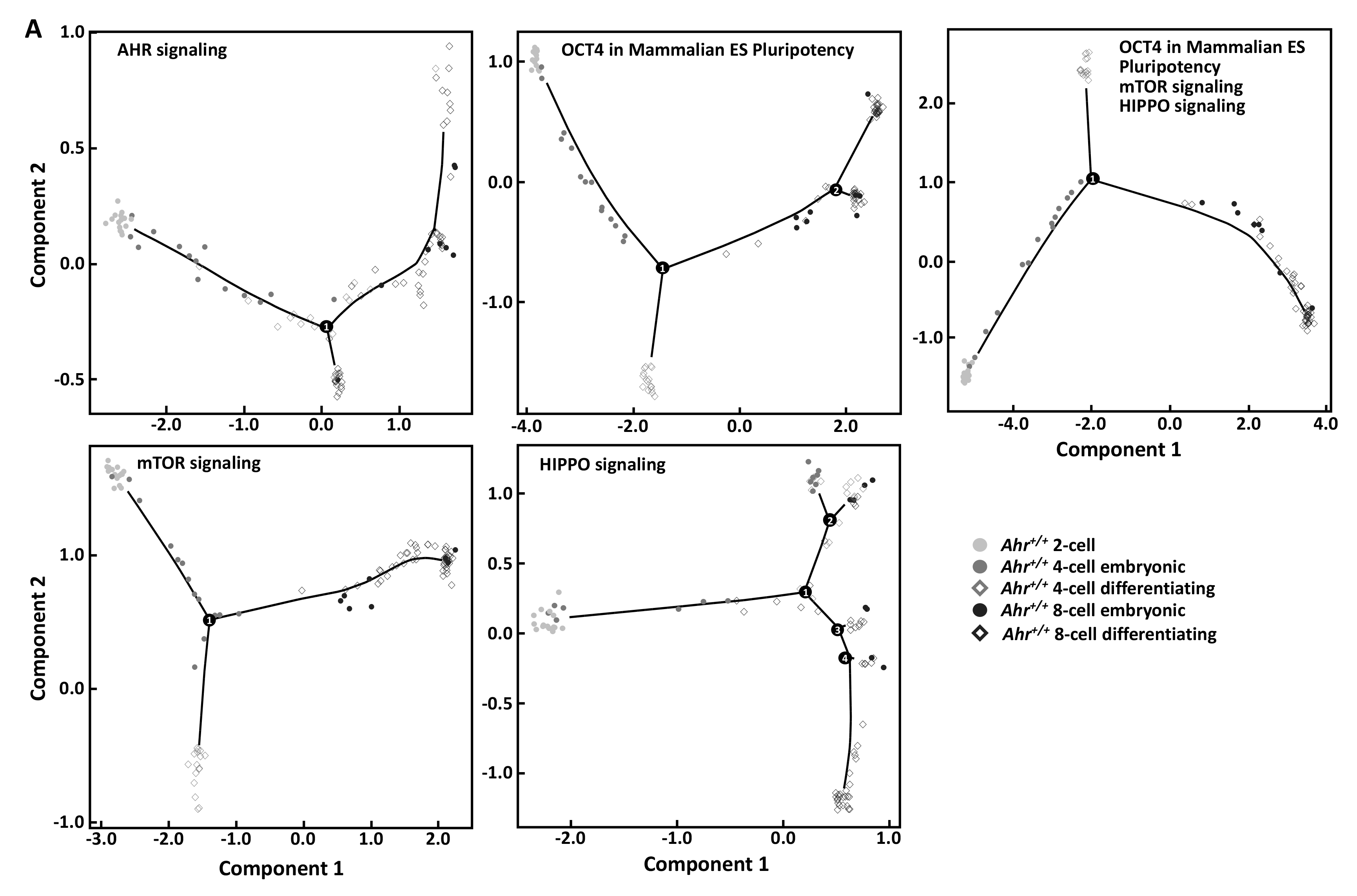


**Supplemental Fig. 5A.** Projection of trajectories using genes involved in AHR, Role of OCT4 in mammalian ES pluripotency, HIPPO signaling, mTOR signaling, and all three of OCT4, HIPPO, and mTOR signaling across *Ahr^+/+^* blastomeres.


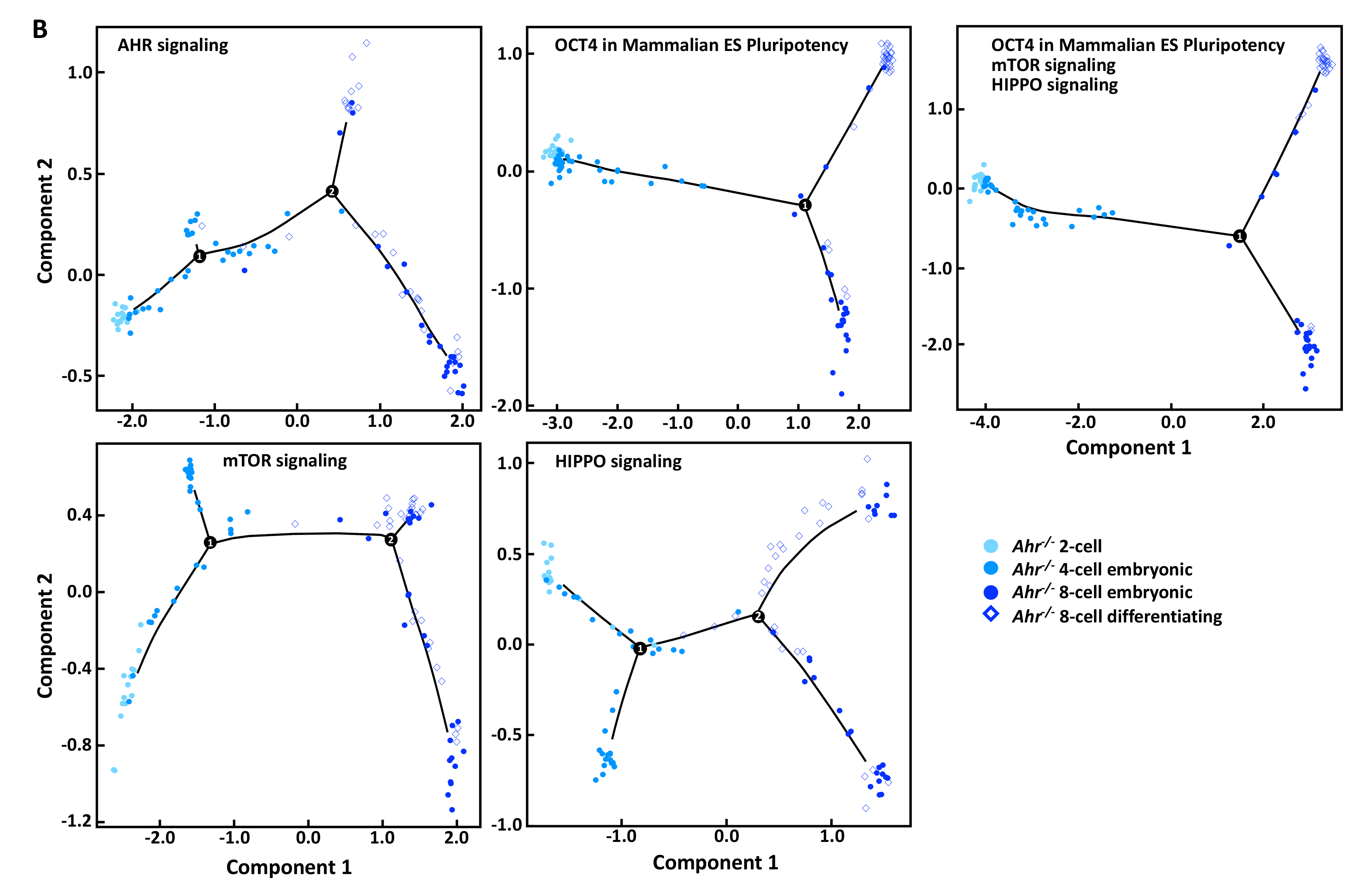


**Supplemental Fig. 5B.** Projection of trajectories using genes involved in AHR, Role of OCT4 in mammalian ES pluripotency, HIPPO signaling, mTOR signaling, and all three of OCT4, HIPPO, and mTOR signaling across *Ahr^-/-^* blastomeres.


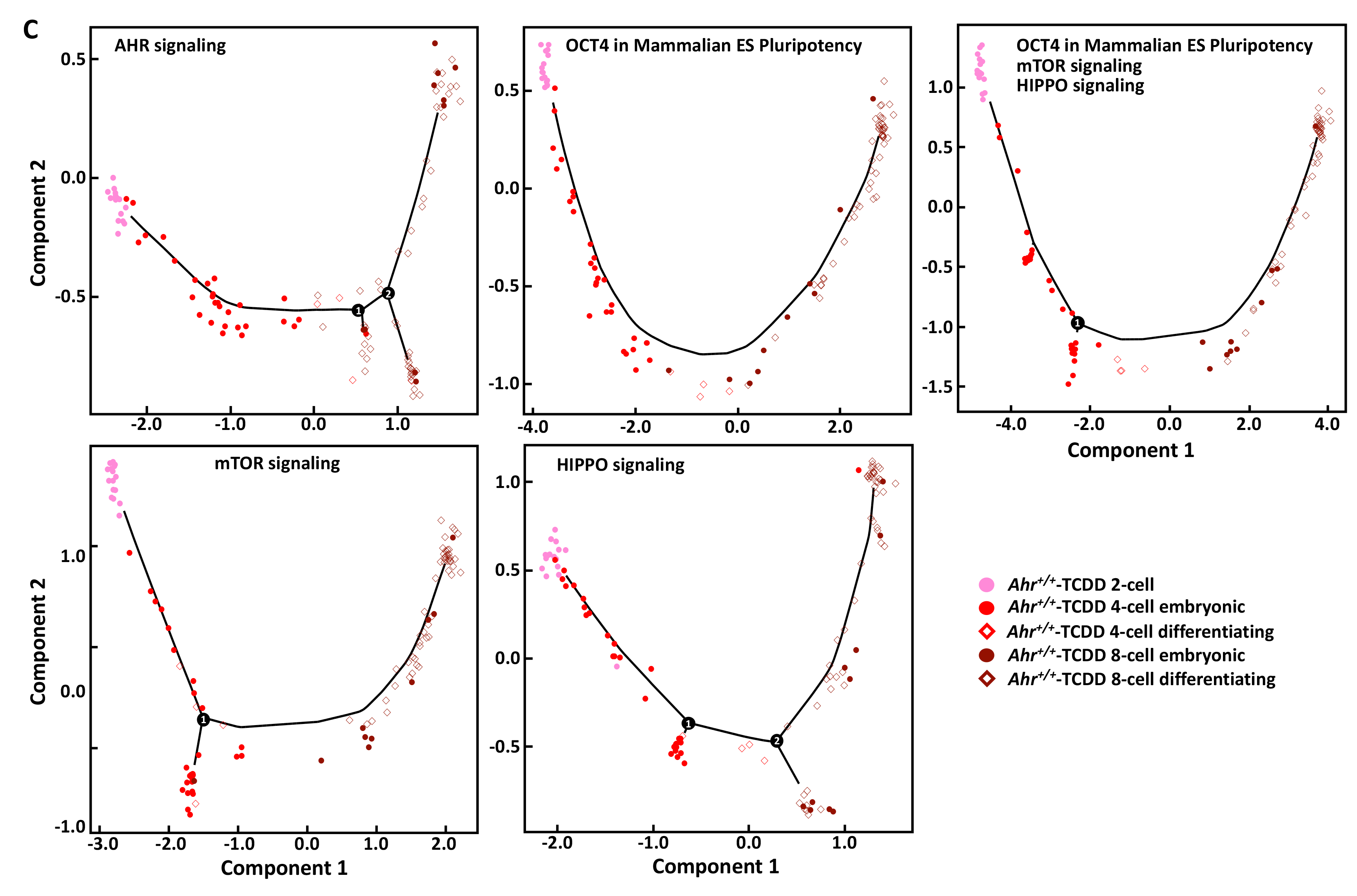


**Supplemental Fig. 5C.** Projection of trajectories using genes involved in AHR, Role of OCT4 in mammalian ES pluripotency, HIPPO signaling, mTOR signaling, and all three of OCT4, HIPPO, and mTOR signaling across *Ahr^+/+^*-TCDD blastomeres.


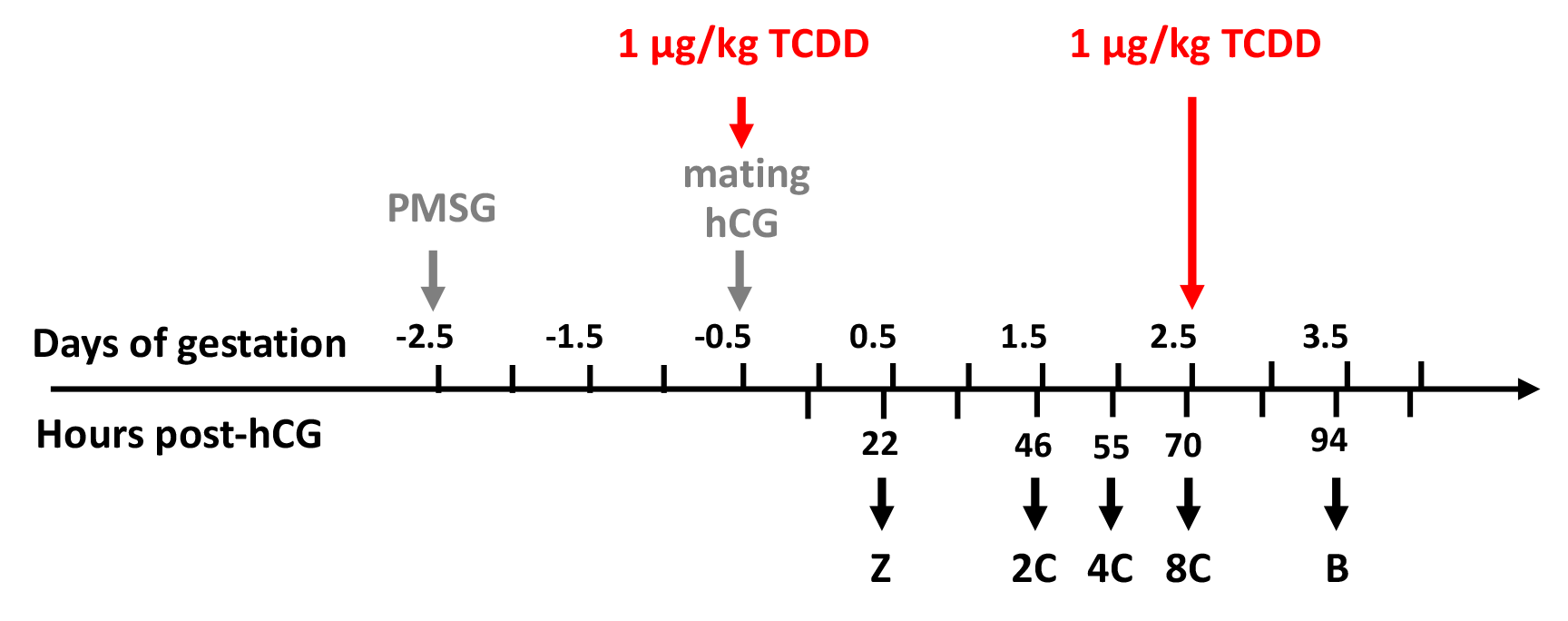


**Supplemental Figure 6. Experimental Scheme for Superovulation and Embryo Collection.** PMSG - pregnant mare serum gonadotrophin; hCG - human urine chorionic gonadotrophin; Z - zygotes; 2C, 4C, and 8C - 2-cell, 4-cell, and 8-cell embryos; B - blastocysts.

**Supplemental Figure 7. Example of scoring for pluripotency factor- and CDX2-expressing embryonic cells within blastocyst.**

**
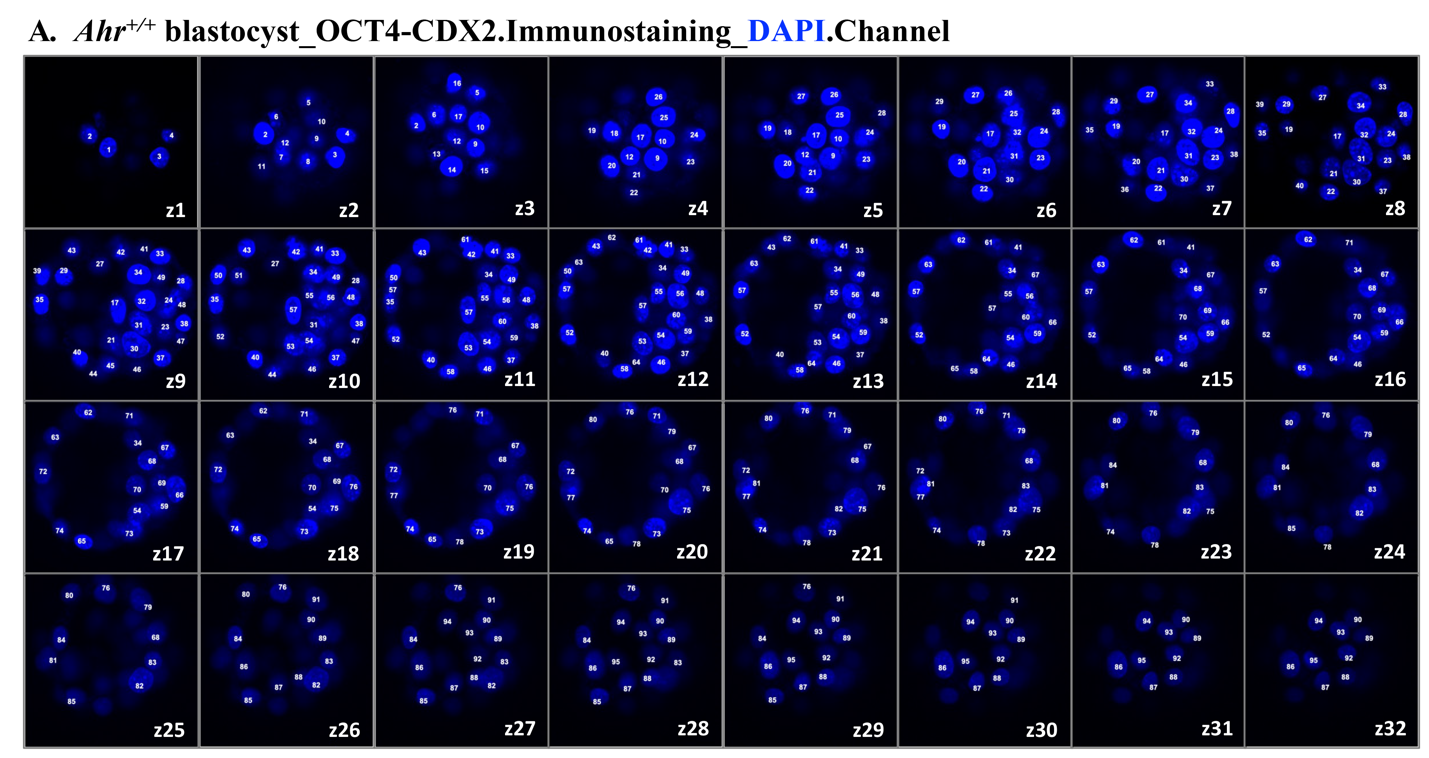
**

**
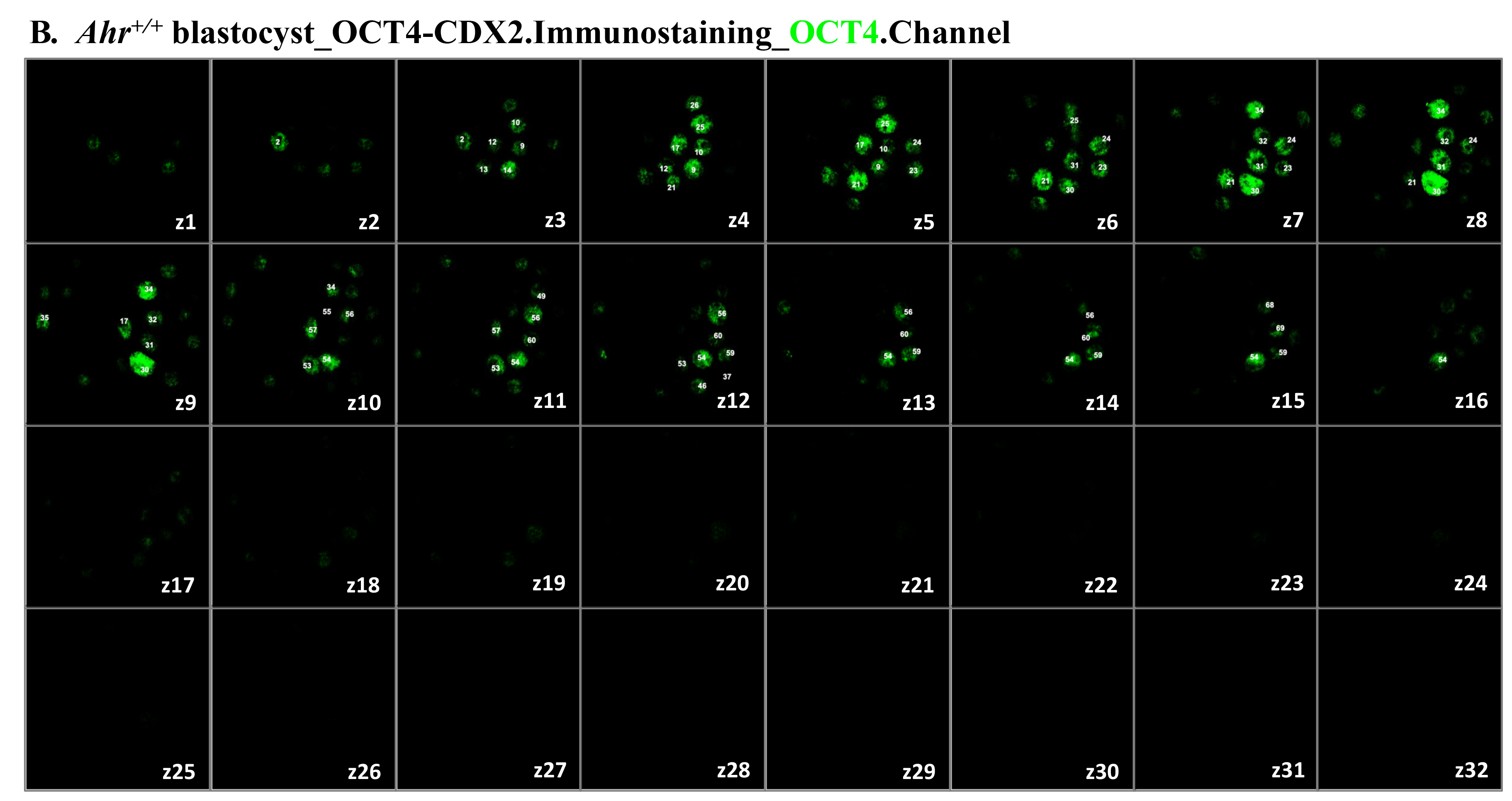
**

**
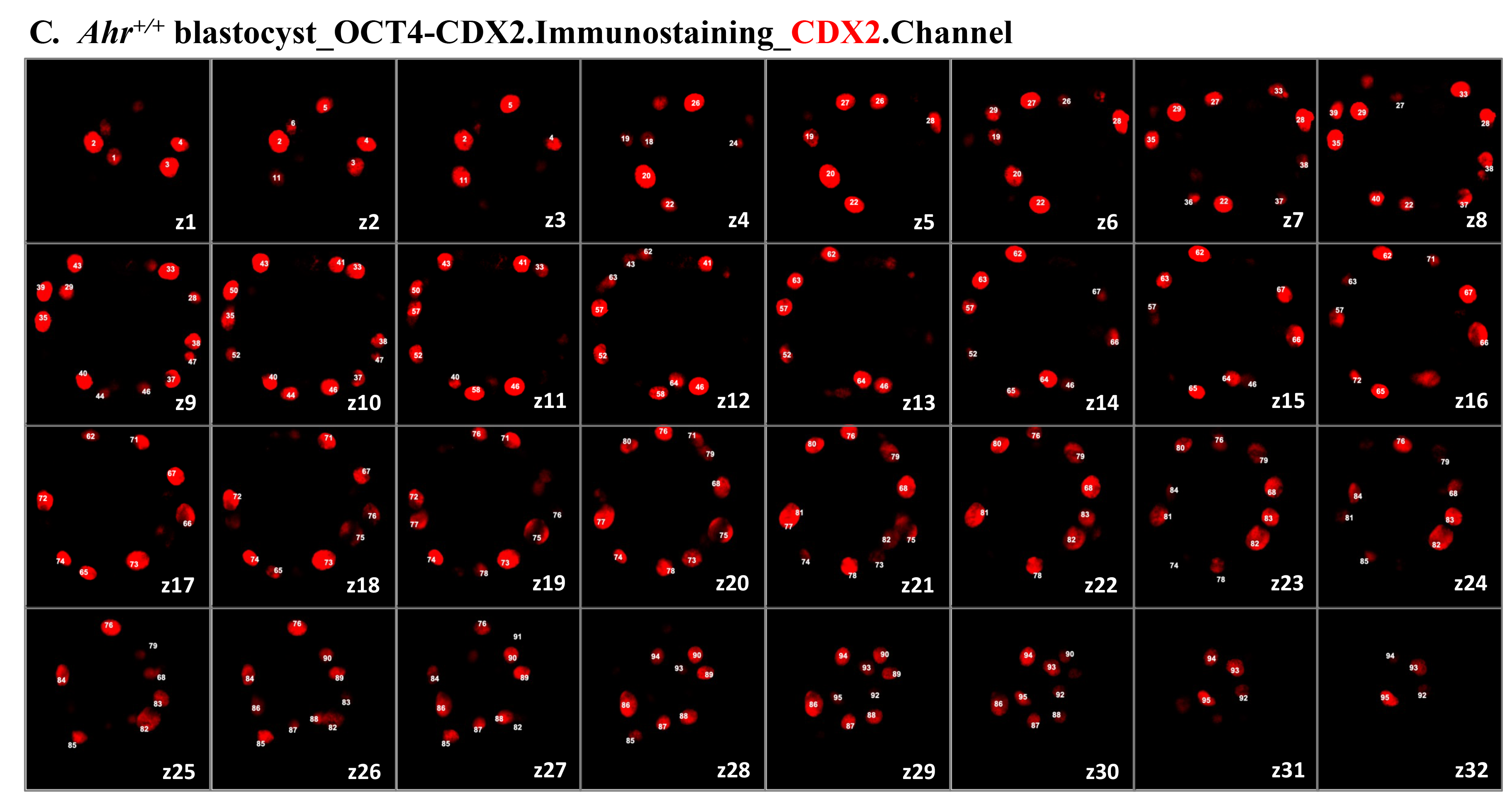
**
