# Supplementary material for: The aryl hydrocarbon receptor directs the differentiation of murine progenitor blastomeres
Source: Cell Biol Toxicol. 2022 Aug 27;39(4):1657–76. doi: 10.1007/s10565-022-09755-9 (PMC10425484; doi:10.1007/s10565-022-09755-9)
Supplement: Supplementary file 2 — Supplementary file2 (DOCX 35.1 KB) [file 10565_2022_9755_MOESM2_ESM.docx]

**Supplementary Table 1 - Assignment of single cell subpopulation**

| **Cell Seq. ID** | **Group** | **Subpopulation assignment** |
| --- | --- | --- |
| 1A1 | *Ahr^+/+^* 2-Cell | Embryonic |
| 1A2 | *Ahr^+/+^* 2-Cell | Embryonic |
| 1A3 | *Ahr^+/+^*-TCDD 2-Cell | Embryonic |
| 1A4 | *Ahr^+/+^*-TCDD 2-Cell | Embryonic |
| 1A5 | *Ahr^-/-^* 2-Cell | Embryonic |
| 1A6 | *Ahr^-/-^* 2-Cell | Embryonic |
| 1A7 | *Ahr^+/+^* 4-Cell | Embryonic |
| 1A8 | *Ahr^+/+^* 4-Cell | Embryonic |
| 1A9 | *Ahr^+/+^*-TCDD 4-Cell | Embryonic |
| 1A10 | *Ahr^+/+^*-TCDD 4-Cell | Embryonic |
| 1A11 | *Ahr^+/+^*-TCDD 4-Cell | Embryonic |
| 1A12 | *Ahr^+/+^*-TCDD 4-Cell | Embryonic |
| 1B1 | *Ahr^+/+^* 2-Cell | Embryonic |
| 1B2 | *Ahr^+/+^* 2-Cell | Embryonic |
| 1B3 | *Ahr^+/+^*-TCDD 2-Cell | Embryonic |
| 1B4 | *Ahr^+/+^*-TCDD 2-Cell | Embryonic |
| 1B5 | *Ahr^-/-^* 2-Cell | Embryonic |
| 1B6 | *Ahr^-/-^* 2-Cell | Embryonic |
| 1B7 | *Ahr^+/+^* 4-Cell | Embryonic |
| 1B8 | *Ahr^+/+^* 4-Cell | Embryonic |
| 1B9 | *Ahr^+/+^*-TCDD 4-Cell | Embryonic |
| 1B10 | *Ahr^+/+^*-TCDD 4-Cell | Embryonic |
| 1B11 | *Ahr^+/+^*-TCDD 4-Cell | Embryonic |
| 1B12 | *Ahr^+/+^*-TCDD 4-Cell | Embryonic |
| 1C1 | *Ahr^+/+^* 2-Cell | Embryonic |
| 1C2 | *Ahr^+/+^* 2-Cell | Embryonic |
| 1C3 | *Ahr^+/+^*-TCDD 2-Cell | Embryonic |
| 1C4 | *Ahr^+/+^*-TCDD 2-Cell | Embryonic |
| 1C5 | *Ahr^-/-^* 2-Cell | Embryonic |
| 1C6 | *Ahr^-/-^* 2-Cell | Embryonic |
| 1C7 | *Ahr^+/+^* 4-Cell | Embryonic |
| 1C8 | *Ahr^+/+^* 4-Cell | Embryonic |
| 1C9 | *Ahr^+/+^*-TCDD 4-Cell | Embryonic |
| 1C10 | *Ahr^+/+^*-TCDD 4-Cell | Embryonic |
| 1C11 | *Ahr^+/+^*-TCDD 4-Cell | Embryonic |
| 1C12 | *Ahr^+/+^*-TCDD 4-Cell | Embryonic |
| 1D1 | *Ahr^+/+^* 2-Cell | Embryonic |
| 1D2 | *Ahr^+/+^* 2-Cell | Embryonic |
| 1D3 | *Ahr^+/+^*-TCDD 2-Cell | Embryonic |
| **Cell Seq. ID** | **Group** | **Subpopulation assignment** |
| 1D4 | *Ahr^+/+^*-TCDD 2-Cell | Embryonic |
| 1D5 | *Ahr^-/-^* 2-Cell | Embryonic |
| 1D6 | *Ahr^-/-^* 2-Cell | Embryonic |
| 1D7 | *Ahr^+/+^* 4-Cell | Embryonic |
| 1D8 | *Ahr^+/+^* 4-Cell | Embryonic |
| 1D9 | *Ahr^+/+^*-TCDD 4-Cell | Differentiating |
| 1D11 | *Ahr^+/+^*-TCDD 4-Cell | Embryonic |
| 1D12 | *Ahr^+/+^*-TCDD 4-Cell | Embryonic |
| 1E1 | *Ahr^+/+^* 2-Cell | Embryonic |
| 1E2 | *Ahr^+/+^* 2-Cell | Embryonic |
| 1E3 | *Ahr^+/+^*-TCDD 2-Cell | Embryonic |
| 1E4 | *Ahr^+/+^*-TCDD 2-Cell | Embryonic |
| 1E5 | *Ahr^-/-^* 2-Cell | Embryonic |
| 1E6 | *Ahr^-/-^* 2-Cell | Embryonic |
| 1E7 | *Ahr^+/+^* 4-Cell | Embryonic |
| 1E8 | *Ahr^+/+^* 4-Cell | Embryonic |
| 1E9 | *Ahr^+/+^*-TCDD 4-Cell | Embryonic |
| 1E10 | *Ahr^+/+^*-TCDD 4-Cell | Embryonic |
| 1E11 | *Ahr^+/+^*-TCDD 4-Cell | Embryonic |
| 1E12 | *Ahr^+/+^*-TCDD 4-Cell | Embryonic |
| 1F1 | *Ahr^+/+^* 2-Cell | Embryonic |
| 1F2 | *Ahr^+/+^* 2-Cell | Embryonic |
| 1F3 | *Ahr^+/+^*-TCDD 2-Cell | Embryonic |
| 1F4 | *Ahr^+/+^*-TCDD 2-Cell | Embryonic |
| 1F5 | *Ahr^-/-^* 2-Cell | Embryonic |
| 1F6 | *Ahr^-/-^* 2-Cell | Embryonic |
| 1F7 | *Ahr^+/+^* 4-Cell | Embryonic |
| 1F8 | *Ahr^+/+^* 4-Cell | Embryonic |
| 1F9 | *Ahr^+/+^*-TCDD 4-Cell | Embryonic |
| 1F10 | *Ahr^+/+^*-TCDD 4-Cell | Embryonic |
| 1F11 | *Ahr^+/+^*-TCDD 4-Cell | Embryonic |
| 1F12 | *Ahr^+/+^*-TCDD 4-Cell | Embryonic |
| 1G2 | *Ahr^+/+^* 2-Cell | Embryonic |
| 1G3 | *Ahr^+/+^*-TCDD 2-Cell | Embryonic |
| 1G4 | *Ahr^+/+^*-TCDD 2-Cell | Embryonic |
| 1G6 | *Ahr^-/-^* 2-Cell | Embryonic |
| 1G7 | *Ahr^+/+^* 4-Cell | Embryonic |
| 1G9 | *Ahr^+/+^*-TCDD 4-Cell | Embryonic |
| 1G10 | *Ahr^+/+^*-TCDD 4-Cell | Embryonic |
| 1G11 | *Ahr^+/+^*-TCDD 4-Cell | Embryonic |
| **Cell Seq. ID** | **Group** | **Subpopulation assignment** |
| 1G12 | *Ahr^+/+^*-TCDD 4-Cell | Embryonic |
| 1H1 | *Ahr^+/+^* 2-Cell | Embryonic |
| 1H2 | *Ahr^+/+^* 2-Cell | Embryonic |
| 1H3 | *Ahr^+/+^*-TCDD 2-Cell | Embryonic |
| 1H4 | *Ahr^+/+^*-TCDD 2-Cell | Embryonic |
| 1H5 | *Ahr^-/-^* 2-Cell | Embryonic |
| 1H6 | *Ahr^-/-^* 2-Cell | Embryonic |
| 1H7 | *Ahr^+/+^* 4-Cell | Embryonic |
| 1H9 | *Ahr^+/+^*-TCDD 4-Cell | Embryonic |
| 1H10 | *Ahr^+/+^*-TCDD 4-Cell | Embryonic |
| 1H11 | *Ahr^+/+^*-TCDD 4-Cell | Embryonic |
| 2A1 | *Ahr^-/-^* 4-Cell | Embryonic |
| 2A2 | *Ahr^-/-^* 4-Cell | Embryonic |
| 2A3 | *Ahr^-/-^* 4-Cell | Embryonic |
| 2A4 | *Ahr^-/-^* 4-Cell | Embryonic |
| 2A5 | *Ahr^+/+^*-TCDD 4-Cell | Differentiating |
| 2A6 | *Ahr^+/+^* 8-Cell | Differentiating |
| 2A7 | *Ahr^+/+^* 8-Cell | Differentiating |
| 2A8 | *Ahr^+/+^*-TCDD 4-Cell | Differentiating |
| 2A9 | *Ahr^+/+^* 8-Cell | Differentiating |
| 2A10 | *Ahr^+/+^* 4-Cell | Differentiating |
| 2A11 | *Ahr^+/+^* 8-Cell | Differentiating |
| 2A12 | *Ahr^+/+^* 8-Cell | Differentiating |
| 2B1 | *Ahr^-/-^* 4-Cell | Embryonic |
| 2B2 | *Ahr^-/-^* 4-Cell | Embryonic |
| 2B3 | *Ahr^-/-^* 4-Cell | Embryonic |
| 2B4 | *Ahr^-/-^* 4-Cell | Embryonic |
| 2B5 | *Ahr^+/+^* 8-Cell | Differentiating |
| 2B6 | *Ahr^+/+^* 8-Cell | Differentiating |
| 2B7 | *Ahr^+/+^* 8-Cell | Differentiating |
| 2B8 | *Ahr^+/+^* 8-Cell | Differentiating |
| 2B9 | *Ahr^+/+^* 8-Cell | Embryonic |
| 2B10 | *Ahr^+/+^* 8-Cell | Differentiating |
| 2B11 | *Ahr^+/+^* 4-Cell | Differentiating |
| 2B12 | *Ahr^+/+^* 8-Cell | Differentiating |
| 2C1 | *Ahr^-/-^* 4-Cell | Embryonic |
| 2C2 | *Ahr^-/-^* 4-Cell | Embryonic |
| 2C3 | *Ahr^-/-^* 4-Cell | Embryonic |
| 2C4 | *Ahr^-/-^* 4-Cell | Embryonic |
| 2C5 | *Ahr^+/+^* 8-Cell | Differentiating |
| **Cell Seq. ID** | **Group** | **Subpopulation assignment** |
| 2C6 | *Ahr^+/+^* 8-Cell | N.A. |
| 2C7 | *Ahr^+/+^* 8-Cell | Differentiating |
| 2C8 | *Ahr^+/+^*-TCDD 4-Cell | Differentiating |
| 2C9 | *Ahr^+/+^* 8-Cell | Embryonic |
| 2C10 | *Ahr^+/+^* 8-Cell | Embryonic |
| 2C11 | *Ahr^+/+^* 8-Cell | Differentiating |
| 2C12 | *Ahr^+/+^* 8-Cell | Differentiating |
| 2D1 | *Ahr^-/-^* 4-Cell | Embryonic |
| 2D2 | *Ahr^-/-^* 4-Cell | Embryonic |
| 2D3 | *Ahr^-/-^* 4-Cell | Embryonic |
| 2D4 | *Ahr^-/-^* 4-Cell | Embryonic |
| 2D5 | *Ahr^+/+^* 8-Cell | Differentiating |
| 2D6 | *Ahr^+/+^* 8-Cell | Differentiating |
| 2D7 | *Ahr^+/+^* 8-Cell | Differentiating |
| 2D8 | *Ahr^+/+^* 8-Cell | Differentiating |
| 2D9 | *Ahr^+/+^* 8-Cell | Differentiating |
| 2D10 | *Ahr^+/+^* 8-Cell | N.A. |
| 2D11 | *Ahr^+/+^* 8-Cell | Differentiating |
| 2D12 | *Ahr^+/+^* 8-Cell | Differentiating |
| 2E1 | *Ahr^-/-^* 4-Cell | Embryonic |
| 2E2 | *Ahr^-/-^* 4-Cell | Embryonic |
| 2E3 | *Ahr^-/-^* 4-Cell | Embryonic |
| 2E4 | *Ahr^-/-^* 4-Cell | N.A. |
| 2E5 | *Ahr^+/+^* 8-Cell | Differentiating |
| 2E6 | *Ahr^+/+^* 8-Cell | Differentiating |
| 2E7 | *Ahr^+/+^* 8-Cell | Differentiating |
| 2E8 | *Ahr^+/+^* 8-Cell | Differentiating |
| 2E9 | *Ahr^+/+^* 8-Cell | Embryonic |
| 2E10 | *Ahr^+/+^* 8-Cell | Embryonic |
| 2E11 | *Ahr^+/+^* 8-Cell | Differentiating |
| 2E12 | *Ahr^+/+^* 8-Cell | Differentiating |
| 2F1 | *Ahr^-/-^* 4-Cell | Embryonic |
| 2F2 | *Ahr^-/-^* 4-Cell | Embryonic |
| 2F3 | *Ahr^-/-^* 4-Cell | Embryonic |
| 2F4 | *Ahr^-/-^* 4-Cell | Embryonic |
| 2F5 | *Ahr^+/+^* 8-Cell | Differentiating |
| 2F6 | *Ahr^+/+^* 8-Cell | Differentiating |
| 2F7 | *Ahr^+/+^* 8-Cell | Differentiating |
| 2F8 | *Ahr^+/+^* 8-Cell | Differentiating |
| 2F9 | *Ahr^+/+^* 8-Cell | Embryonic |
| **Cell Seq. ID** | **Group** | **Subpopulation assignment** |
| 2F10 | *Ahr^+/+^* 8-Cell | Differentiating |
| 2F11 | *Ahr^+/+^* 8-Cell | Differentiating |
| 2F12 | *Ahr^+/+^* 8-Cell | Differentiating |
| 2G1 | *Ahr^-/-^* 4-Cell | Embryonic |
| 2G2 | *Ahr^-/-^* 4-Cell | Embryonic |
| 2G3 | *Ahr^-/-^* 4-Cell | Embryonic |
| 2G4 | *Ahr^-/-^* 4-Cell | Embryonic |
| 2G5 | *Ahr^+/+^* 8-Cell | Differentiating |
| 2G6 | *Ahr^+/+^* 8-Cell | Differentiating |
| 2G7 | *Ahr^+/+^* 8-Cell | Differentiating |
| 2G8 | *Ahr^+/+^* 8-Cell | Embryonic |
| 2G9 | *Ahr^+/+^* 8-Cell | Differentiating |
| 2G10 | *Ahr^+/+^* 8-Cell | Differentiating |
| 2G11 | *Ahr^+/+^* 8-Cell | Differentiating |
| 2G12 | *Ahr^+/+^* 8-Cell | Differentiating |
| 2H1 | *Ahr^-/-^* 4-Cell | Embryonic |
| 2H2 | *Ahr^-/-^* 4-Cell | Embryonic |
| 2H3 | *Ahr^-/-^* 4-Cell | Embryonic |
| 2H4 | *Ahr^-/-^* 4-Cell | Embryonic |
| 2H5 | *Ahr^+/+^* 8-Cell | Differentiating |
| 2H6 | *Ahr^+/+^* 8-Cell | Differentiating |
| 2H7 | *Ahr^+/+^* 8-Cell | Differentiating |
| 2H8 | *Ahr^+/+^* 8-Cell | Embryonic |
| 2H9 | *Ahr^+/+^* 8-Cell | Differentiating |
| 2H10 | *Ahr^+/+^* 8-Cell | Differentiating |
| 2H11 | *Ahr^+/+^* 8-Cell | Differentiating |
| 2H12 | *Ahr^+/+^* 8-Cell | Differentiating |
| 3A1 | *Ahr^-/-^* 8-Cell | Differentiating |
| 3A2 | *Ahr^-/-^* 8-Cell | Embryonic |
| 3A4 | *Ahr^-/-^* 8-Cell | Embryonic |
| 3A5 | *Ahr^-/-^* 8-Cell | Differentiating |
| 3A6 | *Ahr^-/-^* 8-Cell | Differentiating |
| 3A7 | *Ahr^-/-^* 8-Cell | Differentiating |
| 3A9 | *Ahr^+/+^* 4-Cell | Differentiating |
| 3A10 | *Ahr^+/+^*-TCDD 8-Cell | Differentiating |
| 3A11 | *Ahr^+/+^*-TCDD 8-Cell | Differentiating |
| 3A12 | *Ahr^+/+^*-TCDD 8-Cell | Embryonic |
| 3B1 | *Ahr^-/-^* 8-Cell | Differentiating |
| 3B2 | *Ahr^-/-^* 8-Cell | Embryonic |
| 3B4 | *Ahr^-/-^* 8-Cell | Differentiating |
| **Cell Seq. ID** | **Group** | **Subpopulation assignment** |
| 3B5 | *Ahr^-/-^* 8-Cell | Differentiating |
| 3B6 | *Ahr^-/-^* 8-Cell | Differentiating |
| 3B7 | *Ahr^-/-^* 8-Cell | Differentiating |
| 3B9 | *Ahr^+/+^* 4-Cell | Differentiating |
| 3B10 | *Ahr^+/+^*-TCDD 8-Cell | Differentiating |
| 3B11 | *Ahr^+/+^*-TCDD 8-Cell | Embryonic |
| 3B12 | *Ahr^+/+^*-TCDD 8-Cell | Embryonic |
| 3C1 | *Ahr^-/-^* 8-Cell | Differentiating |
| 3C2 | *Ahr^-/-^* 8-Cell | Embryonic |
| 3C3 | *Ahr^-/-^* 8-Cell | Embryonic |
| 3C4 | *Ahr^-/-^* 8-Cell | Embryonic |
| 3C5 | *Ahr^-/-^* 8-Cell | Differentiating |
| 3C6 | *Ahr^-/-^* 8-Cell | Differentiating |
| 3C7 | *Ahr^-/-^* 8-Cell | Differentiating |
| 3C8 | *Ahr^+/+^* 4-Cell | Differentiating |
| 3C9 | *Ahr^+/+^* 4-Cell | Differentiating |
| 3C10 | *Ahr^+/+^*-TCDD 8-Cell | Differentiating |
| 3C11 | *Ahr^+/+^*-TCDD 8-Cell | Embryonic |
| 3C12 | *Ahr^+/+^*-TCDD 8-Cell | Differentiating |
| 3D1 | *Ahr^-/-^* 8-Cell | Differentiating |
| 3D2 | *Ahr^-/-^* 8-Cell | Embryonic |
| 3D3 | *Ahr^-/-^* 8-Cell | Embryonic |
| 3D4 | *Ahr^-/-^* 8-Cell | Embryonic |
| 3D5 | *Ahr^-/-^* 8-Cell | Differentiating |
| 3D6 | *Ahr^-/-^* 8-Cell | Differentiating |
| 3D7 | *Ahr^-/-^* 8-Cell | Differentiating |
| 3D8 | *Ahr^+/+^* 4-Cell | Differentiating |
| 3D9 | *Ahr^+/+^* 4-Cell | Differentiating |
| 3D10 | *Ahr^+/+^*-TCDD 8-Cell | Differentiating |
| 3D11 | *Ahr^+/+^*-TCDD 8-Cell | Embryonic |
| 3D12 | *Ahr^+/+^*-TCDD 8-Cell | Differentiating |
| 3E1 | *Ahr^-/-^* 8-Cell | Differentiating |
| 3E2 | *Ahr^-/-^* 8-Cell | Embryonic |
| 3E3 | *Ahr^-/-^* 8-Cell | Embryonic |
| 3E4 | *Ahr^-/-^* 8-Cell | Embryonic |
| 3E5 | *Ahr^-/-^* 8-Cell | Differentiating |
| 3E6 | *Ahr^-/-^* 8-Cell | Differentiating |
| 3E7 | *Ahr^-/-^* 8-Cell | Differentiating |
| 3E8 | *Ahr^+/+^* 4-Cell | Differentiating |
| 3E9 | *Ahr^+/+^* 4-Cell | Differentiating |
| **Cell Seq. ID** | **Group** | **Subpopulation assignment** |
| 3E10 | *Ahr^+/+^*-TCDD 8-Cell | Differentiating |
| 3E11 | *Ahr^+/+^*-TCDD 8-Cell | Embryonic |
| 3E12 | *Ahr^+/+^*-TCDD 8-Cell | Differentiating |
| 3F1 | *Ahr^-/-^* 8-Cell | Differentiating |
| 3F3 | *Ahr^-/-^* 8-Cell | Embryonic |
| 3F4 | *Ahr^-/-^* 8-Cell | Embryonic |
| 3F5 | *Ahr^-/-^* 8-Cell | Differentiating |
| 3F6 | *Ahr^-/-^* 8-Cell | Differentiating |
| 3F7 | *Ahr^-/-^* 8-Cell | Differentiating |
| 3F8 | *Ahr^+/+^* 4-Cell | Differentiating |
| 3F9 | *Ahr^+/+^* 4-Cell | Differentiating |
| 3F10 | *Ahr^+/+^*-TCDD 8-Cell | Differentiating |
| 3F11 | *Ahr^+/+^*-TCDD 8-Cell | Embryonic |
| 3F12 | *Ahr^+/+^*-TCDD 8-Cell | Differentiating |
| 3G1 | *Ahr^-/-^* 8-Cell | Differentiating |
| 3G3 | *Ahr^-/-^* 8-Cell | Embryonic |
| 3G4 | *Ahr^-/-^* 8-Cell | Embryonic |
| 3G5 | *Ahr^-/-^* 8-Cell | Differentiating |
| 3G6 | *Ahr^-/-^* 8-Cell | Differentiating |
| 3G7 | *Ahr^-/-^* 8-Cell | Differentiating |
| 3G8 | *Ahr^+/+^* 4-Cell | Differentiating |
| 3G9 | *Ahr^+/+^* 4-Cell | Differentiating |
| 3G10 | *Ahr^+/+^*-TCDD 8-Cell | Differentiating |
| 3G11 | *Ahr^+/+^*-TCDD 8-Cell | Embryonic |
| 3G12 | *Ahr^+/+^*-TCDD 8-Cell | Differentiating |
| 3H1 | *Ahr^-/-^* 8-Cell | Differentiating |
| 3H3 | *Ahr^-/-^* 8-Cell | Embryonic |
| 3H4 | *Ahr^-/-^* 8-Cell | Embryonic |
| 3H5 | *Ahr^-/-^* 8-Cell | Embryonic |
| 3H6 | *Ahr^-/-^* 8-Cell | Differentiating |
| 3H7 | *Ahr^-/-^* 8-Cell | N.A. |
| 3H8 | *Ahr^+/+^* 4-Cell | Differentiating |
| 3H9 | *Ahr^+/+^* 4-Cell | Differentiating |
| 3H10 | *Ahr^+/+^*-TCDD 8-Cell | Differentiating |
| 3H11 | *Ahr^+/+^*-TCDD 8-Cell | Embryonic |
| 3H12 | *Ahr^+/+^*-TCDD 8-Cell | Differentiating |
| 4A1 | *Ahr^+/+^*-TCDD 8-Cell | Differentiating |
| 4A2 | *Ahr^+/+^*-TCDD 8-Cell | Differentiating |
| 4A3 | *Ahr^+/+^*-TCDD 8-Cell | Differentiating |
| 4A4 | *Ahr^+/+^*-TCDD 8-Cell | Differentiating |
| **Cell Seq. ID** | **Group** | **Subpopulation assignment** |
| 4A5 | *Ahr^+/+^*-TCDD 8-Cell | Differentiating |
| 4A6 | *Ahr^-/-^* 8-Cell | Embryonic |
| 4B1 | *Ahr^+/+^*-TCDD 8-Cell | Differentiating |
| 4B2 | *Ahr^+/+^*-TCDD 8-Cell | Differentiating |
| 4B3 | *Ahr^+/+^*-TCDD 8-Cell | Embryonic |
| 4B4 | *Ahr^+/+^*-TCDD 8-Cell | Differentiating |
| 4B5 | *Ahr^+/+^*-TCDD 8-Cell | Differentiating |
| 4B6 | *Ahr^-/-^* 8-Cell | Embryonic |
| 4C1 | *Ahr^+/+^*-TCDD 8-Cell | Differentiating |
| 4C2 | *Ahr^+/+^*-TCDD 8-Cell | Differentiating |
| 4C3 | *Ahr^+/+^*-TCDD 8-Cell | Differentiating |
| 4C4 | *Ahr^+/+^*-TCDD 8-Cell | Differentiating |
| 4C5 | *Ahr^+/+^*-TCDD 8-Cell | Differentiating |
| 4C6 | *Ahr^-/-^* 8-Cell | Embryonic |
| 4D1 | *Ahr^+/+^*-TCDD 8-Cell | Differentiating |
| 4D2 | *Ahr^+/+^*-TCDD 8-Cell | Differentiating |
| 4D3 | *Ahr^+/+^*-TCDD 8-Cell | Differentiating |
| 4D4 | *Ahr^+/+^*-TCDD 8-Cell | Differentiating |
| 4D5 | *Ahr^+/+^*-TCDD 8-Cell | Differentiating |
| 4D6 | *Ahr^-/-^* 8-Cell | Embryonic |
| 4E1 | *Ahr^+/+^*-TCDD 8-Cell | Differentiating |
| 4E2 | *Ahr^+/+^*-TCDD 8-Cell | Differentiating |
| 4E3 | *Ahr^+/+^*-TCDD 8-Cell | Differentiating |
| 4E4 | *Ahr^+/+^*-TCDD 8-Cell | Differentiating |
| 4E5 | *Ahr^+/+^*-TCDD 8-Cell | Differentiating |
| 4E6 | *Ahr^-/-^* 8-Cell | Embryonic |
| 4F1 | *Ahr^+/+^*-TCDD 8-Cell | Differentiating |
| 4F2 | *Ahr^+/+^*-TCDD 8-Cell | N.A. |
| 4F3 | *Ahr^+/+^*-TCDD 8-Cell | Differentiating |
| 4F4 | *Ahr^+/+^*-TCDD 8-Cell | Differentiating |
| 4F5 | *Ahr^+/+^*-TCDD 8-Cell | Differentiating |
| 4F6 | *Ahr^-/-^* 8-Cell | Embryonic |
| 4G1 | *Ahr^+/+^*-TCDD 8-Cell | Differentiating |
| 4G2 | *Ahr^+/+^*-TCDD 8-Cell | Differentiating |
| 4G3 | *Ahr^+/+^*-TCDD 8-Cell | Differentiating |
| 4G4 | *Ahr^+/+^*-TCDD 8-Cell | Differentiating |
| 4G5 | *Ahr^+/+^*-TCDD 8-Cell | Differentiating |
| 4G6 | *Ahr^-/-^* 8-Cell | Embryonic |
| 4H1 | *Ahr^+/+^*-TCDD 8-Cell | Differentiating |
| 4H2 | *Ahr^+/+^*-TCDD 8-Cell | Differentiating |
| **Cell Seq. ID** | **Group** | **Subpopulation assignment** |
| 4H3 | *Ahr^+/+^*-TCDD 8-Cell | Differentiating |
| 4H4 | *Ahr^+/+^*-TCDD 8-Cell | Differentiating |
